# Simple inheritance of color and pattern polymorphism in the steppe grasshopper *Chorthippus dorsatus*

**DOI:** 10.1038/s41437-021-00433-w

**Published:** 2021-04-16

**Authors:** Gabe Winter, Mahendra Varma, Holger Schielzeth

**Affiliations:** 1 Population Ecology Group, Institute of Ecology and Evolution, Friedrich Schiller University Jena, Jena, Germany; 2 Max Planck Institute for Chemical Ecology, Jena, Germany

**Keywords:** Quantitative trait loci, Quantitative trait

## Abstract

The green–brown polymorphism of grasshoppers and bush-crickets represents one of the most penetrant polymorphisms in any group of organisms. This poses the question of why the polymorphism is shared across species and how it is maintained. There is mixed evidence for whether and in which species it is environmentally or genetically determined in Orthoptera. We report breeding experiments with the steppe grasshopper *Chorthippus dorsatus*, a polymorphic species for the presence and distribution of green body parts. Morph ratios did not differ between sexes, and we find no evidence that the rearing environment (crowding and habitat complexity) affected the polymorphism. However, we find strong evidence for genetic determination for the presence/absence of green and its distribution. Results are most parsimoniously explained by three autosomal loci with two alleles each and simple dominance effects: one locus influencing the ability to show green color, with a dominant allele for green; a locus with a recessive allele suppressing green on the dorsal side; and a locus with a recessive allele suppressing green on the lateral side. Our results contribute to the emerging contrast between the simple genetic inheritance of green–brown polymorphisms in the subfamily Gomphocerinae and environmental determination in other subfamilies of grasshoppers. In three out of four species of Gomphocerinae studied so far, the results suggest one or a few loci with a dominance of alleles allowing the occurrence of green. This supports the idea that brown individuals differ from green individuals by homozygosity for loss-of-function alleles preventing green pigment production or deposition.

## Introduction

The green–brown polymorphism in Orthoptera (grasshoppers and bush-crickets) represents one of the most widespread phenotypic polymorphisms among any group of organisms. The polymorphism has long been known (Dearn 1990; Rowell 1972) but seems underappreciated in contemporary literature. Among East African species of the family Acrididae, the green–brown polymorphism occurs in about 45% of the species (Rowell 1972), and among the about 1000 European Orthoptera species, it occurs in 30% of the species (Schielzeth 2020). The polymorphism is thus widely shared among species and is particularly common in species occurring in moist and alpine grasslands (Schielzeth 2020). It occurs in both major suborders of Orthoptera, Ensifera, and Caelifera, which have separated about 200 Mya (Misof et al. 2014), as well as in species of Mantodea, Phasmatodea, and Mantophasmatodea, thus in the wider sibship of polyneopteran insects.

The widespread co-occurrence of multiple color morphs poses the question of how such conspicuous intra-specific diversity is maintained and whether the developmental processes are shared across species (Jamie and Meier 2020; Orteu and Jiggins 2020). Recent data on color polymorphic stick insects suggest that causal loci are shared among different species from the same genus (Comeault et al. 2015; Villoutreix et al. 2020). For other groups of Polyneoptera, the loci have not yet been mapped. For most species, it is not even clear whether the green–brown polymorphisms are environmentally or genetically determined. This lack of basic knowledge currently precludes an evaluation of why the polymorphism is shared across species.

Polymorphisms might, in principle, be transient or balanced (Ford 1966). For the green–brown system in Orthoptera, the polymorphism seems to be balanced rather than transient since there is neither indication for widespread directional shifts in morph ratios nor indications of extensive admixture of divergent populations (Schielzeth 2020). However, some geographic patterns, such as increases of brown morphs with altitude (Köhler et al. 2017) and spatial heterogeneity in morph ratios (Dearn 1990; Dieker et al. 2018; Gill 1979) suggest habitat-specific selection. In order to understand how the polymorphism is maintained and why it is shared across species, it is important to know how it is formed during development since genetic and environmental control will evolve very differently. Current evidence is mixed in this respect.

There are several Orthoptera species in which the development of brown or green phenotypes is induced by the environment. Phenotypic plasticity in the green–brown polymorphism has been demonstrated for multiple species of the subfamilies Cyrtacanthacridinae (Rowell and Cannis 1971; Tanaka 2004; Tanaka et al. 2012), Oedipodinae (Ergene 1955; Rowell 1970), and Acridinae (Ergene 1950, 1952a, b; Okay 1956). High humidity tends to favor green, high temperature and high population density favor brown, and substrate color favors matches (Dearn 1990; Rowell 1972). Color changes in these species seem to work in both directions (green to brown and brown to green) and are typically associated with molt. Such results have led to the belief that orthopterans are generally phenotypically plastic for the green–brown polymorphism. However, this is not universally true. In *Conocephalus maculatus* (one of the few Ensifera species studied), for instance, the development into green or brown imagoes (from universally green nymphae) depends on parental morphs, and not on temperature, humidity, or substrate color (Oda and Ishii 1998, 2001). Furthermore, results on species from the subfamily Gomphocerinae suggest a simple genetic mechanism, as we show and discuss below.

The Gomphocerinae represents one of the largest subfamilies of grasshoppers, with more than 1200 species worldwide. Unlike phenotypically plastic species from other subfamilies, species of Gomphocerinae do not seem to respond to environmental triggers (Helfert 1978; Valverde and Schielzeth 2015). An exception is a study on a North American gomphocerine grasshopper, which reports tendencies to produce more brown morphs under low humidity or when fed on dry grass (Otte and Williams 1972). And it has been found in the field that morph ratios change throughout the season in a Central American species of Gomphocerinae (Lecoq and Pierozzi 1996). However, these patterns might be caused by phenological differences or differential mortality rather than phenotypic plasticity. In contrast to the limited evidence for environmental color determination in Gomphocerine, there is strong evidence that the green color is heritable and controlled by simple Mendelian loci in three different species (Gill 1981; Sansome and La Cour 1935; Schielzeth and Dieker 2020) (Table 1). We here present new data on the inheritance of color morphs in another species of Gomphocerinae and summarize published data in light of the green–brown determination.

**Table 1:** Overview of studies on environmental versus genetic determination of the green–brown color polymorphism in grasshoppers of the subfamily Gomphocerinae (Orthoptera, Acrididae).

Species	Experimental design	Phenotypic plasticity	Genetic control	Reference
*Syrbula admirabilis*	346 field-caught individuals raised under manipulated dryness of food and humidity	Increased proportion of brown individuals when raised on dry grass or at low humidity	Not tested	Otte and Williams (1972)
*Chorthippus biguttulus*	218 field-caught individuals raised under manipulated substrate, temperature, and humidity	Green not affected by substrate color, temperature, or humidity (while darkness was affected)	Not tested	Helfert (1978)
*Chorthippus brunneus*	789 offspring from 41 experimental crosses in standard cages	Not tested	93% green in green–green matings, 61% green in mixed matings, 0% green in brown–brown matings, suggesting an autosomal locus with a dominant green allele	Gill (1981)
*Gomphocerus sibiricus*	(1) 78 individuals of mixed ancestry in manipulated environments, (2) 404 offspring from 89 experimental crosses in standard cages	(1) Lack of response to manipulated substrate color and radiation intensity (temperature)	(2) 83% green in green–green matings, 55% green in mixed matings, 3% green in brown–brown matings suggesting a major autosomal locus with dominant green allele	(1) Valverde and Schielzeth (2015), (2) Schielzeth and Dieker (2020)
*Pseudochorthippus parallelus*	(1) 1850 offspring from 96 experimental crosses, (2) 549 offspring from 7 mating combinations in standard cages (multiple pairs pooled)	Not tested	(1) 100% green in green–green matings, 47% green in mixed matings, 26% green in brown–brown matings suggesting a major autosomal locus with dominant brown allele, (2) 90% green in green–green matings, 78% green in mixed matings, 18% green in brown–brown matings suggesting heritable, but more complex inheritance	(1) Sansome and La Cour (1935), (2) Köhler (2006)

In many species of gomphocerine grasshoppers, green colors are variably distributed across different parts of the body. Besides variation in the exact extent and intensity of green, as well as variable concealment of green in some areas with black pigments, the variation in the distribution across the lateral and dorsal sides is discrete and shared among multiple species (Rubtzov 1935; Uvarov 1966). Many species occur both in uniform variants and in variants where dorsal and lateral sides show markedly different colors (Rubtzov 1935; Uvarov 1966). Hence, gomphocerine grasshoppers are often color and pattern polymorphic. A few studies have addressed such pattern polymorphisms in grasshoppers, and they mostly show rather simple genetic inheritance systems with a few interacting loci (Byrne 1967; Dearn 1983; Sansome and La Cour 1935).

**Fig. 1:**
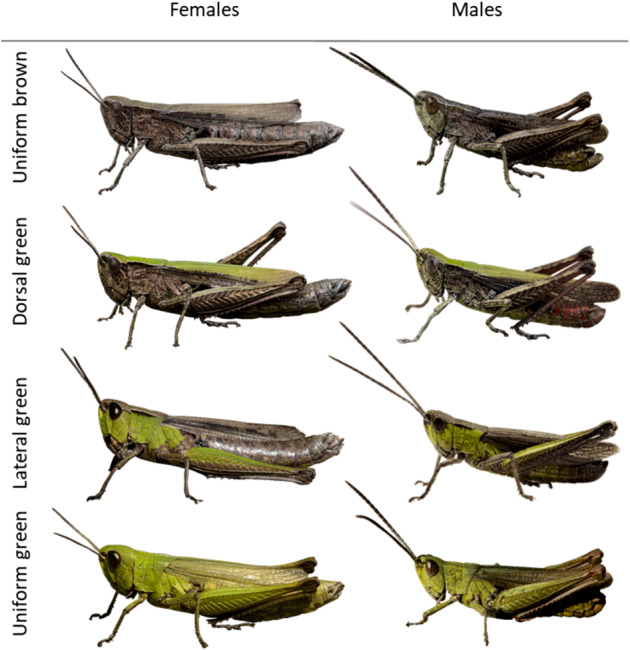
Four distinct color morphs in the steppe grasshopper *Chorthippus dorsatus*. Color morphs differ in the presence and distribution of green areas and occur equally in females and males.

We here present an analysis of the inheritance of color and pattern polymorphism in the steppe grasshopper *Chorthippus dorsatus*, a representative of the gomphocerine subfamily of acridid grasshoppers. The species is widely distributed across Eurasia from Spain to China (Bellmann et al. 2019) and is locally abundant in dry to mesic grasslands. The steppe grasshopper occurs in four distinct color morphs that differ in the distribution of green across the dorsal and lateral sides of the body: uniform brown, dorsal green, lateral green, and uniform green (Fig. 1). The color and pattern polymorphism occurs in both sexes. Not much is known about the spatial distribution of color morphs in their natural habitat, but morphs seem to co-occur over large parts of the range (Rubtzov 1935).

We applied a half-sib-full-sib breeding design to produce offspring from known parental morph combinations and raised the offspring in the laboratory. Such breeding designs allow efficient separation of maternal and genetic effects and are widely used for studying trait inheritance of variable phenotypes (Falconer 1996). In total, we scored more than 1400 offspring from over 130 families raised under two experimentally manipulated rearing environments. This allowed us to test for family effects and environment dependencies of color and pattern polymorphisms. We used simulations to test the fit of different inheritance mechanisms to our observations and found a single parsimonious mechanism that strongly suggests simple genetic inheritance of color morphs.

## Materials and methods

### Founding population

Our breeding design was implemented with field-caught steppe grasshoppers *Chorthippus dorsatus* from Jena, Germany (50.94°N, 11.61°E). A total of 511 individuals (226 males, 285 females) were sampled in June/July 2018 as third or fourth instar nymphae (there are four nymphal stages in this species), ensuring that all individuals were virgin at the time of capture. Subjects were transferred to the laboratory, where they were separated by sex and maintained in groups of up to 90 individuals in cages of dimensions 47.5 × 47.5 × 93 cm^3^. After their final molt, individuals had their color morph scored and were transferred to mating cages of 22 × 16 × 16 cm^3^. Individuals were maintained with ad libitum freshly cut grass potted in small vials filled with water and a water tube for moisture. Small pots containing a 50:50 vermiculite–sand mixture were provided to adult females for egg deposition.

### Scoring of color morphs

Scoring of individuals was straightforward for the distinct morphs: uniform brown (individuals without any green area, abbreviated *B* in the following), dorsal green (individuals that were green dorsally and brown laterally, *D* in the following), lateral green (individuals that were green laterally and brown dorsally, *L* in the following), and uniform green (individuals that showed clear green coloration on both dorsal and lateral sides, *G* in the following, Fig. 1). Coloration is typically most evident on the head and pronotum but extends to the legs, wings, and the anterior part of the abdomen. With a little experience, nymphae are easily scored for the same color variants from at least the third nymphal stage.

### Mating design

**Fig. 2:**
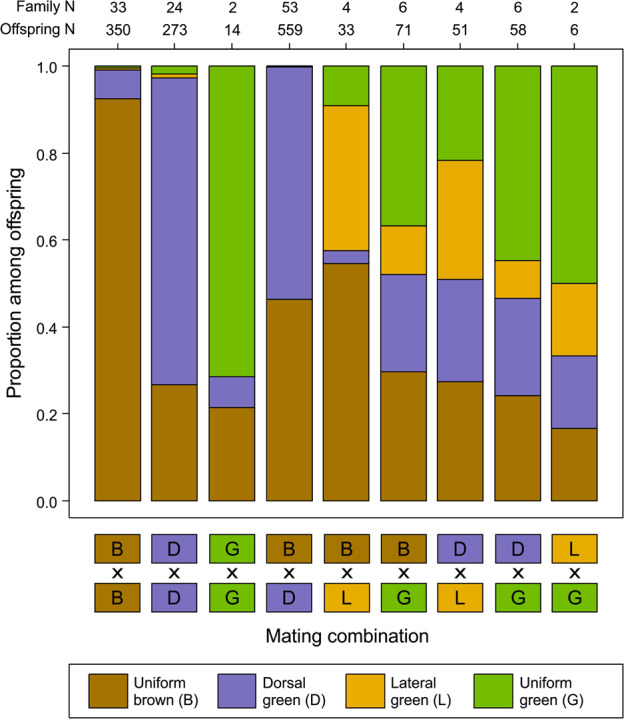
Offspring morph ratios by parental morph mating combination in *Chorthippus dorsatus*. Mating combinations pool both parents of origin. Numbers on the upper margin show sample sizes in terms of number of offspring scored and number of families for each mating combination.

We implemented a half-sib-full-sib breeding design with our field-caught grasshoppers by mating males (*N* = 51) with five females each (*N* = 249 females in total). Each female was kept in a separate mating cage, and males were rotated between “their” five mating cages every third day. We aimed to equalize morph ratios, but since morph ratios were severely skewed in the field, we mated each male to two brown females, two dorsal green females, and either a uniform green female, a lateral green female, or another brown female. The number of successfully reproducing pairs per color morph combination and their progeny is presented in Fig. 2. Although females were provided with sand for egg deposition, most females glued their egg cases to the base of the grass that was provided as food or in the corner of the cage. Egg cases were collected every 7 days and were transferred to Petri dishes lined with moist filter paper, with a single egg case per dish. Petri dishes were regularly moistened and kept at room temperature for several weeks. Eggs were transferred to refrigerators in October, being regularly moistened and kept until spring at about 4 °C. Diapause was ended in seven cohorts between March and September 2019, with one cohort per month. We purposefully equalized the representation of families across cohorts.

### Offspring generation

Offspring hatched after approximately 2 weeks at room temperature. All offspring from a single egg case were transferred to family cages (of dimensions 22 × 16 × 16 cm^3^) and maintained like their parents with ad libitum food and water access. Family size at hatching ranged from 1 to 10 individuals. Offspring molt at intervals of about 1 week until they reach the imaginal stage after about one month. Offspring were scored for color morphs when all individuals of a family had molted into the third nymphal stage (development within families is usually highly synchronized). It is not feasible to follow a large number of subjects individually through development since any external marks would be lost during molt or cause molting problems. Thus, scoring was done by counting the number of individuals of each morph per cage and was done blind to their parents’ color. The first two cohorts were scored independently by GW and HS, but since there was no disagreement between counts, cohorts 3–7 were scored by GW only. All adults were sexed and scored for color morphs again within 1 day after the imaginal molt. Eleven individuals died before their final molt but were still used in the analysis (total *N* = 1415) as the scoring in the third nymphal stage was confirmed for all other individuals (confirmed *N* = 1404).

### Environmental treatment

We assigned full-sib families from the same egg case to one of two treatments. Half of the families were raised in standard plain white housing cages, with food and water provided as described above. The other half of the families were raised in a more complex, enriched environment, in which the floor was lined with patterned green and brown foam rubber, a part of a gray or green egg box for cover and climbing, and a piece of pipe cleaner for climbing. Multiple egg cases from the same mating pair were assigned in equal numbers to both treatments. Treatments were employed in this way for reasons related to the use of data in a separate study. However, the treatment also allowed us to test for possible environment dependencies of the green–brown polymorphism. Effects of crowding on color morph development were assessed via natural variation in rearing group sizes (1–10 individuals per cage).

### Statistical analysis

We used χ^2^ tests to test morph ratios (a) for sex difference, (b) for differences between mating combinations, (c) specifically for differences between reversed parental morph combinations, and (d) for treatment differences within mating combinations. Generalized linear mixed-effects models (GLMMs) with binomial error distribution and logit link controlling for female and male identity gave equivalent results. Generalized linear models with binomial error distribution and logit link were used to estimate the change of morph frequencies with rearing group sizes. All statistical analyses and simulations were performed in R 4.0.2 (R Core Team 2020) using the package lme4 version 1.1–23 for fitting GLMMs (Bates et al. 2015).

### Simulations

We implemented Monte Carlo simulations to test the fit of specific inheritance mechanisms to observed color morph frequencies from different mating combinations. Specifically, we explored the following scenarios (Table 2): (a) one locus with three alleles (two variants), (b) one locus with four alleles, (c) two loci with two alleles each, (d) two loci with two and three alleles each, (e) three loci with two alleles each (four variants), (f) four loci with two alleles each, (g) polygenic threshold model with a single underlying trait, (h) polygenic threshold model with two underlying traits, and (i) polygenic threshold model with three underlying traits (two variants, see Supplementary Material for details). Threshold models are commonly used for analyzing polygenic traits with discrete phenotype expression (Falconer 1996) and were used to evaluate the possibility of polygenic inheritance of color morphs. In all simulations, we proceeded in five steps:

1. Defining dominance: there are many dominance relationships among alternative alleles that can be defined for each scenario. Studies on the green–brown color polymorphism in other Gomphocerine grasshoppers suggest a dominance of green color (see Table 1). However, we also explored other plausible dominance relationships. The Supplementary Material shows just a selection of cases that we have implemented (listed in Table 2).
2. Approximating alleles and genotype frequencies: we chose allele frequencies that fit the field morph frequencies (Table 3) with each specified inheritance mechanism and dominance relationship based on simulation (exploring the full range of allele frequencies). This tuning to field morph ratios ensures that allele frequencies are in a reasonable range for the simulations since any fitting scenario should also be consistent with field morph ratios. Allele frequencies were used to generate expectations for genotype frequencies in the parental generation under the assumption that the population is in Hardy–Weinberg equilibrium.
3. Sampling of potential parents: we sampled potential parents of the appropriate phenotype tightly following our mating design in the number of families of each color morph combination. Sampling was done proportional to expected genotype frequencies after conditioning on parental phenotypes.
4. Simulation of offspring genotypes and phenotypes: we sampled alleles from all parental combinations followed by random mating to generate expectations of offspring genotype frequencies. From these expectations, we sampled the number of offspring that were scored in our data collection. Offspring genotypes were then mapped on phenotypes as defined by a specific scenario to yield simulated offspring numbers for each color morph for all families. Steps (3) and (4) were repeated 1000 times to result in a distribution of morph ratios for each mating combination.
5. Comparison of simulations and observations: to formally compare the fit of the simulation for each scenario to the observations, we calculated the proportion of simulation runs that yielded offspring numbers as extreme as or more extreme (in the sense of difference from the simulated mean) than the observed number of offspring. Small values indicated that the difference is so large that the observed numbers are unlikely to be produced by sampling variation alone under a given simulation scenario. This was done separately by mating combination and offspring morph, yielding a probability value that the observed numbers would be found in the mating design given a specific inheritance mechanism and allele frequencies. A scenario was rejected if it produces significant mismatches for multiple combinations. We considered (and often tried by simulation) if changes to alleles frequencies or dominance relationships would resolve incompatible offspring numbers.

**Table 2:** Overview of inheritance models simulated for the inheritance of color morphs in the steppe grasshopper *Chorthippus dorsatus*.

Inheritance model	No. of genotypes	No. of rules	No. of parameters	Simulated dominance
1 locus, 3 alleles	6	2	2	*D* > *B* > *L*
1 locus, 3 alleles	6	2	2	*D* > *L* > *B*
1 locus, 4 alleles	10	3	3	*D* > *L* > *G* > *B*
2 loci, 2 alleles each	9	2	2	*D* > *u*, *L* > *n*
2 loci, 2 and 3 alleles^a^	18	4	3	*G* > *b*, *D*/*L* > *n*
3 loci, 2 alleles each	27	3	3	*G* > *b*, *U* > *d*, *N* > *l*
3 loci, 2 alleles each^b^	27	3	3	*G* > *b*, *D* > *u*, *L* > *n*
3 loci, 2 alleles each	27	3	3	*G* > *b*, *D* > *u*, *N* > *l*
3 loci, 2 alleles each	27	3	3	*G* > b, *M* > *w*, *R*/*r*
4 loci, 2 alleles each	120	4	4	*G* > *b, D* > *u, L* > *n, M* > *w*
*n* loci, 1 trait	Inf	3	3	*D* > *L* > *G* > *B*
*n* loci, 1 trait	Inf	3	3	*G* > *L* > *D* > *B*
*n* loci, 2 traits	Inf	3	3	*G* > *B*, *L* > *D*
*n* loci, 3 traits	Inf	3	3	*G* > *B*, *G* > *D*, *G* > *L*

The column No. of rules shows the number of dominance relationships (oligogenic models) or order of thresholds (threshold models) to be specified. The column No. of parameters shows the number of allele frequencies (oligogenic models) or thresholds (threshold models) to be estimated. The column Simulated dominance shows the specific dominance relationships. The models are presented in the Supplementary Material.

^a^Second best-fitting model.

^b^Best-fitting model.

**Table 3:** Distribution of color morphs across sexes of the steppe grasshopper *Chorthippus dorsatus*, as sampled from the field for the parental generation and in the laboratory for the offspring generation.

	Number of individuals		Proportion of individuals			χ^2^ test	
	Females	Males	Females	Males	Overall	χ^2^_1_	*P*
*Parental generation*							
Uniform brown	170	127	0.60	0.56	0.58	0.48	0.49
Dorsal green	100	92	0.35	0.41	0.38	1.47	0.23
Lateral green	4	4	0.01	0.02	0.02	0.00	1.00
Uniform green	11	3	0.04	0.01	0.03	2.16	0.14
Total	285	226					
*Offspring generation*							
Uniform brown	355	363	0.53	0.50	0.51	0.84	0.36
Dorsal green	263	291	0.39	0.40	0.40	0.14	0.71
Lateral green	18	26	0.03	0.04	0.03	0.68	0.41
Uniform green	39	46	0.06	0.06	0.06	0.11	0.74
Total	675	726					

χ^2^ test compares the ratios per color morph, given the total number of males and females in each generation.

Simulations are documented in the Supplementary Material, including reasons for rejections of particular scenarios. In the main manuscript, we present what we consider the best-fitting model from our simulations. While more complicated genetic models (including more loci or epistatic interactions) are possible, we consider them less parsimonious to explain our observations.

## Results

We scored color morphs of 1415 offspring from 134 full-sib families (unique female parents) and 46 half-sib families (unique male parents). A total of 727 (52%) offspring were uniform brown, 559 (40%) dorsal green, 44 (3%) lateral green, and 85 (6%) uniform green. Morph frequencies did not differ significantly between the sexes neither in field samples for the parental generation (χ^2^ test: χ^2^_3_ = 6.79, *P* = 0.079) nor in the offspring generation (χ^2^_3_ = 2.16, *P* = 0.14, Table 3).

The distribution of offspring morphs varied significantly across families and was clearly linked to parental phenotypes (χ^2^ test: χ^2^_24_ = 1098.2, *P* < 10^−15^, Fig. 2). Pure matings of uniform brown morphs resulted in 90% uniform brown, 8.4% dorsal green, and one each of lateral green (<1%) and uniform green (<1%) offspring. The occurrences of non-brown offspring from brown–brown matings were clustered in families, with the most extreme being a single large family that produced 14 (50%) uniform brown, 13 (46%) dorsal green, and one (4%) uniform green offspring. Another brown–brown family produced four dorsal green besides two uniform brown offspring.

Pure matings of uniform green morphs were less biased and produced a majority of the parental uniform green morph among their offspring (56%), along with 11% dorsal green and 33% uniform brown offspring (Fig. 2). Similarly, pure matings of dorsal green morphs produced a majority of the parental dorsal green morph (59%), 37% brown offspring, 3.2% uniform green, and 1.6% lateral green offspring (Fig. 2).

**Fig. 3:**
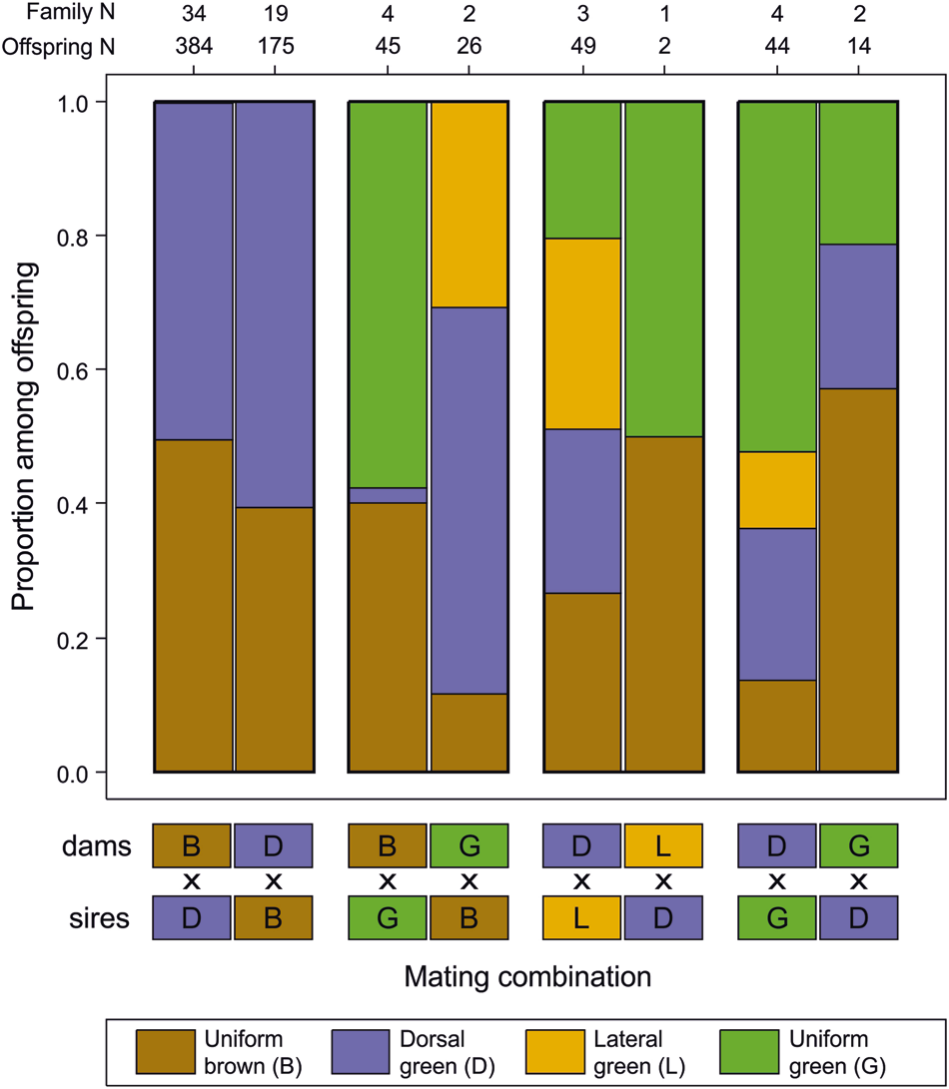
Offspring morph ratios by parental morph mating combination for the four mixed-morph matings for which we had data for both parental combinations in *Chorthippus dorsatus*. Numbers on the upper margin show sample sizes in terms of number of offspring scored and number of families for each mating combination.

Mixed matings produced mixed combinations of offspring, but offspring morph frequencies were markedly different (Fig. 2), and, in particular, lateral green offspring occurred mostly from matings involving a lateral green parent. For four mixed combinations, we had data from both combinations of maternal and paternal color morphs (Fig. 3). Morph frequencies differed significantly in two cases (BxG vs. GxB, χ^2^ test: χ^2^_3_ = 55.88, *P* < 10^−11^ and DxG vs. GxD, χ^2^_3_ = 12.18, *P* = 0.0068). However, the number of families was low, and simulations suggest that the differences might arise from the sampling of genotypes in the parental generations alone, such that they might not represent true parents-of-origin effect.

**Fig. 4:**
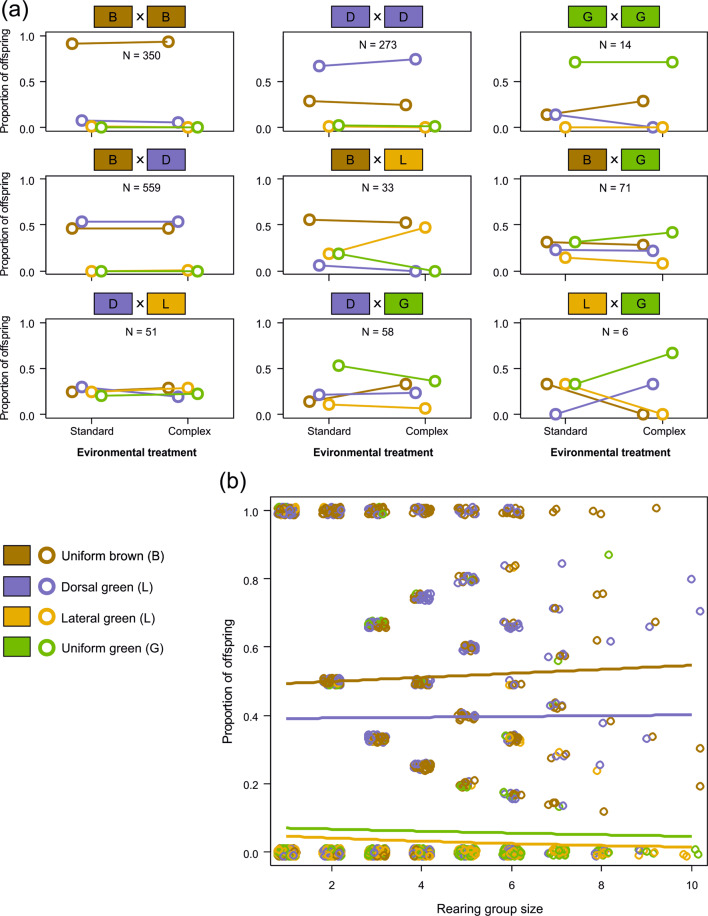
Rearing environment effects on offspring color morphs in *Chorthippus dorsatus*. **a** Effects of habitat complexity on offspring color morphs by mating combination in *Chorthippus dorsatus*. Mating combinations pool both parents of origin. Dots and bars represent the mean proportion of offspring per environmental treatment and standard deviations. **b** Effects of rearing group size on color morphs in *Chorthippus dorsatus*. Rearing groups consisted of full-sib families that varied naturally in the number of hatchlings from a single egg case. Trend lines were fitted from GLMs, and all trends were not significantly different from zero. Data points represent the proportion of a color morph within a rearing group.

Families from the same egg case were raised in either a standard environment or an enriched environment. There was no indication of any rearing-environment effect on offspring color morphs (χ^2^ tests, all *P* > 0.10, Fig. 4a). It has been suggested that crowding favors the development of brown morphs in some grasshopper species. However, there was no indication that morph ratios were affected by rearing group size (GLM slopes, *P* > 0.26 for all color morphs, Fig. 4b).

**Fig. 5:**
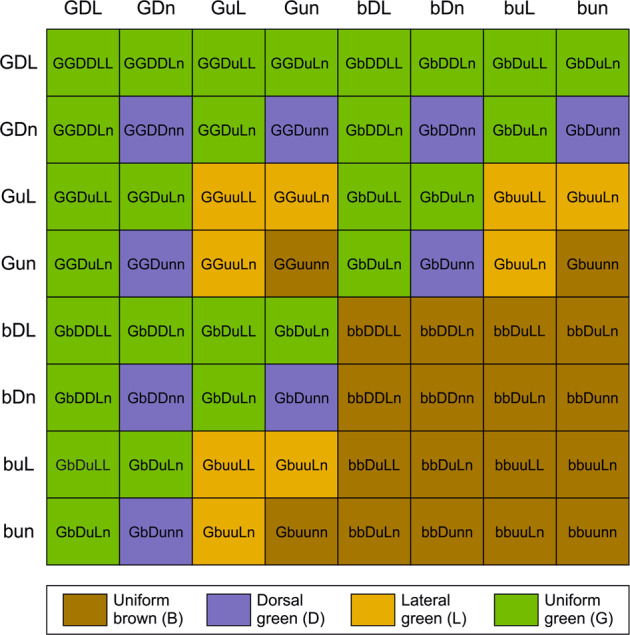
Punnett square illustrating the expected offspring color morph of *Chorthippus dorsatus* for all possible haploid genotypes. Locus *G* controls the ability to produce green (with an allele *G* dominant over *b* to produce green color). Locus *D* controls the color of the dorsal side (producing brown dorsal color when homozygous-recessive *uu*). And locus *L* controls the color of the lateral side (producing brown lateral color when homozygous-recessive *nn*).

We implemented Monte Carlo simulations to test how well various inheritance mechanisms fit with the observations (see Table 2 for an overview and Supplementary Material for details). The most parsimonious mechanism to explain our observations was an autosomal three-locus two-allele inheritance model with (a) one locus *G* controlling the ability to produce green (with an allele *G* dominant over *b* to allow the production of green color), (b) one locus *D* controlling the color of the dorsal side (with a recessive allele *u* that in its homozygote state produces a brown dorsal color independent of other loci, with the alternate dominant allele *D* allowing for green color), and (c) one locus *L* controlling the color of the lateral side (with a recessive allele *n* that in its homozygote state produces a brown lateral color independent of other loci, with the alternate dominant allele *L* allowing for green color). This allows some phenotypically brown individuals to produce non-brown offspring. For example, when phenotypically brown individuals of genotypes *GGuunn* and *bbDunn* are mated, they can produce offspring of genotype *GbDunn* that are phenotypically dorsal green (Fig. 5).

**Fig. 6:**
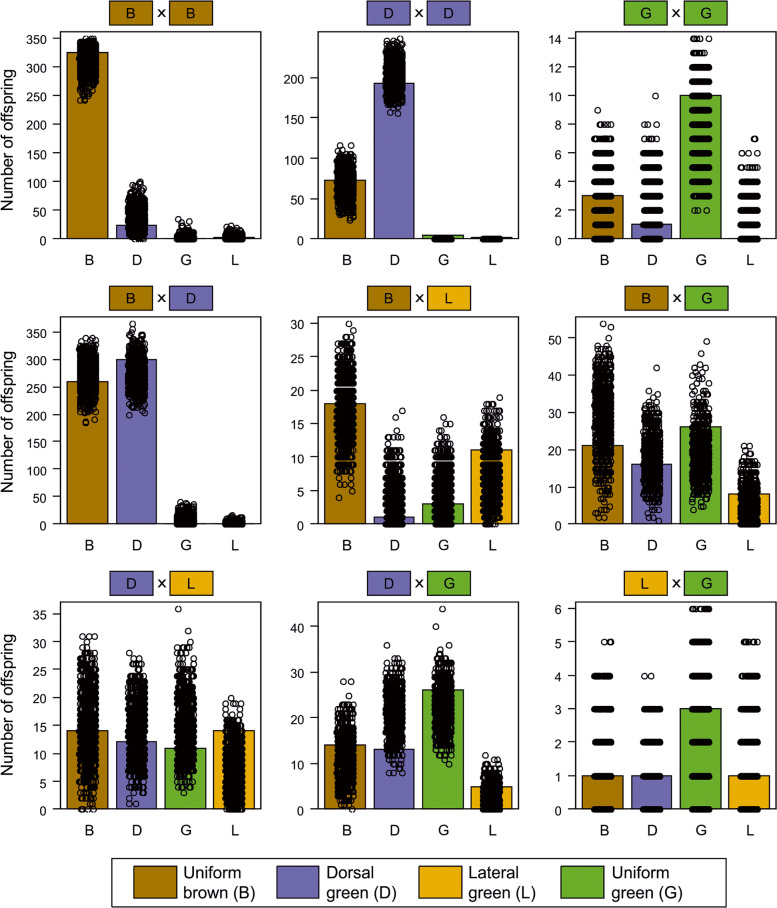
Observed numbers of offspring of each color morphs for different mating combinations (bars) in *Chorthippus dorsatus*, and results from Monte Carlo simulations following the same breeding design (dots). Simulations assume three loci with two alleles each, one controlling the ability to produce any green color, one turning the upper side brown in the homozygous-recessive state, and one turning lateral sides brown in the homozygous-recessive state. Simulations were run with allele frequencies estimated from morph frequencies in the field (*p*_G_ = 0.33, *p*_D_ = 0.49, *p*_L_ = 0.04).

We estimated allele frequencies at these three putative color loci as *p*_G_ = 0.33, *p*_D_ = 0.49, and *p*_L_ = 0.04 based on morph frequencies found among the parental generation in the field. Notably, the three loci were assumed to be autosomally inherited, genetically unlinked, and purely additive in their phenotypic effect. Unlike several alternative inheritance mechanisms (including polygenic inheritance), this simple inheritance mechanism explained observed offspring morph frequencies very well for all mating combinations (Fig. 6, see Model 7 on Supplementary Material). The only exceptions were seven offspring (five uniform green and two lateral green) from dorsal green × dorsal green matings that are not predicted by the three-locus model (2.5% of offspring from the DxD mating combination and 0.5% of all offspring). These offspring might suggest that a second locus is involved in the production of lateral green color (see Model 10, with four loci, on Supplementary Material) or, more generally, incomplete penetrance of the *L* locus.

## Discussion

The results of our breeding experiment are highly indicative of simple genetic inheritance and an absence of detectable habitat complexity and crowding effects on the color and pattern polymorphism in *Chorthippus dorsatus*. Results are most parsimoniously explained by three unlinked autosomal loci, one controlling the ability to produce green and two producing dorsal and lateral brown coloration. Single-locus models and two-locus models cannot explain all offspring morph frequencies for all mating combinations. Similarly, polygenic inheritance does not easily produce the strong patterns among offspring morphs. Even within the three-locus two-allele model, only the scenario of a dominant allele that allows the production of any green and two recessive alleles, each forcing the dorsal or lateral side to turn brown, explains the observations, while other dominance patterns do not.

Although our simulations suggest that the data are compatible with three unlinked autosomal loci, genetic linkage cannot be completely ruled out, in particular since linkage might be incomplete. Linkage to the X chromosomes is unlikely due to the lack of sex differences in morph ratios in both generations and the absence of strong sex-biases within families. Two cases of significant differences in reciprocal crosses might be suggestive of parent-of-original effects (potentially caused by X linkage or cytoplasmic factors). However, simulations show that such differences are likely to appear even with autosomal inheritance, as oligogenic inheritance means that the sampling variation of genotypes is large in parental generations. Selective mortality could, in principle, lead to biased morph ratios, but the survival in the laboratory was high such that post-hatching selection mortality is unlikely to distort the results.

We suggest the following functional hypothesis for the three-locus system. We assume a genetic program regulating the production and deposition of green pigment. The *G* locus might control the formation of the green pigment, and the recessive *b* allele might represent a loss-of-function mutation that disables the production of green pigment. A single functional copy at the *G* locus can thus still produce green pigment. Downstream in the pathways, there is local control about the deposition of green pigment in tissues of the dorsal and lateral sides. Functional copies of *D* and *L* allow the deposition of any green pigment produced (effectively a green-ability variant being dominant and acting locally). However, two non-functional copies *uu* or *nn* (at loci *D* and *L*) prevent the local deposition of green pigment on the dorsal and lateral sides, respectively. In that case, the three loci additively determine color morphs affecting a functional pigmentation pathway, one locus further upstream and two further downstream. Downstream loci affecting color morphs are more likely to be regulatory, while upstream loci might be regulatory or coding variants (Orteau and Jiggins 2020). This hypothesis must be empirically verified by genetic mapping followed by functional analyses.

The results on the inheritance of the green versus brown color variant reported here match with other studies on gomphocerine grasshoppers (Table 1), in which a single major autosomal locus seems to determine the presence of green body color. In three species from the closer sibship of the genus *Chorthippus*, *Chorthippus (Glyptobothrus) brunneus* (Gill 1981), *Gomphocerus sibiricus* (Schielzeth and Dieker 2020), and *Chorthippus dorsatus* (this study), there is strong evidence for the green allele being dominant over the brown allele(s). For the more distantly related *Pseudochorthippus parallelus* (Shah et al. 2020), the mechanism is less clear. Bulk breeding in communal cages resulted in a relatively high proportion of green morphs from brown–brown matings (18%), even higher than the proportion of brown from green–green matings (10%) (Köhler 2006). Even more extreme, there was no brown offspring from green–green matings, but 26% of green offspring from brown–brown matings were reported in Sansome and La Cour (1935). The latter study does not report raw data, the morph classification system is ambiguous, and the study has been criticized before (Richards and Waloff 1954). Nevertheless, both studies on *Pseudochorthippus parallelus* indicate that at least the incidence of green individuals hatching from brown–brown mating is significantly higher than in the other species, suggesting the presence of more genetic or environmental modifiers. It is also possible that the genetic underpinnings are different in *Pseudochorthippus* when compared to the clade comprising *Chorthippus*/*Gomphocerus*.

The most prominent environmental factors that are known to influence the green–brown polymorphism in grasshoppers from the subfamilies Oedipodinae, Cyrtacanthacridinae, and Acridinae are humidity, crowding, and, to a lesser degree, substrate color (Rowell 1970). We did not manipulate humidity and substrate color specifically. Our standard environment consisted mostly of plain white cages (including floor, mesh, and holms), while more complex, enriched environments consisted of patterned background and egg boxes with climbing material. The latter environment was certainly darker overall, with significantly more green areas. From a crypsis point of view, it might seem advantageous to express a mixed body color (potential disruptive coloration) in complex environments (Stevens and Merilaita 2009). However, there was no effect of the rearing environment on offspring color morphs. Furthermore, we tested for effects of natural variation in family size as a measure of crowding but found no effect. Although our conclusions are necessarily limited to the environmental factors that varied during development in the offspring generation, the lack of evidence for environmental influences is in line with previous findings on gomphocerine grasshoppers (Helfert 1978; Valverde and Schielzeth 2015, but see Otte and Williams 1972), but contrasts with findings on species from Oedipodinae, Cyrtacanthacridinae, and Acridinae, where environmental control appears to be common (Dearn 1990; Rowell 1972).

The green–brown polymorphism is thought to be determined by bile pigments synthesized and/or transported in the hemolymph and deposited in the epidermis, producing the green color (Cromartie 1959; Fuzeau-Braesch 1972). The differences between green and brown morphs might consist of the presence of bile pigments per se, but also of the oxidative state of bile pigments that can switch the effect from green to brown coloration (Cromartie 1959). It is possible that the molecular basis of green/brown color differs between color-changing species (e.g., from subfamilies Oedipodinae, Cyrtacanthacridinae, and Acridinae) and species with genetic morph determination in Gomphocerinae. Some of the color-changing species show almost 100% response to the environment (Ergene 1950, 1955; Rowell and Cannis 1971), while in others the response is incomplete (Tanaka 2008). Hormones, juvenile hormones, in particular, might mediate color change as in the polyphenism of solitary/gregarious locusts (Pener 1991; Pener and Simpson 2009; Pener and Yerushalmi 1998).

The most likely ecological driver of morph-specific selection is crypsis (Dearn 1990; Rowell 1972), with perhaps an additional role or trade-offs with thermoregulation (Köhler and Schielzeth 2020). Broad habitat-dependent patterns across the East African (Rowell 1972) and European (Schielzeth 2020) orthopteran fauna suggest that green is favored in vegetated areas, grasslands, bushes, and trees, while ground-dwelling species tend to be brown. This seems very plausible in light of camouflage. Small-scale heterogeneity in selection might contribute to the maintenance of the polymorphism in such habitats (Nilsson and Ripa 2010). There is currently no evidence that the polymorphism affects mate choice or intrasexual competition (limited own data), although this has not been looked for in sufficient detail. Interestingly, the simple genetic inheritance mechanism that we suggest would allow for a substantial diversity of color morphs in offspring (at intermediate allele frequencies), but also the fixation of any locally advantageous color morph.

Overall, in conjunction with previous studies on gomphocerine, our study strongly suggests that the developmental basis of the green–brown polymorphism is very different between species of the subfamily Gomphocerinae (or at least relatives of the genus *Chorthippus*) and other species of Acrididae. The simple genetic inheritance offers potential for genetic mapping in Gomphocerinae. Furthermore, the contrast between color-changing acridids and non-changing gomphocerine grasshoppers offers potential for studying apparent balancing selection under very different conditions in the field. We hope that this study revives an interest in the green–brown polymorphism of orthopterans, so that in further studies we get a clearer picture of how the color morphs are produced developmentally and, ultimately, how such striking phenotypic polymorphism is maintained in natural populations. We think that the green–brown polymorphism of polyneopteran insects may complement studies on other widespread polymorphisms, such as melanism in beetles and lepidopterans (Majerus, 1998).

## Supplementary information

Supplementary Material

## Data Availability

Data available from the Dryad Digital Repository: 10.5061/dryad.0rxwdbs08.
