# Supplementary material for: Simple inheritance of color and pattern polymorphism in the steppe grasshopper Chorthippus dorsatus
Source: Heredity (Edinb). 2021 Apr 16;127(1):66–78. doi: 10.1038/s41437-021-00433-w (PMC8249487; doi:10.1038/s41437-021-00433-w)
Supplement: Supplementary file 1 — Supplementary Material [file 41437_2021_433_MOESM1_ESM.pdf]

Simple inheritance of color and pattern polymorphism in the steppe grasshopper *Chorthippus dorsatus*

Supplementary Material

Gabe Winter, Mahendra Varma, Holger Schielzeth

17/03/2021

Contents

Introduction **2**

R functions for calculations **4**

- Core simulation function for oligogenic models . . . 4
- Core simulation function for polygenic models . . . 5
- Function for estimating allele frequencies for oligogenic models . . . 6
- Function for estimating genotypic thresholds for polygenic models . . . 9
- Function for plotting and summaries . . . 10
- Function for overall model fit evaluation . . . 10
- Function for Punnett square display . . . 11

Overview of models **13**

Single-locus models **14**

- Model 1: Single-locus models with three alleles (D dominant over B, B dominant over L) . . . 14
- Model 2: Single-locus models with three alleles (D dominant over L, L dominant over B) . . . 18
- Model 3: Single-locus models with four alleles . . . 22

Two-locus models **26**

- Model 4: Two-locus models with two alleles . . . 26
- Model 5: Two-locus models with two and three alleles . . . 30

Three-locus models **34**

- Model 6: Three-locus models with two alleles (U and N dominant) . . . 34
- Model 7: Three-locus models with two alleles (D and L dominant) . . . 38
- Model 8: Three-locus models with two alleles (D and N dominant) . . . 42
- Model 9: Three-locus models with two alleles (2 loci for G) . . . 46

Four-locus models **50**

- Model 10: Four-locus models with two alleles (2 dominant alleles for L) . . . 50

Threshold-based multi-locus models **55**

- Model 11: One-trait model with morph genotypic values (D > L > G > B) . . . 55
- Model 12: One-trait model with morph genotypic values (G > L > D > B) . . . 60
- Model 13: Two-trait models . . . 65
- Model 14: Three-trait models . . . 70

Conclusions **75**

Introduction

Our simulations explore different inheritance mechanisms that might explain the results of a half-sib-full-sib breeding design implemented with the steppe grasshopper *Chorthippus dorsatus*. The species occurs in four distinct color morphs: uniform brown (individuals without green areas, B in the following), dorsal green (green dorsally and brown laterally, D), uniform green (clear green coloration on both dorsal and lateral sides, G), and lateral green (green laterally but brown dorsally, L). Morph frequencies at that sampling site are as follows:

```
FieldCounts = c(B=297, D=192, G=14, L=8)
FieldFreq = FieldCounts/sum(FieldCounts)
round(FieldFreq,2)
```

```
##    B    D    G    L
## 0.58 0.38 0.03 0.02
```

We implemented a paternal half-sib-full-sib breeding with a total of 46 different fathers (‘sires’) and 134 mothers (‘dams’) and scored the color morph of 1,415 offspring. We pooled matings in nine distinct mating combinations, irrespective of which phenotype served as a dam or a sire. The following simulations generate the expected offspring morph frequencies given our specific breeding design and compare the simulation results to the empirically determined offspring frequencies.

```
# A path variable needs to be set for the directory
dat = read.table(paste0(path, "Dorsatus_Data_BreedingResults.txt"), header=TRUE)
dat$MatingComb2 = factor(dat$MatingComb2,
        levels=c("B/B", "D/D", "G/G", "B/D", "B/L", "B/G", "D/L", "D/G", "G/L"))
FemaleUq = aggregate(dat$MaleID ~ dat$FemaleID + dat$MorphF, FUN=length)
colnames(FemaleUq) = c("FemaleID", "Morph", "Count")
MaleUq = aggregate(dat$FemaleID ~ dat$MaleID + dat$MorphM, FUN=length)
colnames(MaleUq) = c("MaleID", "Morph", "Count")
```

The observed number of offspring per mating combinations is as follows:

```
obs = data.frame(tapply(dat$nOffsp, list(dat$MatingComb2, dat$MorphOff), sum))
obs[is.na(obs)] = 0
par(mfrow=c(3,3), mar=c(2.5,4,2.5,1))
for(i in 1:9)
  barplot(as.numeric(obs[i,]), names.arg=colnames(obs),
          ylim=c(0,max(obs[i,])), main=rownames(obs)[i], las=1)
```

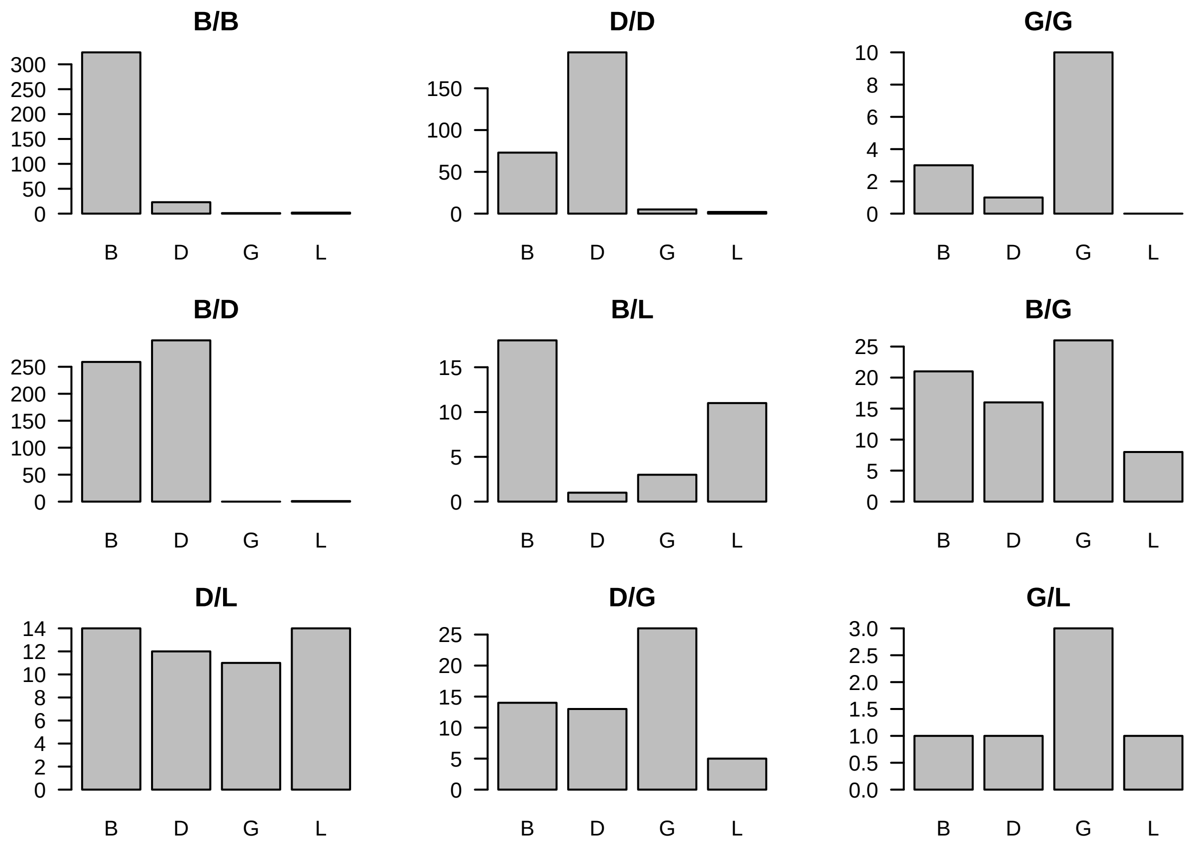
B/B
300
250
200
150
100
50
0
B
D
G
L
D/D
150
100
50
0
B
D
G
L
G/G
10
8
6
4
2
0
B
D
G
L
B/D
250
200
150
100
50
0
B
D
G
L
B/L
15
10
5
0
B
D
G
L
B/G
25
20
15
10
5
0
B
D
G
L
D/L
14
12
10
8
6
4
2
0
B
D
G
L
D/G
25
20
15
10
5
0
B
D
G
L
G/L
3.0
2.5
2.0
1.5
1.0
0.5
0.0
B
D
G
L

R functions for calculations

Core simulation function for oligogenic models

The core simulation function for **oligogenic models** takes a table of genotypes and their frequencies (*gen*), the sampling design (*dat*), and the identities of unique females (*FemaleUq*) and unique males (*MaleUq*) in the mating design to generate a set of potential parental genotypes. For each mating genotype combination, the function calculates expected offspring genotype frequencies, and offspring numbers are drawn from these expectations. The process is repeated *nruns* times, and the results are returned as an array. The option *verbose* allows that the progress is printed on screen.

```
simOligoFnc = function(gen, dat, FemaleUq, MaleUq, nruns, verbose=FALSE) {
  res = array(NA, dim=c(9, 4, nruns))
  # Calculation of expected offspring genotype frequencies
  # for all mating combinations
  Mating = expand.grid(Mother=gen$Genotype, Father=gen$Genotype,
                       FreqOffB=NA, FreqOffD=NA, FreqOffG=NA, FreqOffL=NA)
  Mating = merge(Mating, data.frame(Mother=gen$Genotype, FreqMother=gen$Freq))
  Mating = merge(Mating, data.frame(Father=gen$Genotype, FreqFather=gen$Freq))
  for(i in 1:nrow(Mating)) {
      Locus1 = c(paste0(substr(Mating$Mother[i],1,1), substr(Mating$Father[i],1,1)),
                 paste0(substr(Mating$Mother[i],1,1), substr(Mating$Father[i],2,2)),
                 paste0(substr(Mating$Mother[i],2,2), substr(Mating$Father[i],1,1)),
                 paste0(substr(Mating$Mother[i],2,2), substr(Mating$Father[i],2,2)))
      Locus2 = c(paste0(substr(Mating$Mother[i],3,3), substr(Mating$Father[i],3,3)),
                 paste0(substr(Mating$Mother[i],3,3), substr(Mating$Father[i],4,4)),
                 paste0(substr(Mating$Mother[i],4,4), substr(Mating$Father[i],3,3)),
                 paste0(substr(Mating$Mother[i],4,4), substr(Mating$Father[i],4,4)))
      Locus3 = c(paste0(substr(Mating$Mother[i],5,5), substr(Mating$Father[i],5,5)),
                 paste0(substr(Mating$Mother[i],5,5), substr(Mating$Father[i],6,6)),
                 paste0(substr(Mating$Mother[i],6,6), substr(Mating$Father[i],5,5)),
                 paste0(substr(Mating$Mother[i],6,6), substr(Mating$Father[i],6,6)))
      Locus4 = c(paste0(substr(Mating$Mother[i],7,7), substr(Mating$Father[i],7,7)),
                 paste0(substr(Mating$Mother[i],7,7), substr(Mating$Father[i],8,8)),
                 paste0(substr(Mating$Mother[i],8,8), substr(Mating$Father[i],7,7)),
                 paste0(substr(Mating$Mother[i],8,8), substr(Mating$Father[i],8,8)))
      OffGen = expand.grid(Locus1=Locus1, Locus2=Locus2, Locus3=Locus3, Locus4=Locus4)
      OffGen$Genotype = paste0(OffGen$Locus1, OffGen$Locus2, OffGen$Locus3, OffGen$Locus4)
      OffGen = merge(OffGen, gen[,c("Genotype", "Phenotype")])
      OffGen$Phenotype = factor(OffGen$Phenotype, levels=c("B", "D", "G", "L"))
      Mating[i,c("FreqOffB", "FreqOffD", "FreqOffG", "FreqOffL")] =
        table(OffGen$Phenotype)/sum(table(OffGen$Phenotype))
  }
  if(verbose) print(paste0("Iterations to do: ", nruns))
  for(i in 1:nruns) {
    if(verbose) if(i%%20 ==0) print(paste0("Iterations: ", i))
    # Sampling of parental genotypes conditional on parental phenotypes
      FemaleUq$FemaleGenSim = NA
      MaleUq$MaleGenSim = NA
      for(m in c("B", "D", "G", "L")) {
          gensub = subset(gen, Phenotype==m, select=c("Genotype", "Freq"))
          FemaleUq$FemaleGenSim[FemaleUq$Morph==m] =
            sample(gensub$Genotype, length(FemaleUq$FemaleGenSim[FemaleUq$Morph==m]),
                   replace=TRUE, prob=gensub$Freq)
```

```
        MaleUq$MaleGenSim[MaleUq$Morph==m] =
          sample(gensub$Genotype, length(MaleUq$MaleGenSim[MaleUq$Morph==m]),
                 replace=TRUE, prob=gensub$Freq)
    }
    sim = merge(dat, MaleUq[c("MaleID","MaleGenSim")])
    sim = merge(sim, FemaleUq[c("FemaleID","FemaleGenSim")])
    sim$Mating = paste0(sim$FemaleGenSim, "-", sim$MaleGenSim)
    sim = merge(sim,
                data.frame(Mating=paste0(Mating$Mother,"-", Mating$Father),
                           FeqOffB=Mating$FreqOffB, FeqOffD=Mating$FreqOffD,
                           FeqOffG=Mating$FreqOffG, FeqOffL=Mating$FreqOffL))
    # Simulation of offspring numbers according to the total number
    # of observed offspring per mating. Results of aggregated (by mating
    # combination) simulations are stored in res[,,i]
    sim = cbind(sim, SimOffB=NA, SimOffD=NA, SimOffG=NA, SimOffL=NA)
    for(j in 1:nrow(sim)) {
        samp = sample(c("B", "D", "G", "L"), sim$nOffsp[j], replace=TRUE,
                      prob=sim[j,c("FeqOffB","FeqOffD", "FeqOffG", "FeqOffL")])
        samp = factor(samp, levels=c("B", "D", "G", "L"))
        sim[j,c("SimOffB","SimOffD", "SimOffG", "SimOffL")] = table(samp)
    }
    res[,,i] = as.matrix(c(B=tapply(sim$SimOffB, sim$MatingComb2, sum),
                           D=tapply(sim$SimOffD, sim$MatingComb2, sum),
                           G=tapply(sim$SimOffG, sim$MatingComb2, sum),
                           L=tapply(sim$SimOffL, sim$MatingComb2, sum)),
                         ncol=4, byrow=FALSE)
  }
  return(res)
}
```

Core simulation function for polygenic models

The core simulation function for **polygenic inheritance** takes a table of genotypes for a large number of individuals (*gen*), the threshold values for up to three traits (*ThreshT1*, *ThreshT2*, *ThreshT3*), the sampling design (*dat*), and the identities of unique females (*FemaleUq*) and unique males (*MaleUq*) in the mating design to generate a set of potential parental genotypic values for up to three traits. The function samples potential parental genotypes from the pool of individuals (in *gen*) to generate a set of potential offspring genotypes and, ultimately, a frequency table of offspring phenotypes per mating. This is repeated *nruns* times, and the results are returned as an array. Unlike the *simOligoFnc* of oligogenic models, the *simQuantFnc* does not use a look-up table for assigning phenotypes; hence it also requires a function *phenFnc* to assign phenotypes from genotypic values.

```
simQuantFnc = function(gen, dat, FemaleUq, MaleUq, nruns, phenFnc,
                       ThreshT1, ThreshT2, ThreshT3) {
  res = array(NA, dim=c(9, 4, nruns))
  # Some reformatting to a single row per offspring
  datSimBase = dat[,c("FemaleID", "MaleID", "MatingComb2",
                      "nOffsp", "MorphF", "MorphM")]
  for(i in 1:nrow(datSimBase))
   if(datSimBase$nOffsp[i]>1)
     for(j in 2:datSimBase$nOffsp[i])
      datSimBase = rbind(datSimBase, datSimBase[i,])
  # Beginning of the actual simulation
```

```
  for(n in 1:nruns) {
    FemaleUqSim = cbind(FemaleUq, matrix(NA,ncol=30))
    MaleUqSim   = cbind(MaleUq, matrix(NA,ncol=30))
    for(m in c("B", "D", "G", "L")) {
      gensub = subset(gen, Phenotype==m)
      FemaleUqSim[FemaleUqSim$Morph==m, 4:33] =
        gensub[sample(nrow(gensub), sum(FemaleUqSim$Morph==m), replace=TRUE),1:30]
      MaleUqSim  [  MaleUqSim$Morph==m, 4:33] =
        gensub[sample(nrow(gensub), sum(  MaleUqSim$Morph==m), replace=TRUE),1:30]
    }
    datSim = merge(datSimBase, FemaleUqSim[,c(1,4:33)])
    colnames(datSim)[1:30+6] = paste0("F", 1:30)
    datSim = merge(datSim, MaleUqSim[,c(1,4:33)])
    colnames(datSim)[1:30+36] = paste0("M", 1:30)
    datSim = cbind(datSim, GV1=NA, GV2=NA, GV3=NA)
    # Sampling of alleles from parents. The binomial sampling steps select one or the
    # other parental allele
    for(i in 1:nrow(datSim)) {
        datSim$GV1[i] = mean(as.numeric(datSim[i,c(seq(7,15,by=2),seq(37,45,by=2))
                        + rbinom(30, 1, 0.5)]))
        datSim$GV2[i] = mean(as.numeric(datSim[i,c(seq(17,25,by=2),seq(47,55,by=2))
                        + rbinom(30, 1, 0.5)]))
        datSim$GV3[i] = mean(as.numeric(datSim[i,c(seq(27,35,by=2),seq(57,65,by=2))
                        + rbinom(30, 1, 0.5)]))
    }
    datSim$Phenotype = factor(phenFnc(datSim, ThreshT1, ThreshT2, ThreshT3),
                              levels=c("B", "D", "G", "L"))
    res[,,n] = table(list(datSim$MatingComb2, datSim$Phenotype))
  }
  return(res)
}
```

Function for estimating allele frequencies for oligogenic models

We use simulations to find allele frequencies that fit field morph frequencies. The function simulates all combinations of allele frequencies in steps defined by *AFstep* and calculates the sum of squared deviations from field morph frequencies (*FieldFreq*). Deviations are shown in column *Dev* of the output, with lower values indicating a better fit. A table holding the possible genotypes and their phenotypes (*genSim*) needs to be provided as well as vectors of alleles names at up to four loci (*allelesL1*, *allelesL2*, *allelesL3* and *allelesL4*, respectively). Note that allele names need to be unique across loci. To save computation time, the range of allele frequencies to be explored can be restricted using the *AFmax* argument, a vector (across all alleles at all loci) of maximum allele frequency per allele.

Two additional options (*FirstExchangeble* and *LastExchangeble*) allow constraining the range of allele frequencies in cases where two loci have identical fuctions: The allele frequency of the first allele of the first locus is constraint to be greater or equal to the allele frequency of the first allele in the second locus (for *FirstExchangeble = TRUE*) or, equivalently, the first allele of the second last locus is constraint to be greater or equal to the allele frequency of the first allele in the last locus (for *LastExchangeble = TRUE*). The option *verbose* allows that the progess is printed on screen.

```
AFsimOligoFnc = function(genSim, AFstep=0.02, nLoci=1, FieldFreq,
                         allelesL1 = c("A", "B"), allelesL2=c("C", "D"),
                         allelesL3=c("E", "F"), allelesL4=c("G", "H"), AFmax = NULL,
```

```
                    FirstExchangeble=FALSE, LastExchangeble=FALSE, verbose=FALSE) {
  nAlleles1 = length(allelesL1)
  nAlleles2 = length(allelesL2)
  nAlleles3 = length(allelesL3)
  nAlleles4 = length(allelesL4)
  AFseq = seq(AFstep, 1-AFstep, by=AFstep)
  if(is.null(AFmax)) {
    if(nLoci==1) AFmax = rep(1, nAlleles1)
    if(nLoci==2) AFmax = rep(1, nAlleles1+nAlleles2)
    if(nLoci==3) AFmax = rep(1, nAlleles1+nAlleles2+nAlleles3)
    if(nLoci==4) AFmax = rep(1, nAlleles1+nAlleles2+nAlleles3+nAlleles4)
  }
  AFmax[is.na(AFmax)] = 1
  if(nLoci==1) {
    if(nAlleles1==2) sim = data.frame(expand.grid(AFseq, 0))
    if(nAlleles1==3) sim = data.frame(expand.grid(AFseq, AFseq, 0))
    if(nAlleles1==4) sim = data.frame(expand.grid(AFseq, AFseq, AFseq,  0))
    sim = sim[rowSums(sim[1:nAlleles1]) < 1, ]
    sim[,nAlleles1] = 1 - rowSums(sim[1:nAlleles1])
    colnames(sim) = allelesL1
  }
  if(nLoci==2) {
    if(nAlleles1==2) {
      if(nAlleles2==2) sim = data.frame(expand.grid(AFseq, 0, AFseq, 0))
      if(nAlleles2==3) sim = data.frame(expand.grid(AFseq, 0, AFseq, AFseq, 0))
    }
    if(nAlleles1==3) {
       if(nAlleles2==3) sim = data.frame(expand.grid(AFseq, AFseq, 0, AFseq, AFseq, 0))
    }
    sim = sim[rowSums(sim[1:nAlleles1]) < 1, ]
    sim = sim[rowSums(sim[I(nAlleles1+1):I(nAlleles1+nAlleles2)]) < 1, ]
    sim[,nAlleles1] = 1 - rowSums(sim[1:nAlleles1])
    sim[,nAlleles1+nAlleles2] = 1 - rowSums(sim[I(nAlleles1+1):I(nAlleles1+nAlleles2)])
    colnames(sim) = c(allelesL1, allelesL2)
  }
  if(nLoci==3) {
    if(nAlleles1==2 & nAlleles2==2 & nAlleles3==2) {
      AFseq1 = AFseq[AFseq <= AFmax[1] & AFseq >= 1-AFmax[2]]
      AFseq2 = AFseq[AFseq <= AFmax[3] & AFseq >= 1-AFmax[4]]
      AFseq3 = AFseq[AFseq <= AFmax[5] & AFseq >= 1-AFmax[6]]
      sim = data.frame(expand.grid(AFseq1, 0, AFseq2, 0, AFseq3, 0))
      sim[,seq(2,6,by=2)] = 1 - sim[seq(1,5,by=2)]
      colnames(sim) = c(allelesL1, allelesL2, allelesL3)
      if(LastExchangeble) sim = sim[sim[,3]>=sim[,5],]
      if(FirstExchangeble) sim = sim[sim[,1]>=sim[,3],]
    }
  }
  if(nLoci==4) {
    if(nAlleles1==2 & nAlleles2==2 & nAlleles3==2 & nAlleles4==2) {
      AFseq1 = AFseq[AFseq <= AFmax[1] & AFseq >= 1-AFmax[2]]
      AFseq2 = AFseq[AFseq <= AFmax[3] & AFseq >= 1-AFmax[4]]
      AFseq3 = AFseq[AFseq <= AFmax[5] & AFseq >= 1-AFmax[6]]
      AFseq4 = AFseq[AFseq <= AFmax[7] & AFseq >= 1-AFmax[8]]
```

```
      sim = data.frame(expand.grid(AFseq1, 0, AFseq2, 0, AFseq3, 0, AFseq4, 0))
      sim[,seq(2,8,by=2)] = 1 - sim[seq(1,7,by=2)]
      colnames(sim) = c(allelesL1, allelesL2, allelesL3, allelesL4)
        dim(sim)
      if(LastExchangeble) sim = sim[sim[,5]>=sim[,7],]
      if(FirstExchangeble) sim = sim[sim[,1]>=sim[,3],]
    }
  }
  for(i in 1:length(AFmax))
    if(!is.na(AFmax[i])) sim = sim[sim[,i]<=AFmax[i],]
  sim$PhenB = 0
  sim$PhenD = 0
  sim$PhenG = 0
  sim$PhenL = 0
  if(verbose)
    print(paste0("Iterations to do: ", nrow(sim)))
  for(i in 1:nrow(sim)) {
    if(verbose) if(i %% 100 == 0) print(paste0("Iteration: ", i))
    if(nLoci ==1) {
      AF = sim[i,allelesL1]
      Freq = AF[substr(genSim$Genotype,1,1)] * AF[substr(genSim$Genotype,2,2)]
    }
    if(nLoci ==2) {
      AF = sim[i,c(allelesL1, allelesL2)]
      Freq = AF[substr(genSim$Genotype,1,1)] * AF[substr(genSim$Genotype,2,2)] *
             AF[substr(genSim$Genotype,3,3)] * AF[substr(genSim$Genotype,4,4)]
    }
    if(nLoci ==3) {
      AF = sim[i,c(allelesL1, allelesL2, allelesL3)]
      Freq = AF[substr(genSim$Genotype,1,1)] * AF[substr(genSim$Genotype,2,2)] *
             AF[substr(genSim$Genotype,3,3)] * AF[substr(genSim$Genotype,4,4)] *
             AF[substr(genSim$Genotype,5,5)] * AF[substr(genSim$Genotype,6,6)]
    }
    if(nLoci ==4) {
      AF = sim[i,c(allelesL1, allelesL2, allelesL3, allelesL4)]
      Freq = AF[substr(genSim$Genotype,1,1)] * AF[substr(genSim$Genotype,2,2)] *
             AF[substr(genSim$Genotype,3,3)] * AF[substr(genSim$Genotype,4,4)] *
             AF[substr(genSim$Genotype,5,5)] * AF[substr(genSim$Genotype,6,6)] *
             AF[substr(genSim$Genotype,7,7)] * AF[substr(genSim$Genotype,8,8)]
    }
    genSim$Freq = as.numeric(Freq)
    sim[i,c("PhenB", "PhenD", "PhenG", "PhenL")] =
          tapply(genSim$Freq, genSim$Phenotype, sum)
  }
  sim$Dev = (sim$PhenB - FieldFreq["B"])^2 + (sim$PhenD - FieldFreq["D"])^2
        + (sim$PhenG - FieldFreq["G"])^2 + (sim$PhenL - FieldFreq["L"])^2
  sim[,c("PhenB", "PhenD", "PhenG", "PhenL")] =
      round(sim[,c("PhenB", "PhenD", "PhenG", "PhenL")],2)
  return(sim)
}
```

Function for estimating genotypic thresholds for polygenic models

We use simulations to find genotypic thresholds that fit field morph frequencies. The function simulates all combinations of genotypic thresholds in quantile steps defined by *quantSteps* and calculates the sum of squared deviations from field morph frequencies (*FieldFreq*). Deviations are shown in column *Dev* of the output, with lower values indicating a better fit. A table holding the possible genotypic values (*genSim*) needs to be provided as well as vectors of threshold names for up to three traits (*ThNamesT1*, *ThNamesT2* and *ThNamesT3*, respectively). Note that threshold names should be unique across loci. To save computation time, the range of allele frequencies to be explored can be restricted using the *minT* and *maxT* arguments, vectors (across all thresholds at all traits) of minimum and maximum genotypic values, respectively, for each threshold. The option *verbose* allows that the progess is printed on screen.

```
ThreshSimQuantFnc = function(genSim, quantSteps=0.05, nTraits=1, FieldFreq,
                    ThNamesT1 = c("A", "B"), ThNamesT2=c("C", "D"), ThNamesT3=NA,
                    minT=NA, maxT = NA, phenFnc, verbose=FALSE) {
  TH = qnorm(seq(quantSteps, 1-quantSteps, by=quantSteps))
  if(nTraits ==1) {
    sim = data.frame(expand.grid(TH, TH, TH))
    sim = sim[sim[,1] < sim[,2], ]
    sim = sim[sim[,2] < sim[,3], ]
    colnames(sim) = ThNamesT1
  }
  if(nTraits ==2) {
    sim = data.frame(expand.grid(TH, TH))
    colnames(sim) = c(ThNamesT1, ThNamesT2)
  }
  if(nTraits ==3) {
    sim = data.frame(expand.grid(TH, TH, TH))
    colnames(sim) = c(ThNamesT1, ThNamesT2, ThNamesT3)
  }
  for(i in 1:length(minT))
    if(!is.na(minT[i])) sim = sim[sim[,i]>=minT[i],]
  for(i in 1:length(maxT))
    if(!is.na(maxT[i])) sim = sim[sim[,i]<=maxT[i],]
  sim$PhenB = 0
  sim$PhenD = 0
  sim$PhenG = 0
  sim$PhenL = 0
  if(verbose)
    print(paste0("Iterations to do: ", nrow(sim)))
  for(i in 1:nrow(sim)) {
    if(verbose) if(i %% 100 == 0) print(paste0("Iteration: ", i))
    if(nTraits ==1) {
      ThreshT1 = as.numeric(sim[i,ThNamesT1])
      names(ThreshT1) = ThNamesT1
      Phenotype = phenFnc(genSim, ThreshT1, NA, NA)
    }
    if(nTraits ==2) {
      Phenotype = phenFnc(genSim, sim[i,1], sim[i,2], NA)
    }
    if(nTraits ==3) {
      Phenotype = phenFnc(genSim, sim[i,1], sim[i,2], sim[i,3])
    }
    Phenotype = factor(Phenotype, c("B", "D", "G", "L"))
```

```
    sim[i,c("PhenB", "PhenD", "PhenG", "PhenL")] = table(Phenotype)
  }
  sim$PhenB = sim$PhenB/nrow(genSim)
  sim$PhenD = sim$PhenD/nrow(genSim)
  sim$PhenG = sim$PhenG/nrow(genSim)
  sim$PhenL = sim$PhenL/nrow(genSim)
  sim$Dev = (sim$PhenB - FieldFreq["B"])^2 + (sim$PhenD - FieldFreq["D"])^2
          + (sim$PhenG - FieldFreq["G"])^2 + (sim$PhenL - FieldFreq["L"])^2
  sim[,c("PhenB", "PhenD", "PhenG", "PhenL")] =
      round(sim[,c("PhenB", "PhenD", "PhenG", "PhenL")],2)
  return(sim)
}
```

Function for plotting and summaries

A function for plotting displays the observed offspring phenotype numbers by mating combination (*obs*) as grey bars and the simulation results (*sim*) as dots. The function also calculates summaries for the fit of simulations to observed offspring numbers. First, the proportion of simulations that are as extreme as or more extreme than the observed number of offspring (a p value for the probability that the observations arose by sampling variation given the mating design). Second, *TRUE/FALSE* values for whether the simulations resulted in all-zero values for offspring morphs that were observed in the data. A *TRUE* value might indicate that a particular offspring morph cannot be produced by a particular mating combination (given the inheritance rules being tested). Results are returned as a data frame.

```
plotFnc = function(obs, sim) {
  par(mfrow=c(3,3),mar=c(3,3,3,1))
  for(i in 1:9) {
      barplot(as.numeric(obs[i,]), names.arg=colnames(obs),
              ylim=c(0,max(sim[i,,])), main=rownames(obs)[i], las=1)
      for(j in 1:nruns) points(jitter(1:4),sim[i,,j])
  }
  pVals = cbind(obs, pB=NA, pD=NA, pG=NA, pL=NA)
  pVals = cbind(pVals, BsimO=NA, DsimO=NA, GsimO=NA, LsimO=NA)
  for(i in 1:nrow(obs)){
    for(j in 1:4) {
      pLE = mean(obs[i,j] <= sim[i,j,])
      pME = mean(obs[i,j] >= sim[i,j,])
      pVals[i, j+4] = min(pLE, pME)
      pVals[i, j+8] = all(sim[i,j,]==0) & obs[i,j]>0
    }
  }
  return(pVals)
}
```

Function for overall model fit evaluation

A function for model evaluation that takes the observed offspring phenotype numbers by mating combination (*obs*) and the simulation results (*sim*). It takes the mean across all simulations for all mating combinations and offspring morphs as the prediction given a specific model. It then calculates the (i) sum of absolute deviations for observations (returned as *AbsDev*), (ii) the sum of squared deviations from observations (returned as *SqDev*) and (iii) the square root of the sum of squared deviations from observations as measures of fit (returned as *SrSqDev*). These numbers are compared to a null (random) model that distributes the observed number of

offspring by mating combination equally across all offspring morphs. The ratio of simulated deviations to random deviations give the relative improvement of model predictions compared to a null (random) model. Results for relative comparisions are retured as (*AbsDevRel*, *SqDevRel* and *SrSqDevRel*). Results are returned as a named vector.

```
modevalFnc = function(obs, sim) {
  eval = c(AbsDev=NA, SrSqDev=NA, SqDev=NA,
           AbsDevRel=NA, SrSqDevRel=NA, SqDevRel=NA)
  # Random Model
  nOffsp = rowSums(obs)
  randExp = cbind(nOffsp/4, nOffsp/4, nOffsp/4, nOffsp/4)
  randDevAbs = sum(abs(obs-randExp))
  randDevSq = sum((obs-randExp)^2)
  randDevSrSq = sqrt(sum((obs-randExp)^2))
  # Simulated Model
  modelMean = apply(sim,1:2,median)
  eval["AbsDev"] = sum(abs(obs-modelMean))
  eval["SqDev"] = sum(as.vector(obs-modelMean)^2)
  eval["SrSqDev"] = sqrt(sum(as.vector(obs-modelMean)^2))
  # Relative model fit
  eval["AbsDevRel"] = 1 - eval["AbsDev"] / randDevAbs
  eval["SqDevRel"] = 1 - eval["SqDev"] / randDevSq
  eval["SrSqDevRel"] = 1 - eval["SrSqDev"] / randDevSrSq
  cat("Model fit in offspring numbers and relative improvement to null (uniform) model\n",
      "Absolute deviations:           ",
        round(eval["AbsDev"],0), " (", round(eval["AbsDevRel"]*100,1), "%)\n",
      "Sq root of squared deviations: ",
        round(eval["SrSqDev"],0), " (", round(eval["SrSqDevRel"]*100,1), "%)\n",
      "Sum of squared deviations:     ",
        round(eval["SqDev"],0), " (", round(eval["SqDevRel"]*100,1), "%)\n", sep="")
  return(eval)
}
```

Function for Punnett square display

A convenience function to display the genotype-phenotype table as a Punnett square.

```
PunnettSquare = function(gen, nloci=3) {
  a = substr(gen$Genotype,1,1)
  b = substr(gen$Genotype,2,2)
  if(nloci>=2) a = paste0(a, substr(gen$Genotype,3,3))
  if(nloci>=2) b = paste0(b, substr(gen$Genotype,4,4))
  if(nloci>=3) a = paste0(a, substr(gen$Genotype,5,5))
  if(nloci>=3) b = paste0(b, substr(gen$Genotype,6,6))
  if(nloci==4) a = paste0(a, substr(gen$Genotype,7,7))
  if(nloci==4) b = paste0(b, substr(gen$Genotype,8,8))
  a = unique(a)
  b = unique(b)
  x = data.frame(matrix("B",length(a),length(b)), row.names=unique(a))
  for(i in 1:length(unique(b))) x[,i] = factor(x[,i], levels=c("B", "D", "G", "L"))
  colnames(x) = unique(b)
  for(i in 1:length(a)) {
    for(j in 1:length(b)) {
      genty = paste0(substr(a[i],1,1), substr(b[j],1,1))
```

```
      if(nloci>=2) genty = paste0(genty, substr(a[i],2,2), substr(b[j],2,2))
      if(nloci>=3) genty = paste0(genty, substr(a[i],3,3), substr(b[j],3,3))
      if(nloci==4) genty = paste0(genty, substr(a[i],4,4), substr(b[j],4,4))
      x[i,j] = gen$Phenotype[gen$Genotype==genty]
    }
  }
  return(x)
}
```

Overview of models

We explore a number of inheritance models. Models differ in the number of genotypes they produce and in the number of dominance relationships to be determined. Furthermore, models differ in the number of parameters (allele frequencies and allele threshold) to be approximated and the minimum number of rules (i.e., dominance relationships) to be defined. We consider a parsimonious model one that explains the patterns in the data with the lowest number of parameters, fewest rules, and, preferentially, fewest genotypes. The column labelled *Dominance* shows the cases that are presented in the following section (for details see sections below). The two best-fitting models are Model 7 and Model 5, respectively.

```
##                             Model Genotypes Parameters Rules          Dominance
## Model 1   1 locus, 3 alleles (A)         6          2     2              D>B>L
## Model 2   1 locus, 3 alleles (B)         6          2     2              D>L>B
## Model 3       1 locus, 4 alleles        10          3     3            D>L>G>B
## Model 4         2 loci, 2 alleles         9          2     2           D>u, L>n
## Model 5  2 loci, 2 and 3 alleles        18          3     3         G>b, D=L>n
## Model 6    3 loci, 2 alleles (A)        27          3     3      G>b, U>d, N>l
## Model 7    3 loci, 2 alleles (B)        27          3     3      G>b, D>u, L>n
## Model 8    3 loci, 2 alleles (D)        27          3     3      G>b, D>u, N>l
## Model 9    3 loci, 2 alleles (C)        27          3     3      G>b, M>w, R/r
## Model 10        4 loci, 2 alleles       120          4     4 G>B, D>u, L>n, M>w
## Model 11     n loci, 1 trait (A)       Inf          3     3            D>L>G>B
## Model 12     n loci, 1 trait (B)       Inf          3     3            G>L>D>B
## Model 13         n loci, 2 traits       Inf          2     3           G>B, L>D
## Model 14         n loci, 3 traits       Inf          3     3      G>B, G>D, G>L
```

Single-locus models

Model 1: Single-locus models with three alleles (D dominant over B, B dominant over L)

Three alleles at a single locus might produce a dominance hierarchy. There are at least two rules to be defined: the order of dominance among the three loci. A potential fit to the data are three alleles in dominance sequence $D > B > L$, producing G phenotypes from DL genotypes, D phenotypes from DB or DD genotypes, L phenotypes from LL genotypes, and B phenotypes from BB or BL genotypes. There are $3 * 3 = 9$ possible genotypes (6 of them unique).

```
gen = data.frame(Genotype=rep(NA,9), Phenotype=NA, Freq=NA)
i = 1
for(a1 in c("D", "L", "B"))
  for(a2 in c("D", "L", "B")) {
    gen$Genotype[i] = paste0(a1, a2)
    i = i + 1
  }
gen$Phenotype[is.na(gen$Phenotype) &
              (substr(gen$Genotype,1,1)=="B" & substr(gen$Genotype,2,2)=="B") ] = "B"
gen$Phenotype[is.na(gen$Phenotype) &
              (substr(gen$Genotype,1,1)=="D" | substr(gen$Genotype,2,2)=="D") &
              (substr(gen$Genotype,1,1)=="L" | substr(gen$Genotype,2,2)=="L") ] = "G"
gen$Phenotype[is.na(gen$Phenotype) &
              (substr(gen$Genotype,1,1)=="L" & substr(gen$Genotype,2,2)=="L")] = "L"
gen$Phenotype[is.na(gen$Phenotype) &
              (substr(gen$Genotype,1,1)=="D" | substr(gen$Genotype,2,2)=="D")] = "D"
gen$Phenotype[is.na(gen$Phenotype) &
              (substr(gen$Genotype,1,1)=="B" | substr(gen$Genotype,2,2)=="B")] = "B"
```

These genotypes produce phenotypes as follows.

```
gen[,1:2]
```

```
##   Genotype Phenotype
## 1       DD         D
## 2       DL         G
## 3       DB         D
## 4       LD         G
## 5       LL         L
## 6       LB         B
## 7       BD         D
## 8       BL         B
## 9       BB         B
```

We use simulations to find allele frequencies that fit field morph frequencies. The first round scans in allele frequency steps of 5% to explore the full range of possibilities, the second round scans in steps of 2% and the third in steps of 1% to test for the best combination in a restricted range. The output shows a column *Dev* with the sum of squared deviation between predictions and field morph frequencies, where lower values indicate a better fit (the top 5 results are shown).

```
AFSim = AFsimOligoFnc(gen, AFstep=0.05, nLoci=1, FieldFreq=FieldFreq,
                      allelesL1 = c("D", "L", "B"))
head(AFSim[order(AFSim$Dev),], 5)
```

```
##       D    L    B PhenB PhenD PhenG PhenL          Dev
```

```
## 24 0.25 0.10 0.65  0.55  0.39  0.05  0.01 0.0009628962
## 5  0.25 0.05 0.70  0.56  0.41  0.03  0.00 0.0018017538
## 43 0.25 0.15 0.60  0.54  0.36  0.08  0.02 0.0018736716
## 4  0.20 0.05 0.75  0.64  0.34  0.02  0.00 0.0044451002
## 62 0.25 0.20 0.55  0.52  0.34  0.10  0.04 0.0049090801
```

```
AFSim = AFsimOligoFnc(gen, AFstep=0.02, nLoci=1, FieldFreq=FieldFreq,
                 allelesL1 = c("D", "L", "B"),AFmax=c(0.30, 0.26, 0.8))
head(AFSim[order(AFSim$Dev),], 5)
```

```
##         D    L    B PhenB PhenD PhenG PhenL          Dev
## 159 0.24 0.08 0.68  0.57  0.38  0.04  0.01 0.0001685955
## 208 0.24 0.10 0.66  0.57  0.37  0.05  0.01 0.0001871013
## 110 0.24 0.06 0.70  0.57  0.39  0.03  0.00 0.0003712309
## 257 0.24 0.12 0.64  0.56  0.36  0.06  0.01 0.0004440284
## 60  0.22 0.04 0.74  0.61  0.37  0.02  0.00 0.0006576851
```

```
AFSim = AFsimOligoFnc(gen, AFstep=0.01, nLoci=1, FieldFreq=FieldFreq,
                 allelesL1 = c("D", "L", "B"), AFmax=c(0.30, 0.25, 0.8))
head(AFSim[order(AFSim$Dev),], 5)
```

```
##         D    L    B PhenB PhenD PhenG PhenL          Dev
## 617 0.23 0.07 0.70  0.59  0.37  0.03  0.00 4.675451e-05
## 716 0.23 0.08 0.69  0.59  0.37  0.04  0.01 5.747590e-05
## 518 0.23 0.06 0.71  0.59  0.38  0.03  0.00 7.957845e-05
## 815 0.23 0.09 0.68  0.58  0.37  0.04  0.01 1.135426e-04
## 816 0.24 0.09 0.67  0.57  0.38  0.04  0.01 1.492157e-04
```

```
cat("Combinations within an order of magnitude of best combination: ",
             sum(AFSim$Dev<=min(AFSim$Dev)*10))
```

```
## Combinations within an order of magnitude of best combination:  16
```

A reasonable fit to field morph frequencies can be achieved with allele frequencies of $p_D = 0.23$, $p_L = 0.07$, and $p_B = 0.70$, although this produces too few lateral green (L) individuals.

```
AF = c(D=0.23, L=0.07, B=0.70)
gen$Freq = AF[substr(gen$Genotype,1,1)] * AF[substr(gen$Genotype,2,2)]
if(round(sum(gen$Freq),10)!=1)
  print("Warning: Genotype frequencies do not add to 1")
rbind(Simulated=round(tapply(gen$Freq, gen$Phenotype, sum),2),
      Observed=round(FieldFreq,2))
```

```
##              B    D    G    L
## Simulated 0.59 0.37 0.03 0.00
## Observed  0.58 0.38 0.03 0.02
```

Now parents are sampled from this population according to the implemented mating design. Offspring genotypes are sampled from parental genotypes, offspring phenotypes determined, and the results tabulated. The process is repeated *nruns* times. Simulation results are plotted (dots) along with the observed number of offspring (bars) per mating combination. The following table shows the proportion of simulations that are as extreme or more extreme than the observed number of offspring (a p value for the probability that the observations arose by sampling variation given the mating design) as well as *TRUE*/*FALSE* values for offspring morphs that were observed, but are not predicted. Only mating combinations in which at least one offspring morph shows $p < 0.05$ are shown.

```
nruns=1000
res = simOligoFnc(gen, dat, FemaleUq, MaleUq, nruns)
pVals = plotFnc(obs, res)
```

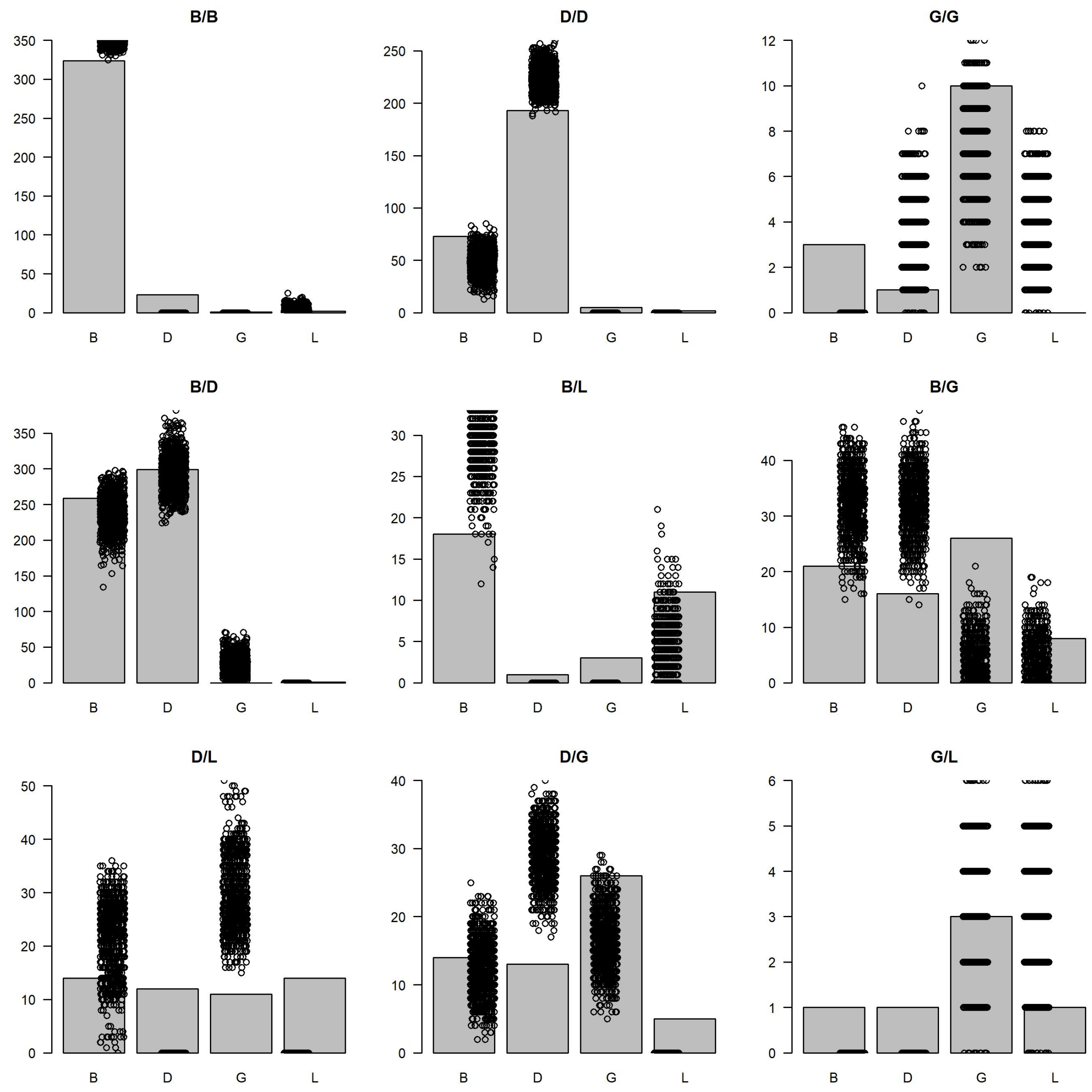


```
evalTab["Model 1", 6:11] = modevalFnc(obs, res)
```

```
## Model fit in offspring numbers and relative improvement to null (uniform) model
## Absolute deviations:             348 (74.8%)
## Sq root of squared deviations: 78 (81.5%)
## Sum of squared deviations:     6074 (96.6%)
```

```
pVals[apply(pVals[,5:8],1,min)<0.05, 5:12]
```

```
##        pB    pD    pG    pL Bsim0 Dsim0 Gsim0 Lsim0
## B/B 0.000 0.000 0.000 0.403 FALSE  TRUE  TRUE FALSE
## D/D 0.017 0.004 0.000 0.000 FALSE FALSE  TRUE  TRUE
## G/G 0.000 0.098 0.090 0.012  TRUE FALSE FALSE FALSE
## B/D 0.231 0.396 0.001 0.000 FALSE FALSE FALSE  TRUE
```

```
## B/L 0.008 0.000 0.000 0.034 FALSE  TRUE  TRUE FALSE
## B/G 0.026 0.002 0.000 0.156 FALSE FALSE FALSE FALSE
## D/L 0.193 0.000 0.000 0.000 FALSE  TRUE FALSE  TRUE
## D/G 0.426 0.000 0.018 0.000 FALSE FALSE FALSE  TRUE
## G/L 0.000 0.000 0.630 0.098  TRUE  TRUE FALSE FALSE
```

The model predicts offspring morph frequencies that are significantly different from observed numbers for all mating combinations. These patterns are thus incompatible with the observed data.

Model 2: Single-locus models with three alleles (D dominant over L, L dominant over B)

The same locus with the same alleles as above might have different dominance relationships with the three alleles in dominance sequence $D > L > B$, producing G phenotypes from DL genotypes, D phenotypes from DB or DD genotypes, L phenotypes from LB and LL genotypes, and B phenotypes from BB genotypes. There are $3 * 3 = 9$ possible genotypes (6 of them unique).

```
gen = data.frame(Genotype=rep(NA,9), Phenotype=NA, Freq=NA)
i = 1
for(a1 in c("D", "L", "B"))
  for(a2 in c("D", "L", "B")) {
    gen$Genotype[i] = paste0(a1, a2)
    i = i + 1
  }
gen$Phenotype[is.na(gen$Phenotype) &
              (substr(gen$Genotype,1,1)=="B" & substr(gen$Genotype,2,2)=="B") ] = "B"
gen$Phenotype[is.na(gen$Phenotype) &
              (substr(gen$Genotype,1,1)=="D" | substr(gen$Genotype,2,2)=="D") &
              (substr(gen$Genotype,1,1)=="L" | substr(gen$Genotype,2,2)=="L") ] = "G"
gen$Phenotype[is.na(gen$Phenotype) &
              (substr(gen$Genotype,1,1)=="L" | substr(gen$Genotype,2,2)=="L")] = "L"
gen$Phenotype[is.na(gen$Phenotype) &
              (substr(gen$Genotype,1,1)=="D" | substr(gen$Genotype,2,2)=="D")] = "D"
```

These genotypes produce phenotypes as follows.

```
gen[,1:2]
```

```
##   Genotype Phenotype
## 1       DD         D
## 2       DL         G
## 3       DB         D
## 4       LD         G
## 5       LL         L
## 6       LB         L
## 7       BD         D
## 8       BL         L
## 9       BB         B
```

We use simulations to find allele frequencies that fit field morph frequencies. The first round scans in allele frequency steps of 5% to explore the full range of possibilities, the second round scans in steps of 2% and the third in steps of 1% to test for the best combination in a restricted range. The output shows a column *Dev* with the sum of squared deviation between predictions and field morph frequencies, where lower values indicate a better fit (the top 5 results are shown).

```
AFSim = AFsimOligoFnc(gen, AFstep=0.05, nLoci=1, FieldFreq=FieldFreq,
                      allelesL1 = c("D", "L", "B"))
head(AFSim[order(AFSim$Dev),], 5)
```

```
##       D    L    B PhenB PhenD PhenG PhenL         Dev
## 4  0.20 0.05 0.75  0.56  0.34  0.02  0.08 0.001627096
## 5  0.25 0.05 0.70  0.49  0.41  0.03  0.07 0.009671617
## 23 0.20 0.10 0.70  0.49  0.32  0.04  0.15 0.011426130
## 3  0.15 0.05 0.80  0.64  0.26  0.02  0.08 0.016277781
## 22 0.15 0.10 0.75  0.56  0.25  0.03  0.16 0.016794109
```

```
AFSim = AFsimOligoFnc(gen, AFstep=0.02, nLoci=1, FieldFreq=FieldFreq,
                      allelesL1 = c("D", "L", "B"), AFmax = c(0.3, 0.18, 0.88))
head(AFSim[order(AFSim$Dev),], 5)
```

```
##       D    L    B PhenB PhenD PhenG PhenL          Dev
## 11 0.22 0.02 0.76  0.58  0.38  0.01  0.03 6.298639e-05
## 59 0.20 0.04 0.76  0.58  0.34  0.02  0.06 1.020094e-03
## 60 0.22 0.04 0.74  0.55  0.37  0.02  0.06 1.132861e-03
## 10 0.20 0.02 0.78  0.61  0.35  0.01  0.03 1.302412e-03
## 12 0.24 0.02 0.74  0.55  0.41  0.01  0.03 2.503754e-03
```

```
AFSim = AFsimOligoFnc(gen, AFstep=0.01, nLoci=1, FieldFreq=FieldFreq,
                      allelesL1 = c("D", "L", "B"), AFmax = c(0.3, 0.15, 0.85))
head(AFSim[order(AFSim$Dev),], 5)
```

```
##        D    L    B PhenB PhenD PhenG PhenL          Dev
## 121 0.22 0.02 0.76  0.58  0.38  0.01  0.03 6.298639e-05
## 219 0.21 0.03 0.76  0.58  0.36  0.01  0.05 1.676567e-04
## 120 0.21 0.02 0.77  0.59  0.37  0.01  0.03 2.043752e-04
## 22  0.22 0.01 0.77  0.59  0.39  0.00  0.02 2.680513e-04
## 220 0.22 0.03 0.75  0.56  0.38  0.01  0.05 3.572962e-04
```

```
cat("Combinations within an order of magnitude of best combination: ",
             sum(AFSim$Dev<=min(AFSim$Dev)*10))
```

```
## Combinations within an order of magnitude of best combination:  6
```

A reasonable fit to field morph frequencies can be achieved with allele frequencies of $p_D = 0.22$, $p_L = 0.02$, and $p_B = 0.76$.

```
AF = c(D=0.22, L=0.02, B=0.76)
gen$Freq = AF[substr(gen$Genotype,1,1)] * AF[substr(gen$Genotype,2,2)]
if(round(sum(gen$Freq),10)!=1)
  print("Warning: Genotype frequencies do not add to 1")
rbind(Simulated=round(tapply(gen$Freq, gen$Phenotype, sum),2),
      Observed=round(FieldFreq,2))
```

```
##              B    D    G    L
## Simulated 0.58 0.38 0.01 0.03
## Observed  0.58 0.38 0.03 0.02
```

Now parents are sampled from this population according to the implemented mating design. Offspring genotypes are sampled from parental genotypes, offspring phenotypes determined, and the results tabulated. The process is repeated *nruns* times. Simulation results are plotted (dots) along with the observed number of offspring (bars) per mating combination. The following table shows the proportion of simulations that are as extreme as or more extreme than the observed number of offspring (a p value for the probability that the observations arose by sampling variation given the mating design). Only mating combinations in which at least one offspring morph shows $p < 0.05$ are shown.

```
nruns=1000
res = simOligoFnc(gen, dat, FemaleUq, MaleUq, nruns)
pVals = plotFnc(obs, res)
```

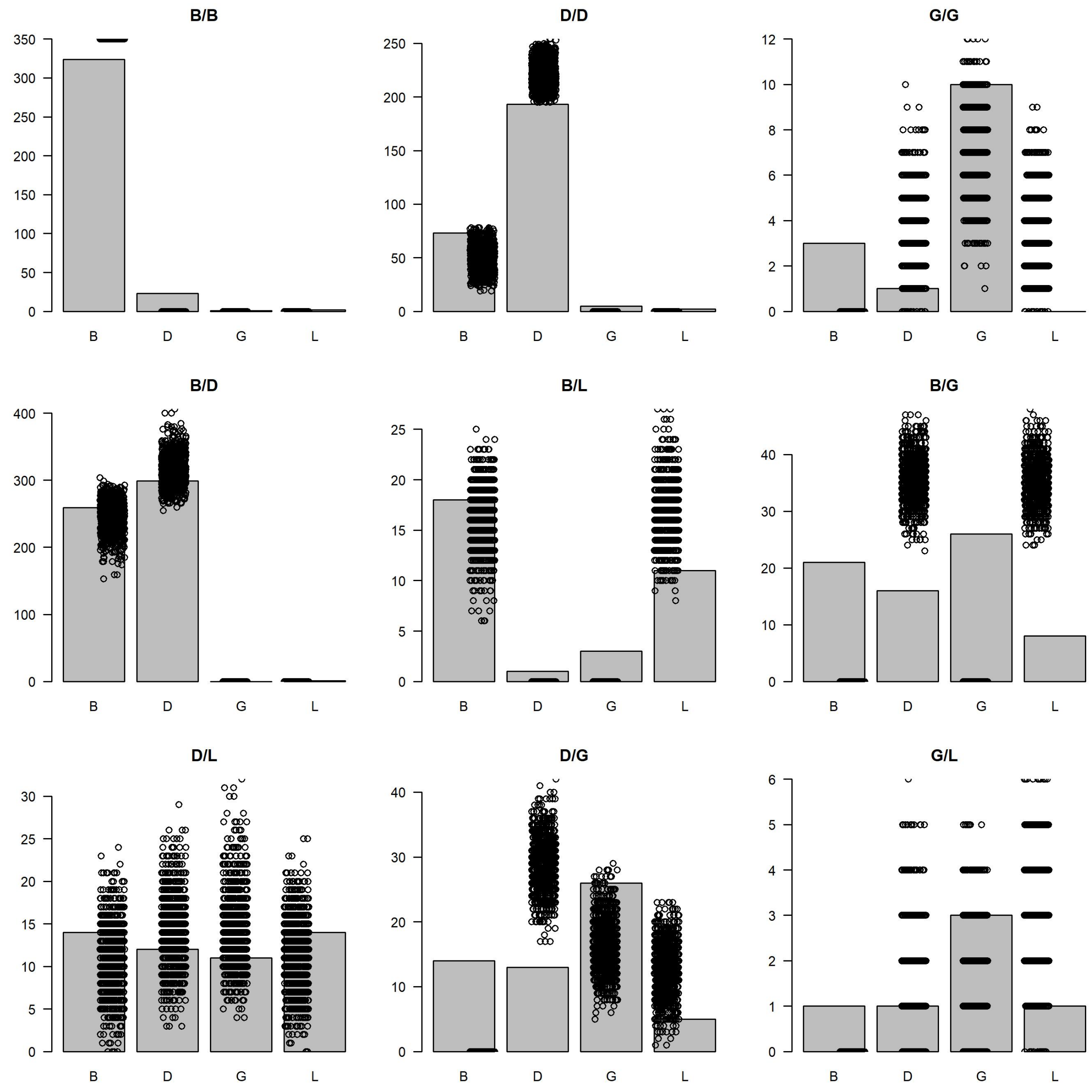


```
evalTab["Model 2", 6:11] = modevalFnc(obs, res)
```

```
## Model fit in offspring numbers and relative improvement to null (uniform) model
## Absolute deviations:            318 (77%)
## Sq root of squared deviations: 76 (82%)
## Sum of squared deviations:     5776 (96.8%)
```

```
pVals[apply(pVals[,5:8],1,min)<0.05, 5:12]
```

```
##        pB    pD    pG    pL Bsim0 Dsim0 Gsim0 Lsim0
## B/B 0.000 0.000 0.000 0.000 FALSE  TRUE  TRUE  TRUE
## D/D 0.033 0.000 0.000 0.000 FALSE FALSE  TRUE  TRUE
## G/G 0.000 0.101 0.085 0.015  TRUE FALSE FALSE FALSE
## B/D 0.291 0.280 1.000 0.000 FALSE FALSE FALSE  TRUE
## B/L 0.323 0.000 0.000 0.031 FALSE  TRUE  TRUE FALSE
```

```
## B/G 0.000 0.000 0.000 0.000  TRUE FALSE  TRUE FALSE
## D/G 0.000 0.000 0.021 0.034  TRUE FALSE FALSE FALSE
## G/L 0.000 0.540 0.165 0.114  TRUE FALSE FALSE FALSE
```

The model predicts offspring morph frequencies that are significantly different from observed numbers for almost all mating combinations. For example for B/B matings, no other offspring morphs than B are possible, for D/D matings L and G offspring morphs are impossible and for G/G matings B offspring morphs are impossible. These patterns are thus incompatible with the observed data.

Model 3: Single-locus models with four alleles

Four alleles at a single locus might produce a dominance hierarchy. There are at least three rules to be defined: the order of dominance among the four loci. A potential fit to the data are four alleles in dominance sequence $D > L > G > B$. There are $4 * 4 = 16$ possible genotypes ($4 + 3 + 2 + 1 = 10$ of them unique).

```
gen = data.frame(Genotype=rep(NA,16), Phenotype=NA, Freq=NA)
i = 1
for(a1 in c("D", "L", "G", "B"))
  for(a2 in c("D", "L", "G", "B")) {
    gen$Genotype[i] = paste0(a1, a2)
    i = i + 1
  }
gen$Phenotype[is.na(gen$Phenotype) &
              (substr(gen$Genotype,1,1)=="D" | substr(gen$Genotype,2,2)=="D")] = "D"
gen$Phenotype[is.na(gen$Phenotype) &
              (substr(gen$Genotype,1,1)=="L" | substr(gen$Genotype,2,2)=="L")] = "L"
gen$Phenotype[is.na(gen$Phenotype) &
              (substr(gen$Genotype,1,1)=="G" | substr(gen$Genotype,2,2)=="G")] = "G"
gen$Phenotype[is.na(gen$Phenotype) &
              (substr(gen$Genotype,1,1)=="B" | substr(gen$Genotype,2,2)=="B")] = "B"
```

These genotypes produce phenotypes as follows.

```
gen[,1:2]
```

```
##    Genotype Phenotype
## 1        DD         D
## 2        DL         D
## 3        DG         D
## 4        DB         D
## 5        LD         D
## 6        LL         L
## 7        LG         L
## 8        LB         L
## 9        GD         D
## 10       GL         L
## 11       GG         G
## 12       GB         G
## 13       BD         D
## 14       BL         L
## 15       BG         G
## 16       BB         B
```

We use simulations to find allele frequencies that fit field morph frequencies. The first round scans in allele frequency steps of 5% to explore the full range of possibilities, the second round scans in steps of 2% and the third in steps of 1% to test for the best combination in a restricted range. The output shows a column *Dev* with the sum of squared deviation between predictions and field morph frequencies, where lower values indicate a better fit (the top 5 results are shown).

```
AFSim = AFsimOligoFnc(gen, AFstep=0.05, nLoci=1, FieldFreq=FieldFreq,
                  allelesL1 = c("D", "L", "G", "B"))
head(AFSim[order(AFSim$Dev),], 5)
```

```
##         D    L    G    B PhenB PhenD PhenG PhenL         Dev
## 4    0.20 0.05 0.05 0.70  0.49  0.36  0.07  0.08 0.008567422
## 3    0.15 0.05 0.05 0.75  0.56  0.28  0.08  0.08 0.010000078
```

```
## 22  0.15 0.10 0.05 0.70  0.49  0.28  0.07  0.16 0.017969758
## 364 0.15 0.05 0.10 0.70  0.49  0.28  0.15  0.08 0.017969758
## 23  0.20 0.10 0.05 0.65  0.42  0.36  0.07  0.15 0.025437468
```

```
AFSim = AFsimOligoFnc(gen, AFstep=0.02, nLoci=1, FieldFreq=FieldFreq,
                 allelesL1 = c("D", "L", "G", "B"),
                 AFmax=c(0.28, 0.18, 0.18, 0.8))
head(AFSim[order(AFSim$Dev),], 5)
```

```
##         D    L    G    B PhenB PhenD PhenG PhenL          Dev
## 10   0.20 0.02 0.02 0.76  0.58  0.36  0.03  0.03 0.0002606102
## 59   0.20 0.04 0.02 0.74  0.55  0.36  0.03  0.06 0.0013774086
## 2411 0.20 0.02 0.04 0.74  0.55  0.36  0.06  0.03 0.0013774086
## 11   0.22 0.02 0.02 0.74  0.55  0.39  0.03  0.03 0.0013815890
## 58   0.18 0.04 0.02 0.76  0.58  0.33  0.03  0.06 0.0023299240
```

```
AFSim = AFsimOligoFnc(gen, AFstep=0.01, nLoci=1, FieldFreq=FieldFreq,
                 allelesL1 = c("D", "L", "G", "B"),
                 AFmax=c(0.25, 0.15, 0.15, 0.8))
head(AFSim[order(AFSim$Dev),], 5)
```

```
##         D    L    G    B PhenB PhenD PhenG PhenL          Dev
## 120  0.21 0.02 0.01 0.76  0.58  0.38  0.02  0.03 1.308359e-05
## 9822 0.21 0.01 0.02 0.76  0.58  0.38  0.03  0.02 1.308359e-05
## 21   0.21 0.01 0.01 0.77  0.59  0.38  0.02  0.02 1.366064e-04
## 218  0.20 0.03 0.01 0.76  0.58  0.36  0.02  0.05 2.606102e-04
## 9920 0.20 0.02 0.02 0.76  0.58  0.36  0.03  0.03 2.606102e-04
```

```
cat("Combinations within an order of magnitude of best combination: ",
             sum(AFSim$Dev<=min(AFSim$Dev)*10))
```

```
## Combinations within an order of magnitude of best combination:  2
```

A good fit to field morph frequencies can be achieved with allele frequencies of $p_D = 0.21$, $p_L = 0.02$, $p_G = 0.01$ and $p_B = 0.76$.

```
AF = c(D=0.21, L=0.02, G=0.01, B=0.76)
gen$Freq = AF[substr(gen$Genotype,1,1)] * AF[substr(gen$Genotype,2,2)]
if(round(sum(gen$Freq),10)!=1)
  print("Warning: Genotype frequencies do not add to 1")
rbind(Simulated=round(tapply(gen$Freq, gen$Phenotype, sum),2),
      Observed=round(FieldFreq,2))
```

```
##              B    D    G    L
## Simulated 0.58 0.38 0.02 0.03
## Observed  0.58 0.38 0.03 0.02
```

Now parents are sampled from this population according to the implemented mating design. Offspring genotypes are sampled from parental genotypes, offspring phenotypes determined, and the results tabulated. The process is repeated *nruns* times. Simulation results are plotted (dots) along with the observed number of offspring (bars) per mating combination. The following table shows the proportion of simulations that are as extreme or more extreme than the observed number of offspring (a p value for the probability that the observations arose by sampling variation given the mating design). Only mating combinations in which at least one offspring morph shows $p < 0.05$ are shown.

```
nruns=1000
res = simOligoFnc(gen, dat, FemaleUq, MaleUq, nruns)
pVals = plotFnc(obs, res)
```

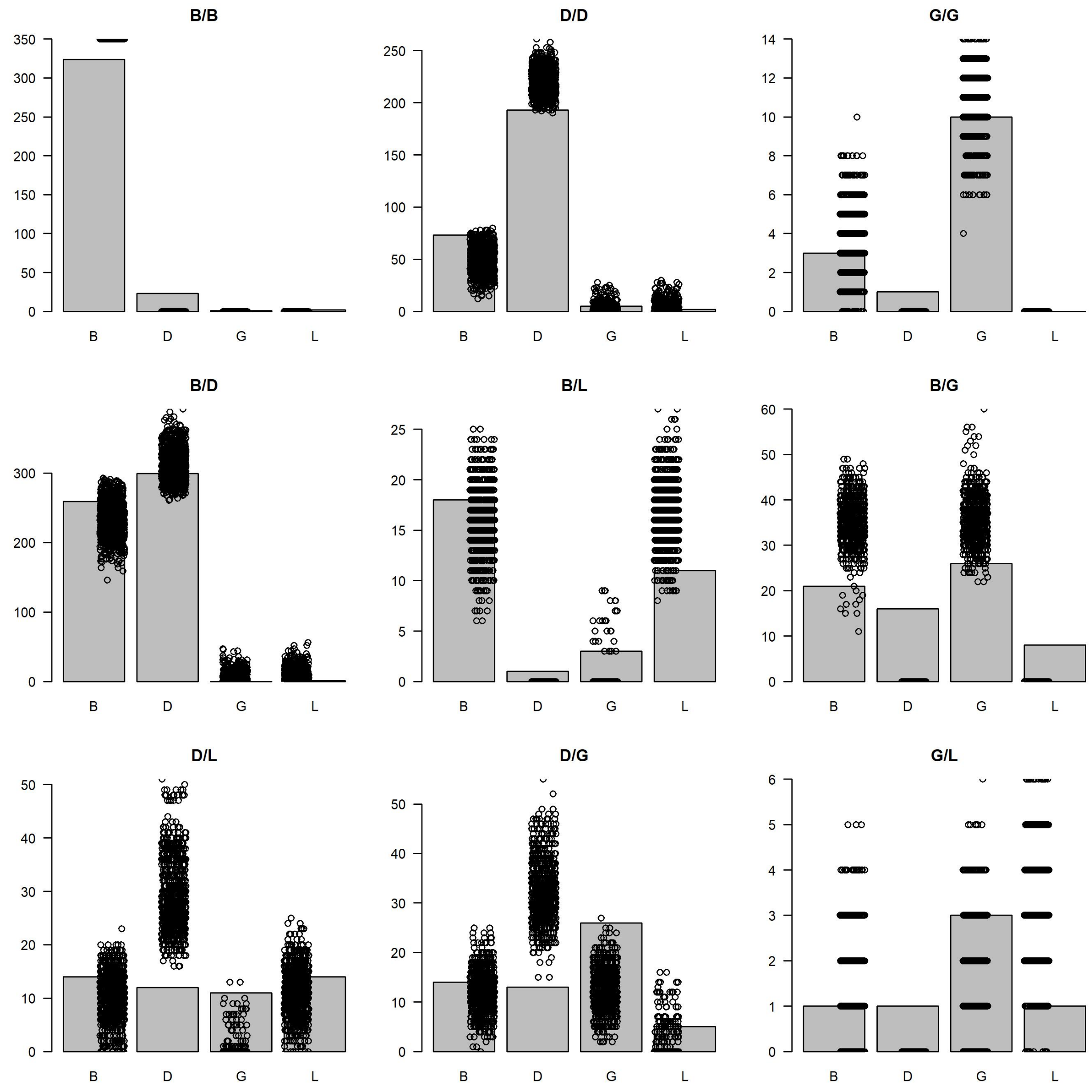


```
evalTab["Model 3", 6:11] = modevalFnc(obs, res)
```

```
## Model fit in offspring numbers and relative improvement to null (uniform) model
## Absolute deviations:          277 (79.9%)
## Sq root of squared deviations: 67 (84%)
## Sum of squared deviations:     4537 (97.4%)
```

```
pVals[apply(pVals[,5:8],1,min)<0.05, 5:12]
```

```
##        pB    pD    pG    pL Bsim0 Dsim0 Gsim0 Lsim0
## B/B 0.000 0.000 0.000 0.000 FALSE  TRUE  TRUE  TRUE
## D/D 0.023 0.005 0.110 0.342 FALSE FALSE FALSE FALSE
## G/G 0.538 0.000 0.462 1.000 FALSE  TRUE FALSE FALSE
## B/L 0.344 0.000 0.027 0.039 FALSE  TRUE FALSE FALSE
## B/G 0.011 0.000 0.026 0.000 FALSE  TRUE FALSE  TRUE
```

```
## D/L 0.248 0.000 0.002 0.317 FALSE FALSE FALSE FALSE
## D/G 0.378 0.000 0.001 0.073 FALSE FALSE FALSE FALSE
## G/L 0.569 0.000 0.181 0.116 FALSE  TRUE FALSE FALSE
```

The model predicts offspring morph frequencies that are significantly different from observed numbers for most mating combinations. For example for B/B matings, no other offspring morphs than B are possible, and from G/G, B/G and G/L matings, D offspring will not be produced at all. These patterns are thus incompatible with the observed data. More generally, changes in the dominance order will produce one phenotype (here B) that is only produced by a homozygous recessive genotype, and pure matings involving this genotype (here B/B) will only produce offspring of a single morph. There is no such mating combination in our dataset to produces exclusively one offspring morph. Alternative dominance rankings are thus also incompatible with the observed data.

Two-locus models

Model 4: Two-locus models with two alleles

There might be two loci involved in color morph determination: one locus D controlling green on the dorsal side, and one locus L controlling green on the lateral side. There are at least two rules to be defined: the order of dominance at two loci. A potentially fitting mechanism is the dominance of a green allele at both loci (D and L, respectively), and a recessive allele (u and n, respectively) that produces brown color in its homozygous state. There are $(2 * 2)^2 = 16$ possible genotypes ($3 * 3 = 9$ of them unique).

```
gen = data.frame(Genotype=rep(NA,16), Phenotype=NA, Freq=NA)
i = 1
for(d1 in c("D", "u"))
  for(d2 in c("D", "u"))
    for(l1 in c("L", "n"))
      for(l2 in c("L", "n")) {
        gen$Genotype[i] = paste0(d1, d2, l1, l2)
        i = i + 1
      }
gen$Phenotype = NA
gen$Phenotype[is.na(gen$Phenotype) &
              (substr(gen$Genotype,1,1)=="u" & substr(gen$Genotype,2,2)=="u") &
              (substr(gen$Genotype,3,3)=="n" & substr(gen$Genotype,4,4)=="n")] = "B"
gen$Phenotype[is.na(gen$Phenotype) &
              (substr(gen$Genotype,1,1)=="u" & substr(gen$Genotype,2,2)=="u") &
              (substr(gen$Genotype,3,3)=="L" | substr(gen$Genotype,4,4)=="L")] = "L"
gen$Phenotype[is.na(gen$Phenotype) &
              (substr(gen$Genotype,1,1)=="D" | substr(gen$Genotype,2,2)=="D") &
              (substr(gen$Genotype,3,3)=="n" & substr(gen$Genotype,4,4)=="n")] = "D"
gen$Phenotype[is.na(gen$Phenotype) &
              (substr(gen$Genotype,1,1)=="D" | substr(gen$Genotype,2,2)=="D") &
              (substr(gen$Genotype,3,3)=="L" | substr(gen$Genotype,4,4)=="L")] = "G"
```

These genotypes produce phenotypes as follows.

```
PunnettSquare(gen, nloci=2)
```

```
##    DL Dn uL un
## DL  G  G  G  G
## Dn  G  D  G  D
## uL  G  G  L  L
## un  G  D  L  B
```

We use simulations to find allele frequencies that fit field morph frequencies. The first round scans in allele frequency steps of 5% to explore the full range of possibilities, the second round scans in steps of 2% and the third in steps of 1% to test for the best combination in a restricted range. The output shows a column *Dev* with the sum of squared deviation between predictions and field morph frequencies, where lower values indicate a better fit (the top 5 results are shown).

```
AFSim = AFsimOligoFnc(gen, AFstep=0.05, nLoci=2, FieldFreq=FieldFreq,
                      allelesL1 = c("D", "u"), allelesL2 = c("L", "n"))
head(AFSim[order(AFSim$Dev),], 5)
```

```
##        D    u    L    n PhenB PhenD PhenG PhenL         Dev
## 4   0.20 0.80 0.05 0.95  0.58  0.32  0.04  0.06 0.002597137
## 5   0.25 0.75 0.05 0.95  0.51  0.39  0.04  0.05 0.005775829
```

```
## 23 0.20 0.80 0.10 0.90  0.52  0.29  0.07  0.12 0.011024017
## 24 0.25 0.75 0.10 0.90  0.46  0.35  0.08  0.11 0.016228624
## 3  0.15 0.85 0.05 0.95  0.65  0.25  0.03  0.07 0.020716333
```

```
AFSim = AFsimOligoFnc(gen, AFstep=0.02, nLoci=2, FieldFreq=FieldFreq,
                  allelesL1 = c("D", "u"), allelesL2 = c("L", "n"),
                  AFmax = c(0.3, 0.9, 0.18, 1.0))
head(AFSim[order(AFSim$Dev),], 5)
```

```
##       D    u    L    n PhenB PhenD PhenG PhenL          Dev
## 11 0.22 0.78 0.02 0.98  0.58  0.38  0.02  0.02 9.701889e-06
## 60 0.22 0.78 0.04 0.96  0.56  0.36  0.03  0.05 6.408227e-04
## 12 0.24 0.76 0.02 0.98  0.55  0.41  0.02  0.02 1.597872e-03
## 59 0.20 0.80 0.04 0.96  0.59  0.33  0.03  0.05 2.006437e-03
## 10 0.20 0.80 0.02 0.98  0.61  0.35  0.01  0.03 2.017805e-03
```

```
AFSim = AFsimOligoFnc(gen, AFstep=0.01, nLoci=2, FieldFreq=FieldFreq,
                  allelesL1 = c("D", "u"), allelesL2 = c("L", "n"),
                  AFmax = c(0.3, 0.9, 0.15, 1.0))
head(AFSim[order(AFSim$Dev),], 5)
```

```
##        D    u    L    n PhenB PhenD PhenG PhenL          Dev
## 121 0.22 0.78 0.02 0.98  0.58  0.38  0.02  0.02 9.701889e-06
## 220 0.22 0.78 0.03 0.97  0.57  0.37  0.02  0.04 1.298692e-04
## 22  0.22 0.78 0.01 0.99  0.60  0.38  0.01  0.01 2.925706e-04
## 122 0.23 0.77 0.02 0.98  0.57  0.39  0.02  0.02 3.714643e-04
## 219 0.21 0.79 0.03 0.97  0.59  0.35  0.02  0.04 5.222110e-04
```

```
cat("Combinations within an order of magnitude of best combination: ",
             sum(AFSim$Dev<=min(AFSim$Dev)*10))
```

```
## Combinations within an order of magnitude of best combination:  1
```

A reasonable fit to field morph frequencies can be achieved with allele frequencies of $p_D = 0.22$, $p_u = 0.78$, $p_L = 0.02$ and $, $p_n = 0.98$.

```
gen$Freq = NA
AF = c(D=0.22, u=0.78, L=0.02, n=0.98)
gen$Freq = AF[substr(gen$Genotype,1,1)] * AF[substr(gen$Genotype,2,2)] *
           AF[substr(gen$Genotype,3,3)] * AF[substr(gen$Genotype,4,4)]
if(round(sum(gen$Freq),10)!=1)
  print("Warning: Genotype frequencies do not add to 1")
rbind(Simulated=round(tapply(gen$Freq, gen$Phenotype, sum),2),
      Observed=round(FieldFreq,2))
```

```
##              B    D    G    L
## Simulated 0.58 0.38 0.02 0.02
## Observed  0.58 0.38 0.03 0.02
```

Now parents are sampled from this population according to the implemented mating design. Offspring genotypes are sampled from parental genotypes, offspring phenotypes determined, and the results tabulated. The process is repeated *nruns* times. Simulation results are plotted (dots) along with the observed number of offspring (bars) per mating combination. The following table shows the proportion of simulations that are as extreme as or more extreme than the observed number of offspring (a p value for the probability that the observations arose by sampling variation given the mating design). Only mating combinations in which at least one offspring morph shows $p < 0.05$ are shown.

```
nruns=1000
res = simOligoFnc(gen, dat, FemaleUq, MaleUq, nruns)
pVals = plotFnc(obs, res)
```

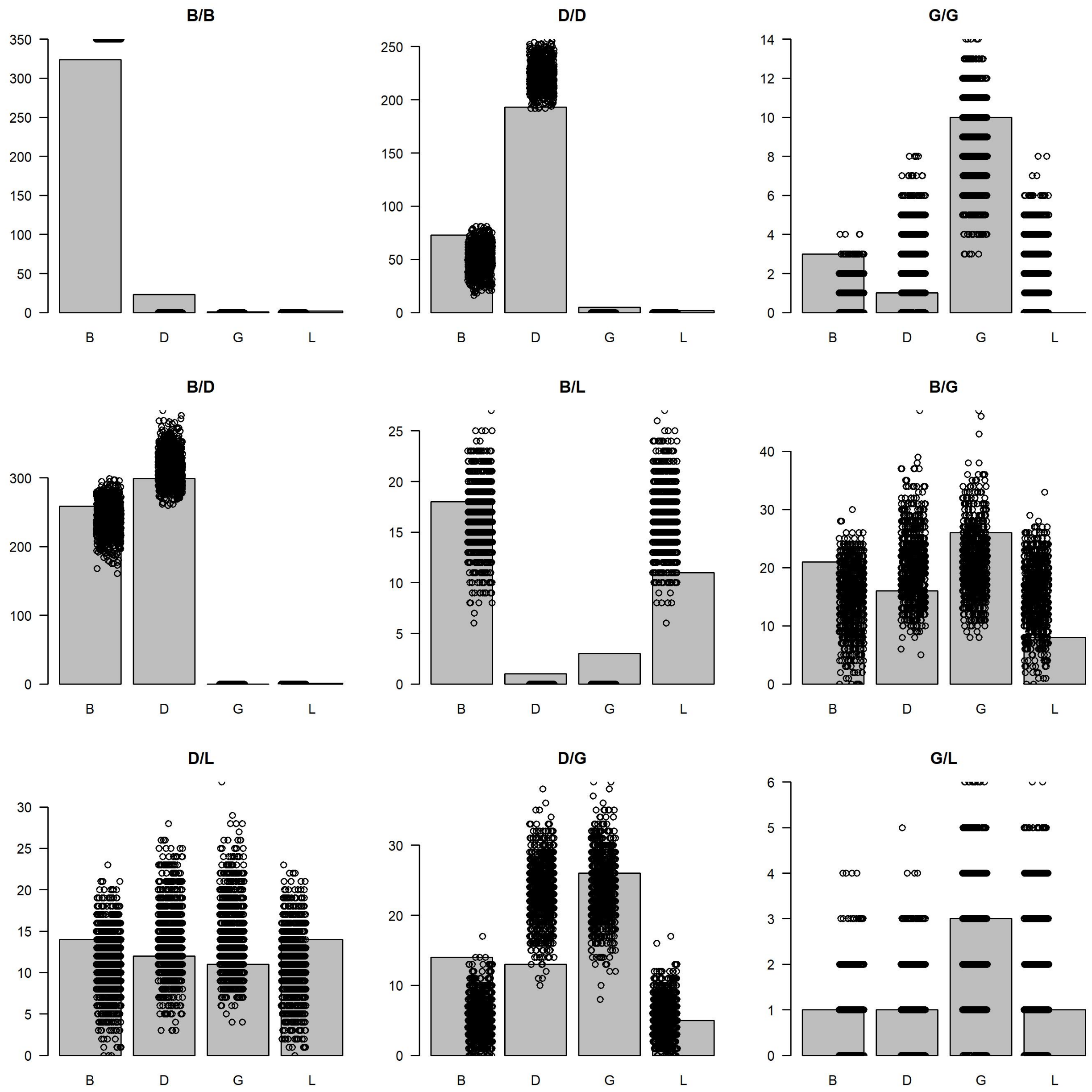


```
evalTab["Model 4", 6:11] = modevalFnc(obs, res)
```

```
## Model fit in offspring numbers and relative improvement to null (uniform) model
## Absolute deviations:           206 (85.1%)
## Sq root of squared deviations: 55 (87%)
## Sum of squared deviations:     3014 (98.3%)
```

```
pVals[apply(pVals[,5:8],1,min)<0.05, 5:12]
```

```
##        pB    pD    pG    pL Bsim0 Dsim0 Gsim0 Lsim0
## B/B 0.000 0.000 0.000 0.000 FALSE  TRUE  TRUE  TRUE
```

```
## D/D 0.027 0.005 0.000 0.000 FALSE FALSE  TRUE  TRUE
## G/G 0.042 0.258 0.354 0.251 FALSE FALSE FALSE FALSE
## B/D 0.297 0.281 1.000 0.000 FALSE FALSE FALSE  TRUE
## B/L 0.366 0.000 0.000 0.054 FALSE  TRUE  TRUE FALSE
## D/G 0.004 0.007 0.344 0.502 FALSE FALSE FALSE FALSE
```

The model predicts offspring morph frequencies that are significantly different from observed numbers for several mating combinations. For example for B/B matings, no other offspring morphs than B are possible, for D/D matings G and L offspring are impossible and for B/L matings D and G offspring are impossible. These patterns are thus incompatible with the observed data. More generally, changes in the dominance order will produce one phenotype (here B) that is only produced by a double homozygous recessive genotype, and pure matings involving this genotype (here B/B) will only produce offspring of a single morph. There is no such mating combination in our dataset to produces exclusively one offspring morph. Alternative dominance rankings are thus also incompatible with the observed data.

Model 5: Two-locus models with two and three alleles

There might be two loci involved in color morph determination: one locus G controlling ability to produce green and one locus R that controls the regions in which green occurs. There are $3 * 6 = 18$ unique genotypes and at least two rules to be defined: the order of dominance at two loci. A potentially fitting mechanisms has two alleles, G and b, at a G locus where the ability to produce green (G) is dominant over brown (b). For the region that controls green, there could be three alleles, D for dorsal green, L for lateral green and n for no green, with D and L being dominant over n, and D and L co-dominant, such that if the ability to produce green is given (by the G locus), the DL genotype produces fully green, the Dn genotype dorsal green, the Ln genotype lateral green and the nn genotype brown.

```
gen = data.frame(Genotype=rep(NA,36), Phenotype=NA, Freq=NA)
i = 1
for(d1 in c("G", "b"))
  for(d2 in c("G", "b"))
    for(l1 in c("D", "L", "n"))
      for(l2 in c("D", "L", "n")) {
        gen$Genotype[i] = paste0(d1, d2, l1, l2)
        i = i + 1
      }
gen$Phenotype = NA
gen$Phenotype[is.na(gen$Phenotype) &
              (substr(gen$Genotype,1,1)=="b" & substr(gen$Genotype,2,2)=="b") |
              (substr(gen$Genotype,3,3)=="n" & substr(gen$Genotype,4,4)=="n")] = "B"
gen$Phenotype[is.na(gen$Phenotype) &
              (substr(gen$Genotype,3,3)=="L" & substr(gen$Genotype,4,4)=="D") |
              (substr(gen$Genotype,3,3)=="D" & substr(gen$Genotype,4,4)=="L")] = "G"
gen$Phenotype[is.na(gen$Phenotype) &
              (substr(gen$Genotype,3,3)=="D" | substr(gen$Genotype,4,4)=="D")] = "D"
gen$Phenotype[is.na(gen$Phenotype) &
              (substr(gen$Genotype,3,3)=="L" | substr(gen$Genotype,4,4)=="L")] = "L"
```

These genotypes produce phenotypes as follows.

```
PunnettSquare(gen, nloci=2)
```

```
##    GD GL Gn bD bL bn
## GD  D  G  D  D  G  D
## GL  G  L  L  G  L  L
## Gn  D  L  B  D  L  B
## bD  D  G  D  B  G  B
## bL  G  L  L  B  B  B
## bn  D  L  B  B  B  B
```

We use simulations to find allele frequencies that fit field morph frequencies. The first round scans in allele frequency steps of 5% to explore the full range of possibilities, the second round scans in steps of 2% and the third in steps of 1% to test for the best combination in a restricted range. The output shows a column *Dev* with the sum of squared deviation between predictions and field morph frequencies, where lower values indicate a better fit (the top 5 results are shown).

```
AFSim = AFsimOligoFnc(gen, AFstep=0.05, nLoci=2, FieldFreq=FieldFreq,
                 allelesL1 = c("G", "b"), allelesL2 = c("D", "L", "n"))
head(AFSim[order(AFSim$Dev),], 5)
```

```
##         G    b    D    L    n PhenB PhenD PhenG PhenL          Dev
## 252 0.25 0.75 0.70 0.05 0.25  0.57  0.37  0.05  0.01 0.0001900549
## 177 0.30 0.70 0.50 0.05 0.45  0.58  0.36  0.04  0.02 0.0003509928
```

```
## 233 0.25 0.75 0.65 0.05 0.30  0.58  0.36  0.05  0.01 0.0004163410
## 271 0.25 0.75 0.75 0.05 0.20  0.56  0.38  0.05  0.01 0.0005001966
## 196 0.30 0.70 0.55 0.05 0.40  0.56  0.38  0.04  0.02 0.0005417203
```

```
AFSim = AFsimOligoFnc(gen, AFstep=0.02, nLoci=2, FieldFreq=FieldFreq,
                 allelesL1 = c("G", "b"), allelesL2 = c("D", "L", "n"),
                 AFmax = c(0.38, 0.8, 0.8, 0.1, 0.5)  )
head(AFSim[order(AFSim$Dev),], 5)
```

```
##         G    b    D    L    n PhenB PhenD PhenG PhenL          Dev
## 3641 0.30 0.70 0.52 0.04 0.44  0.58  0.37  0.03  0.02 2.696203e-05
## 3933 0.26 0.74 0.64 0.04 0.32  0.58  0.37  0.04  0.01 2.799999e-05
## 4226 0.24 0.76 0.76 0.04 0.20  0.58  0.37  0.04  0.01 2.948296e-05
## 3787 0.28 0.72 0.58 0.04 0.38  0.58  0.37  0.03  0.02 3.011709e-05
## 3544 0.32 0.68 0.48 0.04 0.48  0.58  0.37  0.03  0.02 3.183507e-05
```

```
AFSim = AFsimOligoFnc(gen, AFstep=0.01, nLoci=2, FieldFreq=FieldFreq,
                 allelesL1 = c("G", "b"), allelesL2 = c("D", "L", "n"),
                 AFmax = c(0.35, 0.8, 0.8, 0.1, 0.5)  )
head(AFSim[order(AFSim$Dev),], 5)
```

```
##          G    b    D    L    n PhenB PhenD PhenG PhenL          Dev
## 27051 0.24 0.76 0.76 0.03 0.21  0.58  0.38  0.03  0.01 1.285729e-05
## 24287 0.32 0.68 0.48 0.03 0.49  0.58  0.38  0.02  0.02 1.403518e-05
## 24484 0.31 0.69 0.50 0.03 0.47  0.58  0.38  0.02  0.02 1.424666e-05
## 24977 0.29 0.71 0.55 0.03 0.42  0.58  0.38  0.02  0.01 1.562117e-05
## 26952 0.24 0.76 0.75 0.03 0.22  0.59  0.38  0.03  0.01 1.628902e-05
```

```
cat("Combinations within an order of magnitude of best combination: ",
             sum(AFSim$Dev<=min(AFSim$Dev)*10))
```

```
## Combinations within an order of magnitude of best combination:  85
```

A reasonable fit to field morph frequencies can be achieved with allele frequencies of $p_G = 0.24$, $p_b = 0.76$, $p_D = 0.76$, $p_L = 0.03$ and $p_n = 0.21$. Larger values of p_G combinde with smaller values for p_D also give a reasonable fit, but the conclusions are qualitatively unaffected.

```
gen$Freq = NA
AF = c(G=0.24, b=0.76, D=0.76, L=0.03, n=0.21)
#AF = c(G=0.32, b=0.68, D=0.48, L=0.03, L=0.49) # An alternative
gen$Freq = AF[substr(gen$Genotype,1,1)] * AF[substr(gen$Genotype,2,2)] *
           AF[substr(gen$Genotype,3,3)] * AF[substr(gen$Genotype,4,4)]
if(round(sum(gen$Freq),10)!=1)
  print("Warning: Genotype frequencies do not add to 1")
rbind(Simulated=round(tapply(gen$Freq, gen$Phenotype, sum),2),
      Observed=round(FieldFreq,2))
```

```
##              B    D    G    L
## Simulated 0.58 0.38 0.03 0.01
## Observed  0.58 0.38 0.03 0.02
```

Now parents are sampled from this population according to the implemented mating design. Offspring genotypes are sampled from parental genotypes, offspring phenotypes determined, and the results tabulated. The process is repeated *nruns* times. Simulation results are plotted (dots) along with the observed number of offspring (bars) per mating combination. The following table shows the proportion of simulations that are as extreme as or more extreme than the observed number of offspring (a p value for the probability that the observations arose by sampling variation given the mating design). Only mating combinations in which at least one offspring morph shows $p < 0.05$ are shown.

```
nruns=1000
res = simOligoFnc(gen, dat, FemaleUq, MaleUq, nruns)
pVals = plotFnc(obs, res)
```

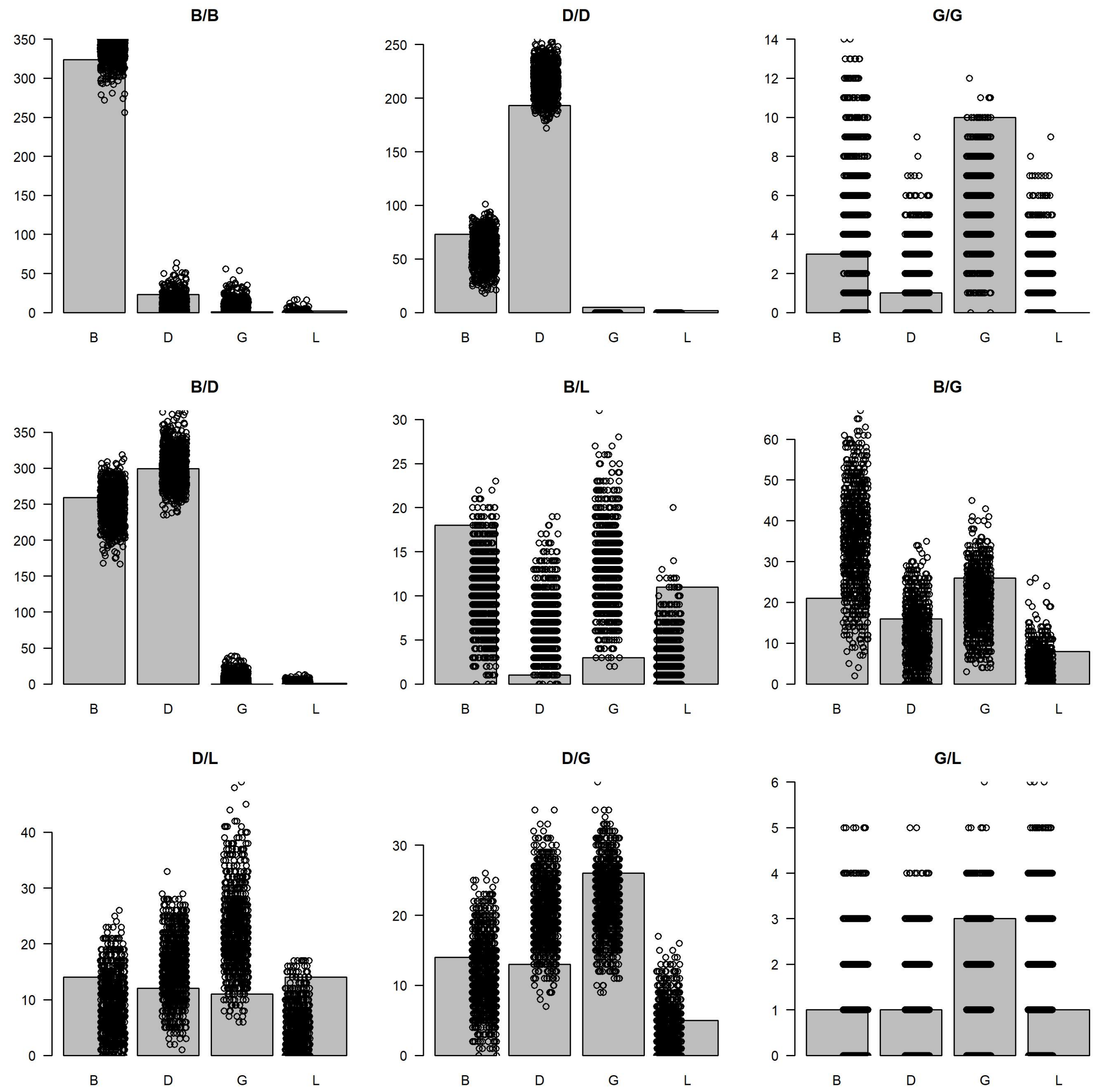


```
evalTab["Model 5", 6:11] = modevalFnc(obs, res)
```

```
## Model fit in offspring numbers and relative improvement to null (uniform) model
## Absolute deviations:             213 (84.6%)
## Sq root of squared deviations: 47 (88.8%)
## Sum of squared deviations:     2223 (98.8%)
```

```
pVals[apply(pVals[,5:8],1,min)<0.05, 5:12]
```

```
##        pB    pD    pG    pL Bsim0 Dsim0 Gsim0 Lsim0
## B/B 0.160 0.119 0.454 0.046 FALSE FALSE FALSE FALSE
```

```
## D/D 0.150 0.065 0.000 0.000 FALSE FALSE  TRUE  TRUE
## G/G 0.396 0.419 0.024 0.232 FALSE FALSE FALSE FALSE
## B/L 0.055 0.036 0.007 0.018 FALSE FALSE FALSE FALSE
## D/L 0.196 0.252 0.034 0.031 FALSE FALSE FALSE FALSE
```

The model predicts offspring morph frequencies that appear to be an overall good fit to the observed numbers. However, the model does not allow from G or L offspring from D/D matings, since both parents lack the L allele required to produce a uniform green phenotype. The number of G offspring from G/G matings is slightly underpredicted, for B/L matings the number of G and D offspring are slightly overpredicted and the number of L offspring underpredicted, for D/L matings the number of G offspring is slightly overpredicted and the number of L offspring underpredicted, and for D/G matings the number of D offspring from is slightly overpredicted. However, these differences seem more quantitative rather than qualitative.

Alternative allele frequencies of $p_G = 0.32$, $p_b = 0.68$, $p_D = 0.48$, $p_L = 0.03$ and $p_n = 0.48$ show the same issues with D/D matings, an significant underprediction of G offspring from B/G matings and a significant overprediction of D offspring from D/G matings. These patterns are thus incompatible with the observed data.

Three-locus models

Model 6: Three-locus models with two alleles (U and N dominant)

There might be three loci involved in color morph determination: One locus G with two alleles (G and b) might be responsible for the ability to produce green color, with G dominant over b. One locus D with two alleles (U and d) that suppresses green color dorsally, with U (the suppressor) dominant over d. One locus L with two alleles (N and l) that suppresses green color laterally, with N (the suppressor) dominant over l. There are $(2 * 2)^3 = 64$ possible genotypes ($3 * 3 * 3 = 27$ of them unique). Despite the large number of genotypes, there are only three rules to be defined: the order of dominance at the three loci.

```
gen = data.frame(Genotype=rep(NA,64), Phenotype=NA, Freq=NA)
i = 1
for(g1 in c("G", "b"))
  for(g2 in c("G", "b"))
    for(d1 in c("U", "d"))
      for(d2 in c("U", "d"))
        for(l1 in c("N", "l"))
          for(l2 in c("N", "l")) {
            gen$Genotype[i] = paste0(g1, g2, d1, d2, l1, l2)
            i = i + 1
          }
gen$Phenotype[is.na(gen$Phenotype) &
              (substr(gen$Genotype,1,1)=="b" & substr(gen$Genotype,2,2)=="b")] = "B"
gen$Phenotype[is.na(gen$Phenotype) &
              (substr(gen$Genotype,3,3)=="d" & substr(gen$Genotype,4,4)=="d") &
              (substr(gen$Genotype,5,5)=="l" & substr(gen$Genotype,6,6)=="l")] = "G"
gen$Phenotype[is.na(gen$Phenotype) &
              (substr(gen$Genotype,3,3)=="U" | substr(gen$Genotype,4,4)=="U") &
              (substr(gen$Genotype,5,5)=="N" | substr(gen$Genotype,6,6)=="N")] = "B"
gen$Phenotype[is.na(gen$Phenotype) &
              (substr(gen$Genotype,3,3)=="U" | substr(gen$Genotype,4,4)=="U")] = "L"
gen$Phenotype[is.na(gen$Phenotype) &
              (substr(gen$Genotype,5,5)=="N" | substr(gen$Genotype,6,6)=="N")] = "D"
```

These genotypes produce phenotypes as follows.

```
PunnettSquare(gen, nloci=3)
```

```
##     GUN GUl GdN Gdl bUN bUl bdN bdl
## GUN   B   B   B   B   B   B   B   B
## GUl   B   L   B   L   B   L   B   L
## GdN   B   B   D   D   B   B   D   D
## Gdl   B   L   D   G   B   L   D   G
## bUN   B   B   B   B   B   B   B   B
## bUl   B   L   B   L   B   B   B   B
## bdN   B   B   D   D   B   B   B   B
## bdl   B   L   D   G   B   B   B   B
```

We use simulations to find allele frequencies that fit field morph frequencies. The first round scans in allele frequency steps of 5% to explore the full range of possibilities, the second round scans in steps of 2% and the third in steps of 1% to test for the best combination in a restricted range. The output shows a column *Dev* with the sum of squared deviation between predictions and field morph frequencies, where lower values indicate a better fit (the top 5 results are shown).

```r
AFSim = AFsimOligoFnc(gen, AFstep=0.05, nLoci=3, FieldFreq=FieldFreq,
                 allelesL1 = c("G", "b"), allelesL2 = c("U", "d"), allelesL3 = c("N", "l"))
head(AFSim[order(AFSim$Dev),], 5)
```

```
##         G    b    U    d    N    l PhenB PhenD PhenG PhenL          Dev
## 4718 0.30 0.70 0.10 0.90 0.70 0.30  0.58  0.38  0.04  0.01 9.242044e-06
## 5544 0.75 0.25 0.35 0.65 0.80 0.20  0.58  0.38  0.02  0.02 2.147030e-05
## 5160 0.55 0.45 0.30 0.70 0.75 0.25  0.58  0.37  0.02  0.03 9.474267e-05
## 5119 0.40 0.60 0.20 0.80 0.75 0.25  0.58  0.38  0.03  0.01 9.550772e-05
## 5139 0.45 0.55 0.25 0.75 0.75 0.25  0.59  0.37  0.02  0.02 1.169201e-04
```

```r
AFSim = AFsimOligoFnc(gen, AFstep=0.02, nLoci=3, FieldFreq=FieldFreq,
                 allelesL1 = c("G", "b"), allelesL2 = c("U", "d"), allelesL3 = c("N", "l"),
                 AFmax = c(0.80, 0.74, 0.38, 0.94, 0.84, 0.34))
head(AFSim[order(AFSim$Dev),], 5)
```

```
##         G    b    U    d    N    l PhenB PhenD PhenG PhenL          Dev
## 2504 0.48 0.52 0.26 0.74 0.76 0.24  0.58  0.38  0.02  0.02 6.722337e-07
## 3043 0.62 0.38 0.32 0.68 0.78 0.22  0.58  0.38  0.02  0.02 1.358636e-06
## 2534 0.52 0.48 0.28 0.72 0.76 0.24  0.58  0.38  0.02  0.02 2.379679e-06
## 3108 0.80 0.20 0.36 0.64 0.78 0.22  0.58  0.37  0.02  0.03 5.866731e-06
## 1997 0.42 0.58 0.22 0.78 0.74 0.26  0.58  0.38  0.03  0.02 6.823766e-06
```

```r
AFSim = AFsimOligoFnc(gen, AFstep=0.01, nLoci=3, FieldFreq=FieldFreq,
                 allelesL1 = c("G", "b"), allelesL2 = c("U", "d"), allelesL3 = c("N", "l"),
                 AFmax = c(0.79, 0.74, 0.38, 0.94, 0.84, 0.34))
head(AFSim[order(AFSim$Dev),], 5)
```

```
##          G    b    U    d    N    l PhenB PhenD PhenG PhenL          Dev
## 10754 0.33 0.67 0.14 0.86 0.72 0.28  0.58  0.38  0.03  0.01 1.248277e-08
## 20338 0.59 0.41 0.31 0.69 0.77 0.23  0.58  0.38  0.02  0.02 4.954079e-07
## 18329 0.48 0.52 0.26 0.74 0.76 0.24  0.58  0.38  0.02  0.02 6.722337e-07
## 20225 0.54 0.46 0.29 0.71 0.77 0.23  0.58  0.38  0.02  0.02 9.065037e-07
## 14485 0.38 0.62 0.19 0.81 0.74 0.26  0.58  0.38  0.03  0.01 1.072882e-06
```

```r
cat("Combinations within an order of magnitude of best combination: ",
             sum(AFSim$Dev<=min(AFSim$Dev)*10))
```

```
## Combinations within an order of magnitude of best combination:  1
```

A reasonable fit to field morph frequencies can be achieved with allele frequencies of $p_G = 0.33$, $p_b = 0.67$, $p_U = 0.14$, $p_d = 0.86$, $p_N = 0.72$ and $p_l = 0.28$.

```r
AF = c(G=0.33, b=0.67, U=0.14, d=0.86, N=0.72, l=0.28)
gen$Freq = AF[substr(gen$Genotype,1,1)] * AF[substr(gen$Genotype,2,2)] *
           AF[substr(gen$Genotype,3,3)] * AF[substr(gen$Genotype,4,4)] *
           AF[substr(gen$Genotype,5,5)] * AF[substr(gen$Genotype,6,6)]
if(round(sum(gen$Freq),10)!=1)
  print("Warning: Genotype frequencies do not add to 1")
rbind(Simulated=round(tapply(gen$Freq, gen$Phenotype, sum),2),
      Observed=round(FieldFreq,2))
```

```
##              B    D    G    L
## Simulated 0.58 0.38 0.03 0.01
## Observed  0.58 0.38 0.03 0.02
```

Now parents are sampled from this population according to the implemented mating design. Offspring genotypes are sampled from parental genotypes, offspring phenotypes determined, and the results tabulated.

The process is repeated *nruns* times. Simulation results are plotted (dots) along with the observed number of offspring (bars) per mating combination. The following table shows the proportion of simulations that are as extreme as or more extreme than the observed number of offspring (a p value for the probability that the observations arose by sampling variation given the mating design). Only mating combinations in which at least one offspring morph shows $p < 0.05$ are shown.

```
nruns=1000
res = simOligoFnc(gen, dat, FemaleUq, MaleUq, nruns)
pVals = plotFnc(obs, res)
```

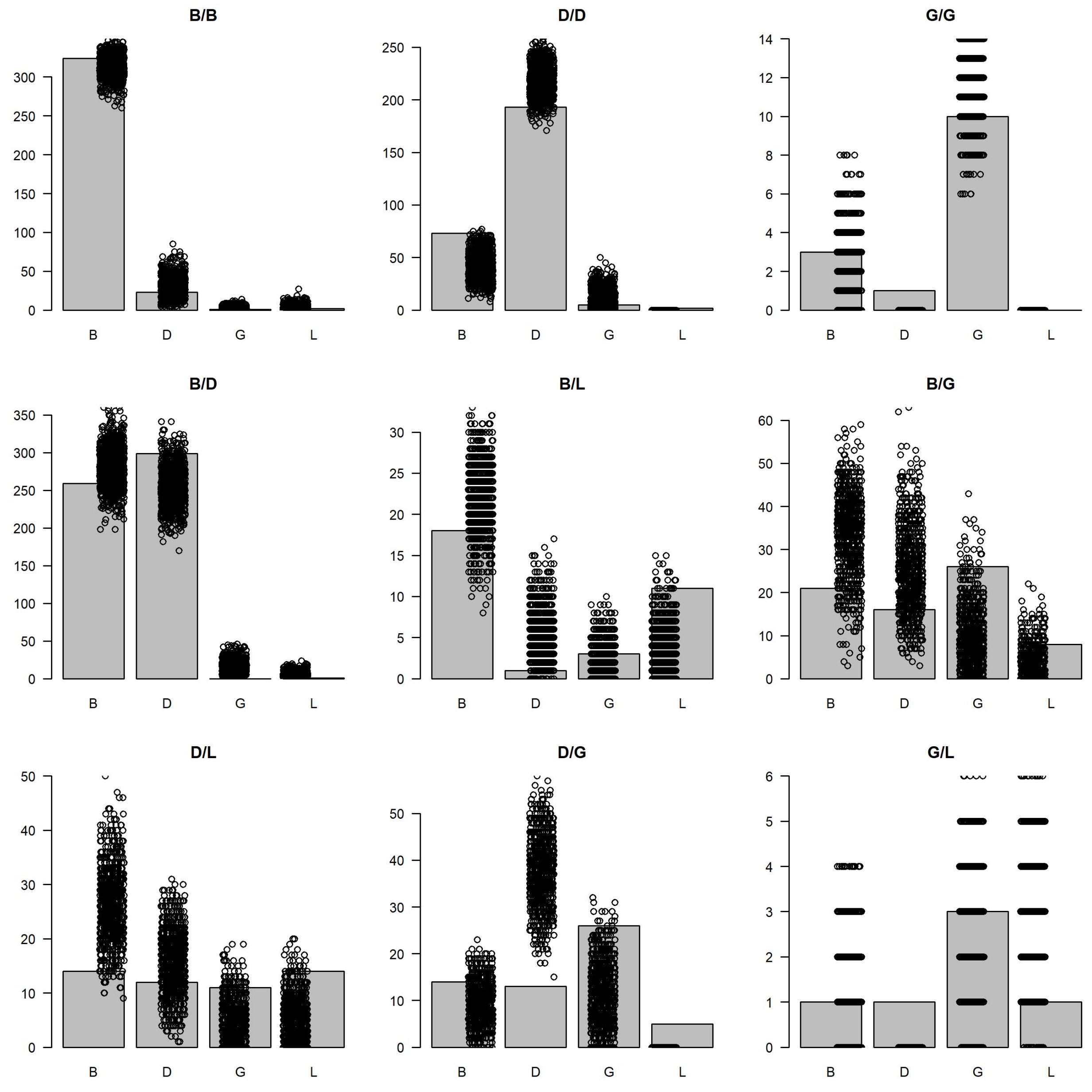


```
evalTab["Model 6", 6:11] = modevalFnc(obs, res)
```

```
## Model fit in offspring numbers and relative improvement to null (uniform) model
## Absolute deviations:            310 (77.6%)
## Sq root of squared deviations: 77 (81.7%)
```

```
## Sum of squared deviations:     5967 (96.6%)
```

```
pVals[apply(pVals[,5:8],1,min)<0.05, 5:12]
```

```
##        pB    pD    pG    pL Bsim0 Dsim0 Gsim0 Lsim0
## D/D 0.005 0.045 0.316 0.000 FALSE FALSE FALSE  TRUE
## G/G 0.452 0.000 0.278 1.000 FALSE  TRUE FALSE FALSE
## B/D 0.185 0.055 0.006 0.198 FALSE FALSE FALSE FALSE
## B/L 0.184 0.068 0.310 0.020 FALSE FALSE FALSE FALSE
## B/G 0.099 0.182 0.026 0.102 FALSE FALSE FALSE FALSE
## D/L 0.028 0.284 0.072 0.035 FALSE FALSE FALSE FALSE
## D/G 0.171 0.000 0.014 0.000 FALSE FALSE FALSE  TRUE
## G/L 0.585 0.000 0.465 0.181 FALSE  TRUE FALSE FALSE
```

The model predicts offspring morph frequencies that are significantly different from observed numbers for several mating combinations. In particular the model does not allow for L offspring from D/D and D/G mating combinations (due to lack of an N allele) and it does not allow for D offspring from G/G and G/L mating combinations (due to lack of an U allele). Several other numbers are significantly over- or underpredicted. These patterns are thus incompatible with the observed data.

Model 7: Three-locus models with two alleles (D and L dominant)

There might be three loci involved in color morph determination: One locus G with two alleles (G and b) might be responsible for the ability to produce green color, with G dominant over b. One locus D with two alleles (D and u) where the recessive allele u suppresses green color dorsally. One locus L with two alleles (L and n) where the recessive allele n suppresses green color laterally.

```
gen = data.frame(Genotype=rep(NA,64), Phenotype=NA, Freq=NA)
i = 1
for(g1 in c("G", "b"))
  for(g2 in c("G", "b"))
    for(d1 in c("D", "u"))
      for(d2 in c("D", "u"))
        for(l1 in c("L", "n"))
          for(l2 in c("L", "n")) {
            gen$Genotype[i] = paste0(g1, g2, d1, d2, l1, l2)
            i = i + 1
          }
gen$Phenotype[is.na(gen$Phenotype) &
              (substr(gen$Genotype,1,1)=="b" & substr(gen$Genotype,2,2)=="b")] = "B"
gen$Phenotype[is.na(gen$Phenotype) &
              (substr(gen$Genotype,3,3)=="u" & substr(gen$Genotype,4,4)=="u") &
              (substr(gen$Genotype,5,5)=="n" & substr(gen$Genotype,6,6)=="n")] = "B"
gen$Phenotype[is.na(gen$Phenotype) &
              (substr(gen$Genotype,3,3)=="D" | substr(gen$Genotype,4,4)=="D") &
              (substr(gen$Genotype,5,5)=="L" | substr(gen$Genotype,6,6)=="L")] = "G"
gen$Phenotype[is.na(gen$Phenotype) &
              (substr(gen$Genotype,3,3)=="D" | substr(gen$Genotype,4,4)=="D")] = "D"
gen$Phenotype[is.na(gen$Phenotype) &
              (substr(gen$Genotype,5,5)=="L" | substr(gen$Genotype,6,6)=="L")] = "L"
```

These genotypes would produce phenotypes as follows.

```
PunnettSquare(gen, nloci=3)
```

```
##     GDL GDn GuL Gun bDL bDn buL bun
## GDL   G   G   G   G   G   G   G   G
## GDn   G   D   G   D   G   D   G   D
## GuL   G   G   L   L   G   G   L   L
## Gun   G   D   L   B   G   D   L   B
## bDL   G   G   G   G   B   B   B   B
## bDn   G   D   G   D   B   B   B   B
## buL   G   G   L   L   B   B   B   B
## bun   G   D   L   B   B   B   B   B
```

We use simulations to find allele frequencies that fit field morph frequencies. The first round scans in allele frequency steps of 5% to explore the full range of possibilities, the second round scans in steps of 2% and the third in steps of 1% to test for the best combination in a restricted range. The output shows a column *Dev* with the sum of squared deviation between predictions and field morph frequencies, where lower values indicate a better fit (the top 5 results are shown).

```
AFSim = AFsimOligoFnc(gen, AFstep=0.05, nLoci=3, FieldFreq=FieldFreq,
                 allelesL1 = c("G", "b"), allelesL2 = c("D", "u"), allelesL3 = c("L", "n"))
head(AFSim[order(AFSim$Dev),], 5)
```

```
##        G    b    D    u    L    n PhenB PhenD PhenG PhenL          Dev
## 290 0.25 0.75 0.80 0.20 0.05 0.95  0.58  0.38  0.04  0.00 1.952063e-05
```

```
## 271 0.25 0.75 0.75 0.25 0.05 0.95  0.59  0.37  0.04  0.00 6.657523e-05
## 196 0.30 0.70 0.55 0.45 0.05 0.95  0.58  0.37  0.04  0.01 7.904388e-05
## 159 0.35 0.65 0.45 0.55 0.05 0.95  0.58  0.36  0.04  0.02 1.499768e-04
## 309 0.25 0.75 0.85 0.15 0.05 0.95  0.57  0.39  0.04  0.00 2.011848e-04
```

```r
AFSim = AFsimOligoFnc(gen, AFstep=0.02, nLoci=3, FieldFreq=FieldFreq,
                 allelesL1 = c("G", "b"), allelesL2 = c("D", "u"), allelesL3 = c("L", "n"),
                 AFmax = c(0.4, 0.8, 0.9, 0.6, 0.1, 1.0))
head(AFSim[order(AFSim$Dev),], 5)
```

```
##        G    b    D    u    L    n PhenB PhenD PhenG PhenL          Dev
## 317 0.36 0.64 0.44 0.56 0.04 0.96  0.58  0.37  0.03  0.01 6.044334e-06
## 380 0.30 0.70 0.56 0.44 0.04 0.96  0.58  0.38  0.03  0.01 1.085229e-05
## 412 0.28 0.72 0.62 0.38 0.04 0.96  0.58  0.38  0.03  0.01 1.777519e-05
## 455 0.26 0.74 0.70 0.30 0.04 0.96  0.59  0.38  0.03  0.00 2.879162e-05
## 307 0.38 0.62 0.42 0.58 0.04 0.96  0.58  0.38  0.03  0.02 3.609928e-05
```

```r
AFSim = AFsimOligoFnc(gen, AFstep=0.01, nLoci=3, FieldFreq=FieldFreq,
                 allelesL1 = c("G", "b"), allelesL2 = c("D", "u"), allelesL3 = c("L", "n"),
                 AFmax = c(0.39, 0.77, 0.87, 0.59, 0.07, 0.97))
head(AFSim[order(AFSim$Dev),], 5)
```

```
##         G    b    D    u    L    n PhenB PhenD PhenG PhenL          Dev
## 147  0.33 0.67 0.49 0.51 0.04 0.96  0.58  0.38  0.03  0.01 4.737811e-08
## 1431 0.25 0.75 0.78 0.22 0.05 0.95  0.58  0.38  0.04  0.00 1.577127e-07
## 180  0.32 0.68 0.51 0.49 0.04 0.96  0.58  0.38  0.03  0.01 5.989211e-07
## 213  0.31 0.69 0.53 0.47 0.04 0.96  0.58  0.38  0.03  0.01 2.571033e-06
## 1330 0.26 0.74 0.72 0.28 0.05 0.95  0.58  0.38  0.04  0.00 2.869900e-06
```

```r
cat("Combinations within an order of magnitude of best combination: ",
             sum(AFSim$Dev<=min(AFSim$Dev)*10))
```

```
## Combinations within an order of magnitude of best combination:  2
```

A reasonable fit to field morph frequencies can be achieved with allele frequencies of $p_G = 0.33$, $p_b = 0.67$, $p_D = 0.49$, $p_u = 0.51$, $p_L = 0.04$ and $p_n = 0.96$.

```r
AF = c(G=0.33, b=0.67, D=0.49, u=0.51, L=0.04, n=0.96)
gen$Freq = AF[substr(gen$Genotype,1,1)] * AF[substr(gen$Genotype,2,2)] *
           AF[substr(gen$Genotype,3,3)] * AF[substr(gen$Genotype,4,4)] *
           AF[substr(gen$Genotype,5,5)] * AF[substr(gen$Genotype,6,6)]
if(round(sum(gen$Freq),10)!=1)
  print("Warning: Genotype frequencies do not add to 1")
rbind(Simulated=round(tapply(gen$Freq, gen$Phenotype, sum),2),
      Observed=round(FieldFreq,2))
```

```
##              B    D    G    L
## Simulated 0.58 0.38 0.03 0.01
## Observed  0.58 0.38 0.03 0.02
```

Now parents are sampled from this population according to the implemented mating design. Offspring genotypes are sampled from parental genotypes, offspring phenotypes determined, and the results tabulated. The process is repeated *nruns* times. Simulation results are plotted (dots) along with the observed number of offspring (bars) per mating combination. The following table shows the proportion of simulations that are as extreme or more extreme than the observed number of offspring (a p value for the probability that the observations arose by sampling variation given the mating design). Only mating combinations in which at least one offspring morph shows $p < 0.05$ are shown.

```
nruns=1000
res = simOligoFnc(gen, dat, FemaleUq, MaleUq, nruns)
pVals = plotFnc(obs, res)
```

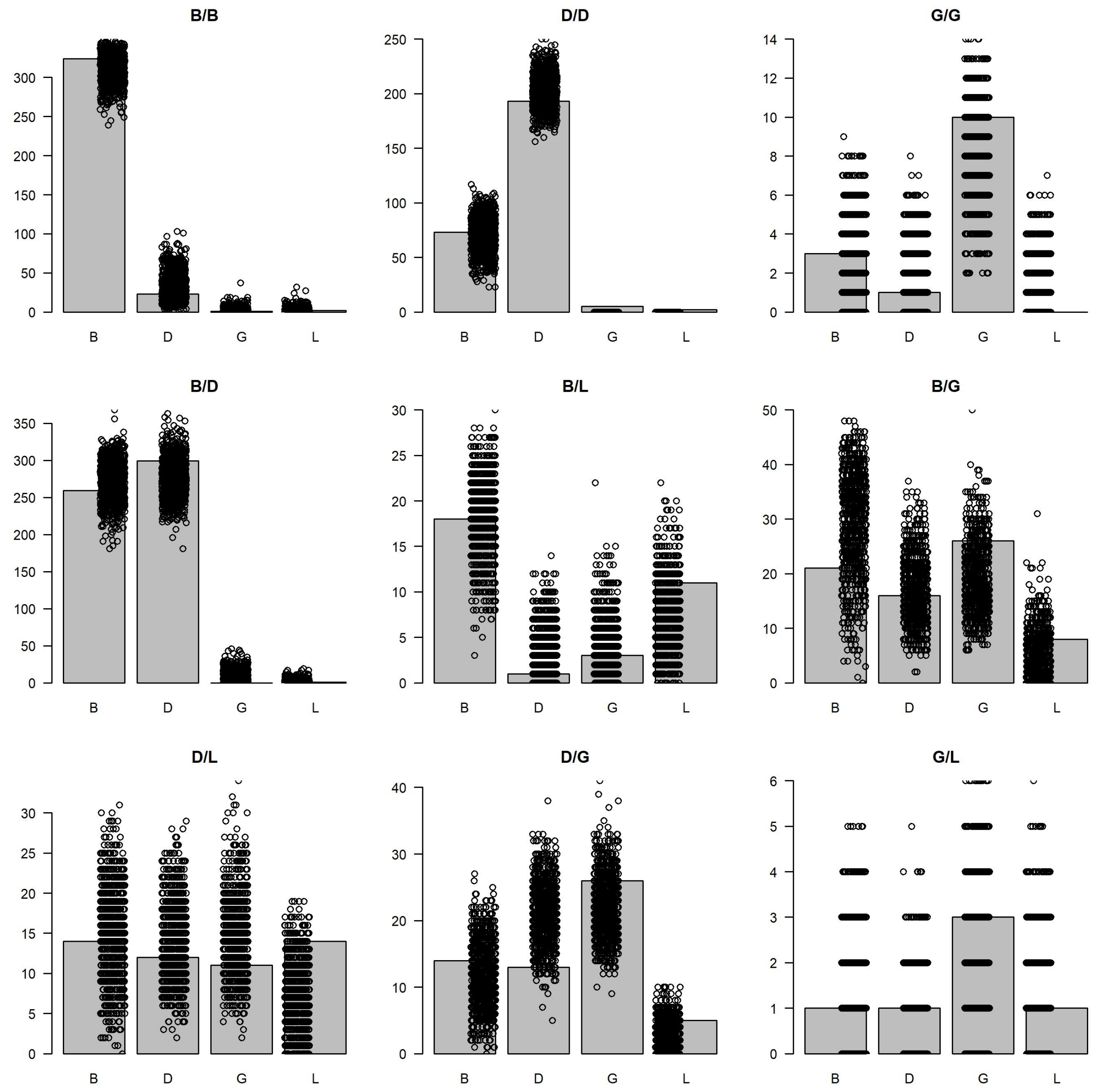


```
evalTab["Model 7", 6:11] = modevalFnc(obs, res)
```

```
## Model fit in offspring numbers and relative improvement to null (uniform) model
## Absolute deviations:             148 (89.3%)
## Sq root of squared deviations: 38 (90.9%)
## Sum of squared deviations:     1478 (99.2%)
```

```
pVals[apply(pVals[,5:8],1,min)<0.05, 5:12]
```

```
##         pB    pD   pG    pL Bsim0 Dsim0 Gsim0 Lsim0
## D/D 0.457 0.301 0.00 0.000 FALSE FALSE  TRUE  TRUE
```

```
## D/G 0.386 0.045 0.19 0.204 FALSE FALSE FALSE FALSE
```

The model predicts offspring morph frequencies that appear to be an overall good fit to the observed numbers. The only exception is that there are no G or L offspring possible from D/D, because they lack the L allele. Furthermore, the number of D offspring from D/G mating combinations is slightly overpredicted (though within the margins expected with multiple testing).

Model 8: Three-locus models with two alleles (D and N dominant)

There might be three loci involved in color morph determination: One locus G with two alleles (G and b) might be responsible for the ability to produce green color, with G dominant over b. One locus D with two alleles (D and u) where the recessive allele u suppresses green color dorsally. One locus L with two alleles (l and N) where the dominant allele N suppresses green color laterally.

```
gen = data.frame(Genotype=rep(NA,64), Phenotype=NA, Freq=NA)
i = 1
for(g1 in c("G", "b"))
  for(g2 in c("G", "b"))
    for(d1 in c("D", "u"))
      for(d2 in c("D", "u"))
        for(l1 in c("l", "N"))
          for(l2 in c("l", "N")) {
            gen$Genotype[i] = paste0(g1, g2, d1, d2, l1, l2)
            i = i + 1
          }
gen$Phenotype[is.na(gen$Phenotype) &
              (substr(gen$Genotype,1,1)=="b" & substr(gen$Genotype,2,2)=="b")] = "B"
gen$Phenotype[is.na(gen$Phenotype) &
              (substr(gen$Genotype,3,3)=="u" & substr(gen$Genotype,4,4)=="u") &
              (substr(gen$Genotype,5,5)=="N" | substr(gen$Genotype,6,6)=="N")] = "B"
gen$Phenotype[is.na(gen$Phenotype) &
              (substr(gen$Genotype,3,3)=="D" | substr(gen$Genotype,4,4)=="D") &
              (substr(gen$Genotype,5,5)=="l" & substr(gen$Genotype,6,6)=="l")] = "G"
gen$Phenotype[is.na(gen$Phenotype) &
              (substr(gen$Genotype,3,3)=="D" | substr(gen$Genotype,4,4)=="D")] = "D"
gen$Phenotype[is.na(gen$Phenotype) &
              (substr(gen$Genotype,5,5)=="l" & substr(gen$Genotype,6,6)=="l")] = "L"
```

These genotypes would produce phenotypes as follows.

```
PunnettSquare(gen, nloci=3)
```

```
##     GDl GDN Gul GuN bDl bDN bul buN
## GDl   G   D   G   D   G   D   G   D
## GDN   D   D   D   D   D   D   D   D
## Gul   G   D   L   B   G   D   L   B
## GuN   D   D   B   B   D   D   B   B
## bDl   G   D   G   D   B   B   B   B
## bDN   D   D   D   D   B   B   B   B
## bul   G   D   L   B   B   B   B   B
## buN   D   D   B   B   B   B   B   B
```

We use simulations to find allele frequencies that fit field morph frequencies. The first round scans in allele frequency steps of 5% to explore the full range of possibilities, the second round scans in steps of 2% and the third in steps of 1% to test for the best combination in a restricted range. The output shows a column *Dev* with the sum of squared deviation between predictions and field morph frequencies, where lower values indicate a better fit (the top 5 results are shown).

```
AFSim = AFsimOligoFnc(gen, AFstep=0.05, nLoci=3, FieldFreq=FieldFreq,
                  allelesL1 = c("G", "b"), allelesL2 = c("D", "u"), allelesL3 = c("l", "N"))
head(AFSim[order(AFSim$Dev),], 5)
```

```
##         G    b    D    u    l    N PhenB PhenD PhenG PhenL          Dev
## 1567 0.45 0.55 0.35 0.65 0.25 0.75  0.58  0.38  0.03  0.02 9.542062e-06
```

```
## 1603 0.35 0.65 0.45 0.55 0.25 0.75  0.59  0.38  0.03  0.01 2.922331e-05
## 2001 0.30 0.70 0.55 0.45 0.30 0.70  0.58  0.37  0.04  0.01 3.917415e-05
## 1173 0.70 0.30 0.25 0.75 0.20 0.80  0.58  0.38  0.02  0.02 4.184588e-05
## 2076 0.25 0.75 0.75 0.25 0.30 0.70  0.59  0.37  0.04  0.00 4.427120e-05
```

```
AFSim = AFsimOligoFnc(gen, AFstep=0.02, nLoci=3, FieldFreq=FieldFreq,
                 allelesL1 = c("G", "b"), allelesL2 = c("D", "u"), allelesL3 = c("l", "N"),
                 AFmax = c(0.78, 0.78, 0.78, 0.8, 0.36, 0.8))
head(AFSim[order(AFSim$Dev),], 5)
```

```
##         G    b    D    u    l    N PhenB PhenD PhenG PhenL          Dev
## 4963 0.28 0.72 0.62 0.38 0.30 0.70  0.58  0.37  0.04  0.01 8.023852e-07
## 955  0.74 0.26 0.24 0.76 0.22 0.78  0.58  0.37  0.02  0.03 2.168770e-06
## 1956 0.46 0.54 0.34 0.66 0.24 0.76  0.58  0.38  0.02  0.02 2.537995e-06
## 4877 0.30 0.70 0.56 0.44 0.30 0.70  0.58  0.37  0.04  0.01 4.060375e-06
## 979  0.64 0.36 0.26 0.74 0.22 0.78  0.58  0.37  0.02  0.02 4.844953e-06
```

```
AFSim = AFsimOligoFnc(gen, AFstep=0.01, nLoci=3, FieldFreq=FieldFreq,
                 allelesL1 = c("G", "b"), allelesL2 = c("D", "u"), allelesL3 = c("l", "N"),
                 AFmax = c(0.75, 0.75, 0.73, 0.77, 0.33, 0.79))
head(AFSim[order(AFSim$Dev),], 5)
```

```
##          G    b    D    u    l    N PhenB PhenD PhenG PhenL          Dev
## 16689 0.36 0.64 0.44 0.56 0.27 0.73  0.58  0.38  0.03  0.01 2.179854e-09
## 19542 0.33 0.67 0.49 0.51 0.28 0.72  0.58  0.38  0.03  0.01 4.737811e-08
## 5491  0.58 0.42 0.28 0.72 0.23 0.77  0.58  0.38  0.02  0.02 2.029697e-07
## 8238  0.51 0.49 0.31 0.69 0.24 0.76  0.58  0.38  0.02  0.02 3.332059e-07
## 19643 0.32 0.68 0.51 0.49 0.28 0.72  0.58  0.38  0.03  0.01 5.989211e-07
```

```
cat("Combinations within an order of magnitude of best combination: ",
             sum(AFSim$Dev<=min(AFSim$Dev)*10))
```

```
## Combinations within an order of magnitude of best combination:  1
```

A reasonable fit to field morph frequencies can be achieved with allele frequencies of $p_G = 0.36$, $p_b = 0.64$, $p_D = 0.44$, $p_u = 0.56$, $p_L = 0.27$ and $p_n = 0.73$.

```
AF = c(G=0.36, b=0.64, D=0.44, u=0.56, l=0.27, N=0.73)
gen$Freq = AF[substr(gen$Genotype,1,1)] * AF[substr(gen$Genotype,2,2)] *
           AF[substr(gen$Genotype,3,3)] * AF[substr(gen$Genotype,4,4)] *
           AF[substr(gen$Genotype,5,5)] * AF[substr(gen$Genotype,6,6)]
if(round(sum(gen$Freq),10)!=1)
  print("Warning: Genotype frequencies do not add to 1")
rbind(Simulated=round(tapply(gen$Freq, gen$Phenotype, sum),2),
      Observed=round(FieldFreq,2))
```

```
##              B    D    G    L
## Simulated 0.58 0.38 0.03 0.01
## Observed  0.58 0.38 0.03 0.02
```

Now parents are sampled from this population according to the implemented mating design. Offspring genotypes are sampled from parental genotypes, offspring phenotypes determined, and the results tabulated. The process is repeated *nruns* times. Simulation results are plotted (dots) along with the observed number of offspring (bars) per mating combination. The following table shows the proportion of simulations that are as extreme as or more extreme than the observed number of offspring (a p value for the probability that the observations arose by sampling variation given the mating design). Only mating combinations in which at least one offspring morph shows $p < 0.05$ are shown.

```r
nruns=1000
res = simOligoFnc(gen, dat, FemaleUq, MaleUq, nruns)
pVals = plotFnc(obs, res)
```

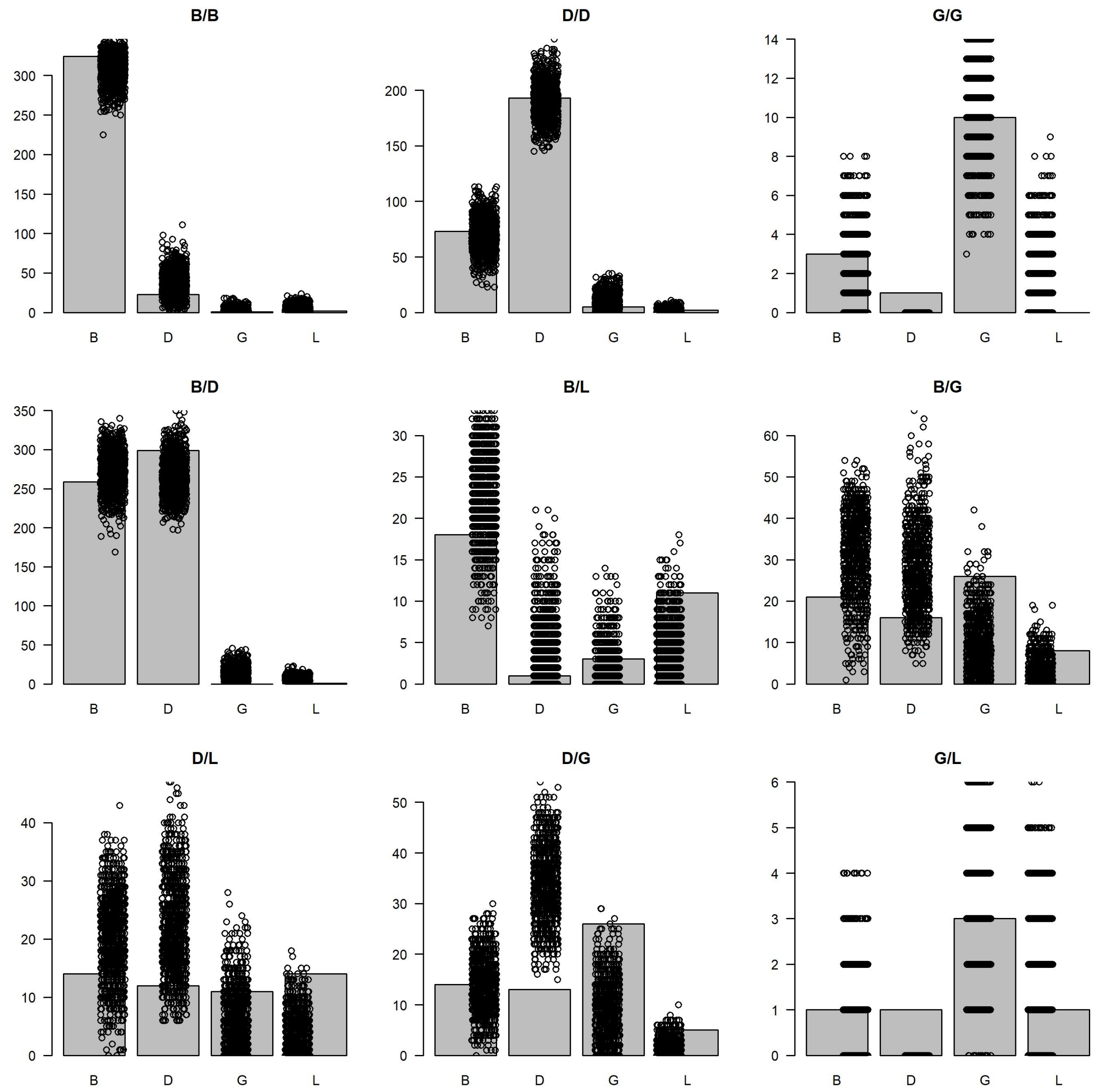


```r
evalTab["Model 8", 6:11] = modevalFnc(obs, res)
```

```
## Model fit in offspring numbers and relative improvement to null (uniform) model
## Absolute deviations:             242 (82.5%)
## Sq root of squared deviations: 60 (85.9%)
## Sum of squared deviations:     3557 (98%)
```

```r
pVals[apply(pVals[,5:8],1,min)<0.05, 5:12]
```

```
##          pB    pD    pG    pL Bsim0 Dsim0 Gsim0 Lsim0
## G/G 0.397 0.000 0.527 0.451 FALSE  TRUE FALSE FALSE
```

```
## B/D 0.310 0.110 0.002 0.153 FALSE FALSE FALSE FALSE
## B/L 0.199 0.260 0.283 0.045 FALSE FALSE FALSE FALSE
## B/G 0.135 0.117 0.017 0.102 FALSE FALSE FALSE FALSE
## D/L 0.216 0.104 0.189 0.009 FALSE FALSE FALSE FALSE
## D/G 0.498 0.000 0.004 0.066 FALSE FALSE FALSE FALSE
## G/L 0.557 0.000 0.516 0.464 FALSE  TRUE FALSE FALSE
```

The model predicts offspring morph frequencies that are significantly different from observed numbers for most mating combinations. In particular the model does not allow for D offspring from G/G and G/L mating combinations (due to lack of an N allele), it overpredicts G offspring from B/D and D/G mating combinations, it underpredicts G offspring from B/G and D/G mating combinations and it underpredicts L offspring from D/L mating combinations. The results are thus not compatible with the observed data.

Model 9: Three-locus models with two alleles (2 loci for G)

There might be three loci involved in color morph determination: One locus G with two alleles (G and B) might be responsible for the ability to produce green color, with the ability allele G dominant over b. Another locus M with two alleles (M and w) responsible for the ability to produce green color at a different place in the genetic pathway, with M dominant over w. A locus R with alleles R and r might explain the regions in which green occurs (RR for fully green, Rr for dorsal green, and rr for lateral green). There are $(2 * 2)^3 = 64$ possible genotypes ($3 * 3 * 3 = 27$ of them unique). There are only four rules to be defined: the order of dominance at *G1* and *G2* and the genetic effects at locus R.

```
gen = data.frame(Genotype=rep(NA,64), Phenotype=NA, Freq=NA)
i = 1
for(g1 in c("G", "b"))
  for(g2 in c("G", "b"))
    for(d1 in c("M", "w"))
      for(d2 in c("M", "w"))
        for(l1 in c("R", "r"))
          for(l2 in c("R", "r")) {
            gen$Genotype[i] = paste0(g1, g2, d1, d2, l1, l2)
            i = i + 1
          }
gen$Phenotype = NA
gen$Phenotype[is.na(gen$Phenotype) &
              (substr(gen$Genotype,1,1)=="b" & substr(gen$Genotype,2,2)=="b") |
              (substr(gen$Genotype,3,3)=="w" & substr(gen$Genotype,4,4)=="w")] = "B"
gen$Phenotype[is.na(gen$Phenotype) &
              (substr(gen$Genotype,5,5)=="R" & substr(gen$Genotype,6,6)=="R")] = "D"
gen$Phenotype[is.na(gen$Phenotype) &
              (substr(gen$Genotype,5,5)=="r" & substr(gen$Genotype,6,6)=="r")] = "L"
gen$Phenotype[is.na(gen$Phenotype) &
              (substr(gen$Genotype,5,5)=="R" | substr(gen$Genotype,6,6)=="R")] = "G"
```

These genotypes would produce phenotypes as follows.

```
PunnettSquare(gen, nloci=3)
```

```
##     GMR GMr GwR Gwr bMR bMr bwR bwr
## GMR   D   G   D   G   D   G   D   G
## GMr   G   L   G   L   G   L   G   L
## GwR   D   G   B   B   D   G   B   B
## Gwr   G   L   B   B   G   L   B   B
## bMR   D   G   D   G   B   B   B   B
## bMr   G   L   G   L   B   B   B   B
## bwR   D   G   B   B   B   B   B   B
## bwr   G   L   B   B   B   B   B   B
```

We use simulations to find allele frequencies that fit field morph frequencies. The first round scans in allele frequency steps of 5% to explore the full range of possibilities, the second round scans in steps of 2% and the third in steps of 1% to test for the best combination in a restricted range. The output shows a column *Dev* with the sum of squared deviation between predictions and field morph frequencies, where lower values indicate a better fit (the top 5 results are shown).

```
AFSim = AFsimOligoFnc(gen, AFstep=0.05, nLoci=3, FieldFreq=FieldFreq,
                allelesL1 = c("G", "b"), allelesL2 = c("M", "w"), allelesL3 = c("R", "r"),
                FirstExchangeble = TRUE)
head(AFSim[order(AFSim$Dev),], 5)
```

```
##          G    b    M    w    R    r PhenB PhenD PhenG PhenL          Dev
## 6590 0.80 0.20 0.25 0.75 0.95 0.05  0.58  0.38  0.04     0 1.246893e-05
## 6589 0.75 0.25 0.25 0.75 0.95 0.05  0.59  0.37  0.04     0 1.054854e-04
## 6639 0.40 0.60 0.40 0.60 0.95 0.05  0.59  0.37  0.04     0 1.212385e-04
## 6591 0.85 0.15 0.25 0.75 0.95 0.05  0.57  0.39  0.04     0 1.832383e-04
## 6605 0.60 0.40 0.30 0.70 0.95 0.05  0.57  0.39  0.04     0 2.111634e-04
```

```
AFSim = AFsimOligoFnc(gen, AFstep=0.02, nLoci=3, FieldFreq=FieldFreq,
                 allelesL1 = c("G", "b"), allelesL2 = c("M", "w"), allelesL3 = c("R", "r"),
                 AFmax = c(0.88, 0.68, 0.48, 0.8, 1.0, 0.1),
                 FirstExchangeble = TRUE)
AFSim = AFsimOligoFnc(gen, AFstep=0.01, nLoci=3, FieldFreq=FieldFreq,
                 allelesL1 = c("G", "b"), allelesL2 = c("M", "w"), allelesL3 = c("R", "r"),
                 AFmax = c(0.85, 0.65, 0.45, 0.8, 1.0, 0.1),
                 FirstExchangeble = TRUE)
head(AFSim[order(AFSim$Dev),], 5)
```

```
##          G    b    M    w    R    r PhenB PhenD PhenG PhenL          Dev
## 7359 0.49 0.51 0.34 0.66 0.95 0.05  0.58  0.38  0.04     0 2.731204e-06
## 7212 0.55 0.45 0.31 0.69 0.95 0.05  0.58  0.38  0.04     0 2.748760e-06
## 7657 0.41 0.59 0.40 0.60 0.95 0.05  0.58  0.38  0.04     0 3.112824e-06
## 6930 0.79 0.21 0.25 0.75 0.95 0.05  0.58  0.38  0.04     0 3.217692e-06
## 7458 0.46 0.54 0.36 0.64 0.95 0.05  0.58  0.38  0.04     0 3.282680e-06
```

```
cat("Combinations within an order of magnitude of best combination: ",
             sum(AFSim$Dev<=min(AFSim$Dev)*10))
```

```
## Combinations within an order of magnitude of best combination:  41
```

A reasonable fit to field morph frequencies can be achieved with allele frequencies of $p_G = 0.49$, $p_b = 0.51$, $p_M = 0.34$, $p_w = 0.66$, $p_R = 0.95$ and $p_r = 0.05$. However, there is a broad range of allele frequencies that fit well, with a negative correlation between p_G and p_M, but the conclusions are qualitatively unaffected.

```
AF = c(G=0.55, b=0.45, M=0.31, w=0.69, R=0.95, r=0.05)
#AF = c(G=0.79, B=0.21, M=0.25, w=0.75, R=0.95, r=0.05) # An alternative
gen$Freq = AF[substr(gen$Genotype,1,1)] * AF[substr(gen$Genotype,2,2)] *
           AF[substr(gen$Genotype,3,3)] * AF[substr(gen$Genotype,4,4)] *
           AF[substr(gen$Genotype,5,5)] * AF[substr(gen$Genotype,6,6)]
if(round(sum(gen$Freq),10)!=1)
  print("Warning: Genotype frequencies do not add to 1")
rbind(Simulated=round(tapply(gen$Freq, gen$Phenotype, sum),2),
      Observed=round(FieldFreq,2))
```

```
##              B    D    G    L
## Simulated 0.58 0.38 0.04 0.00
## Observed  0.58 0.38 0.03 0.02
```

Now parents are sampled from this population according to the implemented mating design. Offspring genotypes are sampled from parental genotypes, offspring phenotypes determined, and the results tabulated. The process is repeated *nruns* times. Simulation results are plotted (dots) along with the observed number of offspring (bars) per mating combination. The following table shows the proportion of simulations that are as extreme as or more extreme than the observed number of offspring (a p value for the probability that the observations arose by sampling variation given the mating design). Only mating combinations in which at least one offspring morph shows $p < 0.05$ are shown.

```
nruns=1000
res = simOligoFnc(gen, dat, FemaleUq, MaleUq, nruns)
pVals = plotFnc(obs, res)
```

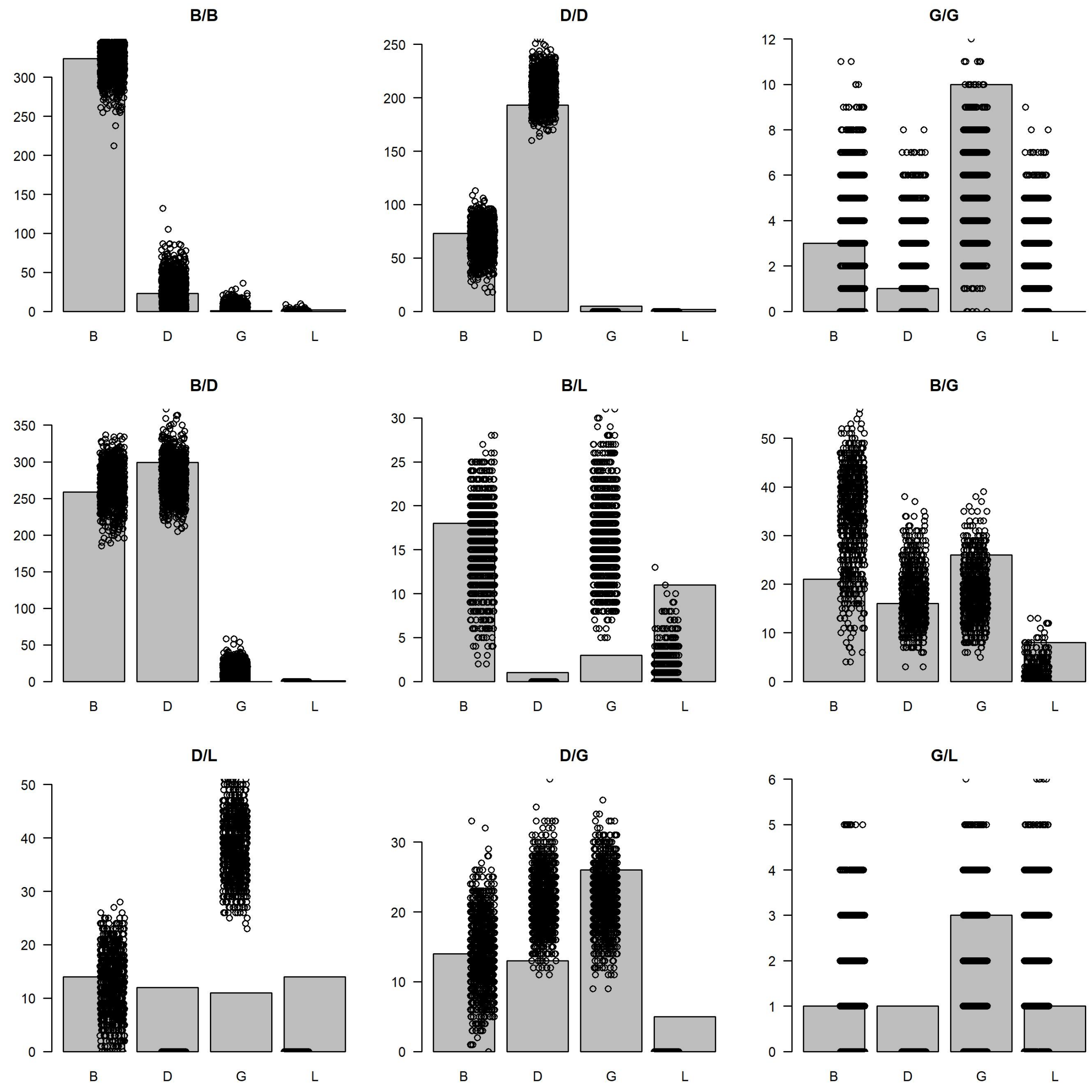


```r
evalTab["Model 9", 6:11] = modevalFnc(obs, res)
```

```
## Model fit in offspring numbers and relative improvement to null (uniform) model
## Absolute deviations:            231 (83.3%)
## Sq root of squared deviations: 55 (87%)
## Sum of squared deviations:      2997 (98.3%)
```

```r
pVals[apply(pVals[,5:8],1,min)<0.05, 5:12]
```

```
##        pB    pD    pG    pL Bsim0 Dsim0 Gsim0 Lsim0
## B/B 0.382 0.380 0.517 0.018 FALSE FALSE FALSE FALSE
## D/D 0.379 0.224 0.000 0.000 FALSE FALSE  TRUE  TRUE
## G/G 0.497 0.239 0.019 0.068 FALSE FALSE FALSE FALSE
## B/D 0.376 0.206 0.038 0.000 FALSE FALSE FALSE  TRUE
## B/L 0.390 0.000 0.000 0.002 FALSE  TRUE FALSE FALSE
```

```
## B/G 0.104 0.483 0.095 0.019 FALSE FALSE FALSE FALSE
## D/L 0.447 0.000 0.000 0.000 FALSE  TRUE FALSE  TRUE
## D/G 0.530 0.015 0.200 0.000 FALSE FALSE FALSE  TRUE
## G/L 0.513 0.000 0.417 0.294 FALSE  TRUE FALSE FALSE
```

The model predicts offspring morph frequencies that are significantly different from observed numbers for most mating combinations. In particular the model does not allow for L offspring from D/D, B/D, D/L and D/G mating combinations, it does not allow for D offspring from B/L, D/L and G/L mating combinations and for G offspring from D/D matings. Several other numbers are significantly over- or underpredicted. These patterns are thus incompatible with the observed data.

Four-locus models

Model 10: Four-locus models with two alleles (2 dominant alleles for L)

There might be four loci involved in color morph determination: One locus G with two alleles (G and b) might be responsible for the ability to produce green color, with G dominant over b. One locus D with two alleles (D and u) where the recessive allele u suppresses green color dorsally. Two loci L and M with two alleles each (L and n and M and w, respectively) where the recessive alleles n and w suppresses green color laterally when either one is in homozygous state.

```
gen = data.frame(Genotype=rep(NA,256), Phenotype=NA, Freq=NA)
i = 1
for(g1 in c("G", "b"))
  for(g2 in c("G", "b"))
    for(d1 in c("D", "u"))
      for(d2 in c("D", "u"))
        for(l1 in c("L", "n"))
          for(l2 in c("L", "n"))
            for(m1 in c("M", "w"))
              for(m2 in c("M", "w")) {
                gen$Genotype[i] = paste0(g1, g2, d1, d2, l1, l2, m1, m2)
                i = i + 1
              }
gen$Phenotype[is.na(gen$Phenotype) &
              (substr(gen$Genotype,1,1)=="b" & substr(gen$Genotype,2,2)=="b")] = "B"
gen$Phenotype[is.na(gen$Phenotype) &
              (substr(gen$Genotype,3,3)=="u" & substr(gen$Genotype,4,4)=="u") &
              ((substr(gen$Genotype,5,5)=="n" & substr(gen$Genotype,6,6)=="n") |
               (substr(gen$Genotype,7,7)=="w" & substr(gen$Genotype,8,8)=="w"))] = "B"
gen$Phenotype[is.na(gen$Phenotype) &
              (substr(gen$Genotype,3,3)=="D" | substr(gen$Genotype,4,4)=="D") &
              (substr(gen$Genotype,5,5)=="L" | substr(gen$Genotype,6,6)=="L") &
              (substr(gen$Genotype,7,7)=="M" | substr(gen$Genotype,8,8)=="M")] = "G"
gen$Phenotype[is.na(gen$Phenotype) &
              (substr(gen$Genotype,3,3)=="D" | substr(gen$Genotype,4,4)=="D") &
              ((substr(gen$Genotype,5,5)=="n" & substr(gen$Genotype,6,6)=="n") |
               (substr(gen$Genotype,7,7)=="w" & substr(gen$Genotype,8,8)=="w"))] = "D"
gen$Phenotype[is.na(gen$Phenotype) &
              (substr(gen$Genotype,5,5)=="L" | substr(gen$Genotype,6,6)=="L") &
              (substr(gen$Genotype,7,7)=="M" | substr(gen$Genotype,8,8)=="M")] = "L"
```

These genotypes would produce phenotypes as follows.

```
PunnettSquare(gen, nloci=4)
```

```
##      GDLM GDLw GDnM GDnw GuLM GuLw GunM Gunw bDLM bDLw bDnM bDnw buLM buLw bunM
## GDLM    G    G    G    G    G    G    G    G    G    G    G    G    G    G    G
## GDLw    G    D    G    D    G    D    G    D    G    D    G    D    G    D    G
## GDnM    G    G    D    D    G    G    D    D    G    G    D    D    G    G    D
## GDnw    G    D    D    D    G    D    D    D    G    D    D    D    G    D    D
## GuLM    G    G    G    G    L    L    L    L    G    G    G    G    L    L    L
## GuLw    G    D    G    D    L    B    L    B    G    D    G    D    L    B    L
## GunM    G    G    D    D    L    L    B    B    G    G    D    D    L    L    B
## Gunw    G    D    D    D    L    B    B    B    G    D    D    D    L    B    B
## bDLM    G    G    G    G    G    G    G    G    B    B    B    B    B    B    B
```

```
## bDLw     G     D     G     D     G     D     G     D     B     B     B     B     B     B     B
## bDnM     G     G     D     D     G     G     D     D     B     B     B     B     B     B     B
## bDnw     G     D     D     D     G     D     D     D     B     B     B     B     B     B     B
## buLM     G     G     G     G     L     L     L     L     B     B     B     B     B     B     B
## buLw     G     D     G     D     L     B     L     B     B     B     B     B     B     B     B
## bunM     G     G     D     D     L     L     B     B     B     B     B     B     B     B     B
## bunw     G     D     D     D     L     B     B     B     B     B     B     B     B     B     B
##       bunw
## GDLM     G
## GDLw     D
## GDnM     D
## GDnw     D
## GuLM     L
## GuLw     B
## GunM     B
## Gunw     B
## bDLM     B
## bDLw     B
## bDnM     B
## bDnw     B
## buLM     B
## buLw     B
## bunM     B
## bunw     B
```

We use simulations to find allele frequencies that fit field morph frequencies. The first round scans in allele frequency steps of 5% to explore the full range of possibilities, the second round scans in steps of 2% and the third in steps of 1% to test for the best combination in a restricted range. The output shows a column *Dev* with the sum of squared deviation between predictions and field morph frequencies, where lower values indicate a better fit (the top 5 results are shown).

```
AFSim = AFsimOligoFnc(gen, AFstep=0.05, nLoci=4, FieldFreq=FieldFreq,
                 allelesL1 = c("G", "b"), allelesL2 = c("D", "u"),
                 allelesL3 = c("L", "n"), allelesL4 = c("M", "w"),
                 LastExchangeble = TRUE)
head(AFSim[order(AFSim$Dev),], 5)
```

```
##          G    b    D    u    L    n    M    w PhenB PhenD PhenG PhenL
## 2650  0.45 0.55 0.35 0.65 0.40 0.60 0.05 0.95  0.58  0.38  0.03  0.02
## 9315  0.25 0.75 0.80 0.20 0.35 0.65 0.10 0.90  0.58  0.37  0.05  0.00
## 15091 0.25 0.75 0.80 0.20 0.20 0.80 0.15 0.85  0.58  0.38  0.04  0.00
## 3408  0.35 0.65 0.45 0.55 0.50 0.50 0.05 0.95  0.58  0.37  0.03  0.01
## 8101  0.35 0.65 0.45 0.55 0.20 0.80 0.10 0.90  0.59  0.38  0.03  0.01
##                Dev
## 2650  9.553691e-06
## 9315  1.312520e-05
## 15091 1.409835e-05
## 3408  1.595599e-05
## 8101  1.648208e-05
```

```
AFSim = AFsimOligoFnc(gen, AFstep=0.02, nLoci=4, FieldFreq=FieldFreq,
                 allelesL1 = c("G", "b"), allelesL2 = c("D", "u"),
                 allelesL3 = c("L", "n"), allelesL4 = c("M", "w"),
                 AFmax = c(0.48, 0.68, 0.48, 0.68, 0.28, 0.88, 0.18, 0.94),
                 LastExchangeble = TRUE)
head(AFSim[order(AFSim$Dev),], 5)
```

```
##         G    b    D    u    L    n    M    w PhenB PhenD PhenG PhenL
## 336  0.46 0.54 0.34 0.66 0.22 0.78 0.08 0.92  0.58  0.38  0.02  0.02
## 1395 0.36 0.64 0.44 0.56 0.18 0.82 0.12 0.88  0.58  0.38  0.03  0.01
## 563  0.36 0.64 0.44 0.56 0.28 0.72 0.08 0.92  0.58  0.38  0.03  0.01
## 947  0.36 0.64 0.44 0.56 0.22 0.78 0.10 0.90  0.58  0.38  0.03  0.01
## 784  0.46 0.54 0.34 0.66 0.18 0.82 0.10 0.90  0.58  0.37  0.02  0.02
##               Dev
## 336  1.652866e-07
## 1395 2.101232e-07
## 563  2.383297e-07
## 947  4.614024e-07
## 784  6.972655e-07
```

```
AFSim = AFsimOligoFnc(gen, AFstep=0.01, nLoci=4, FieldFreq=FieldFreq,
                 allelesL1 = c("G", "b"), allelesL2 = c("D", "u"),
                 allelesL3 = c("L", "n"), allelesL4 = c("M", "w"),
                 AFmax = c(0.47, 0.65, 0.45, 0.67, 0.23, 0.83, 0.13, 0.93),
                 LastExchangeble = TRUE)
head(AFSim[order(AFSim$Dev),], 5)
```

```
##         G    b    D    u    L    n    M    w PhenB PhenD PhenG PhenL
## 1584 0.46 0.54 0.34 0.66 0.22 0.78 0.08 0.92  0.58  0.38  0.02  0.02
## 4814 0.36 0.64 0.44 0.56 0.18 0.82 0.12 0.88  0.58  0.38  0.03  0.01
## 4034 0.36 0.64 0.44 0.56 0.19 0.81 0.11 0.89  0.58  0.38  0.03  0.01
## 3410 0.36 0.64 0.44 0.56 0.21 0.79 0.10 0.90  0.58  0.38  0.03  0.01
## 2208 0.46 0.54 0.34 0.66 0.20 0.80 0.09 0.91  0.58  0.38  0.02  0.02
##               Dev
## 1584 1.652866e-07
## 4814 2.101232e-07
## 4034 3.835210e-07
## 3410 4.263810e-07
## 2208 4.465664e-07
```

```
cat("Combinations within an order of magnitude of best combination: ",
             sum(AFSim$Dev<=min(AFSim$Dev)*10))
```

```
## Combinations within an order of magnitude of best combination:  11
```

A reasonable fit to field morph frequencies can be achieved with allele frequencies of $p_G = 0.46$, $p_b = 0.54$, $p_D = 0.34$, $p_u = 0.66$, $p_L = 0.22$, $p_n = 0.78$, $p_M = 0.08$ and $p_n = 0.92$.

```
AF = c(G=0.46, b=0.54, D=0.34, u=0.66, L=0.22, n=0.78, M=0.08, w=0.92)
# An Alternative
#AF = c(G=0.36, b=0.64, D=0.44, u=0.56, L=0.18, n=0.82, M=0.12, w=0.88)
gen$Freq = AF[substr(gen$Genotype,1,1)] * AF[substr(gen$Genotype,2,2)] *
           AF[substr(gen$Genotype,3,3)] * AF[substr(gen$Genotype,4,4)] *
           AF[substr(gen$Genotype,5,5)] * AF[substr(gen$Genotype,6,6)] *
           AF[substr(gen$Genotype,7,7)] * AF[substr(gen$Genotype,8,8)]
if(round(sum(gen$Freq),10)!=1)
  print("Warning: Genotype frequencies do not add to 1")
rbind(Simulated=round(tapply(gen$Freq, gen$Phenotype, sum),2),
      Observed=round(FieldFreq,2))
```

```
##              B    D    G    L
## Simulated 0.58 0.38 0.02 0.02
```

```
## Observed  0.58 0.38 0.03 0.02
```

Now parents are sampled from this population according to the implemented mating design. Offspring genotypes are sampled from parental genotypes, offspring phenotypes determined, and the results tabulated. The process is repeated *nruns* times. Simulation results are plotted (dots) along with the observed number of offspring (bars) per mating combination. The following table shows the proportion of simulations that are as extreme as or more extreme than the observed number of offspring (a p value for the probability that the observations arose by sampling variation given the mating design). Only mating combinations in which at least one offspring morph shows $p < 0.05$ are shown.

```
nruns=1000
res = simOligoFnc(gen, dat, FemaleUq, MaleUq, nruns)
pVals = plotFnc(obs, res)
```

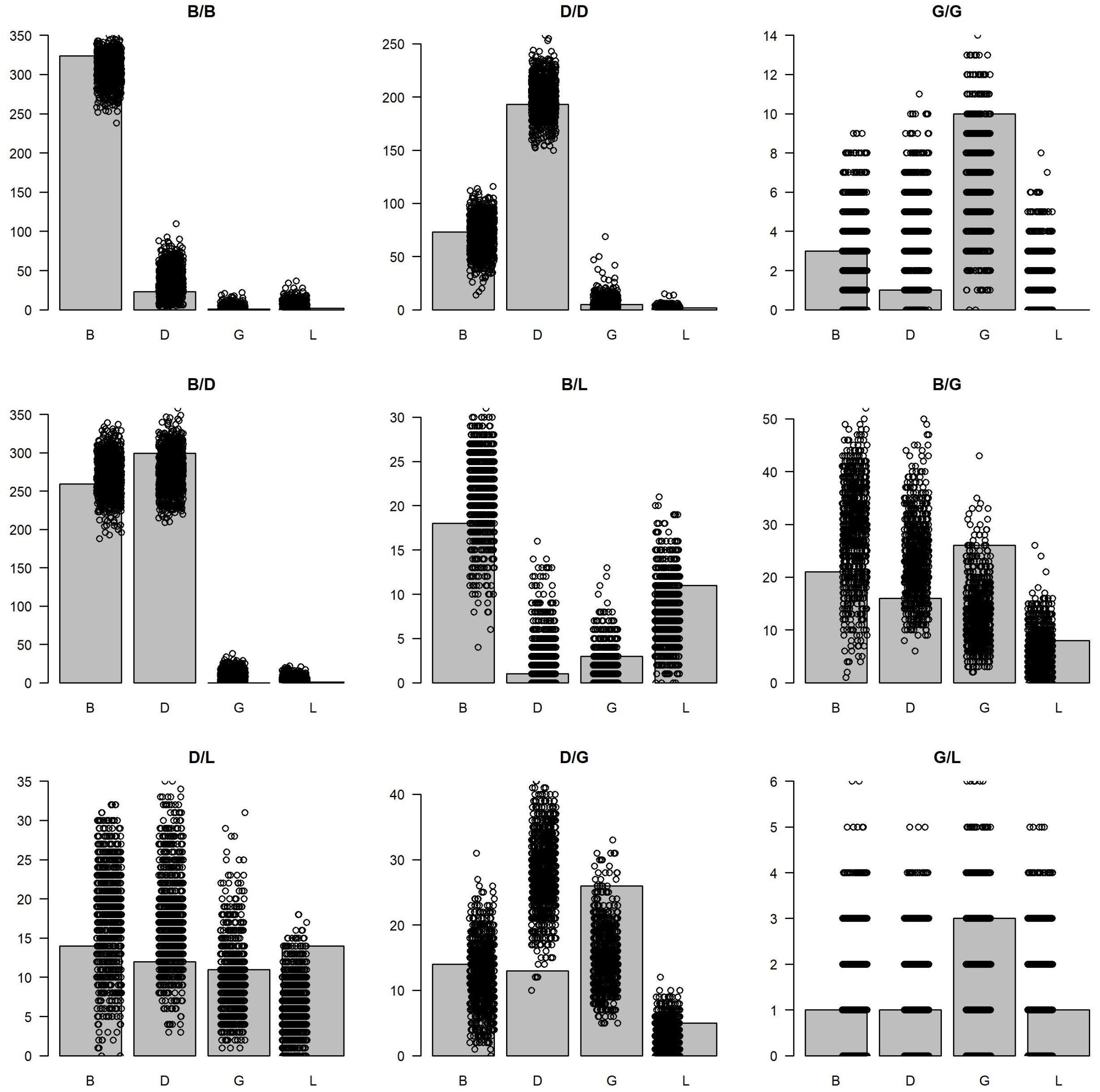

```
evalTab["Model 10", 6:11] = modevalFnc(obs, res)
```

```
## Model fit in offspring numbers and relative improvement to null (uniform) model
## Absolute deviations:           189 (86.3%)
## Sq root of squared deviations: 47 (88.9%)
## Sum of squared deviations:     2203 (98.8%)
```

```
pVals[apply(pVals[,5:8],1,min)<0.05, 5:12]
```

```
##        pB    pD    pG    pL Bsim0 Dsim0 Gsim0 Lsim0
## B/D 0.288 0.165 0.038 0.290 FALSE FALSE FALSE FALSE
## B/G 0.212 0.173 0.029 0.371 FALSE FALSE FALSE FALSE
## D/L 0.274 0.200 0.349 0.031 FALSE FALSE FALSE FALSE
## D/G 0.393 0.004 0.025 0.218 FALSE FALSE FALSE FALSE
```

The model predicts offspring morph frequencies that appear to be an overall good fit to the observed numbers. The number of G offspring is slighly underpredicted for B/G matings and the number of L offspring is slightly underpredicted for D/L matings. The biggest misprediction occurs for D/G matings where D offspring are over and G offspring underpredicted. Notably, there is no observed offspring that is impossibly produced by the model.

Threshold-based multi-locus models

Model 11: One-trait model with morph genotypic values (D > L > G > B)

There might also be many loci influencing a single trait with trait-value thresholds determining morph identities. We here assume five independent loci (or haplotypes) with equal effect sizes to allow for some sampling variation (though there could be many more loci involved). First, we generate a large pool of individuals (controlled by *nind*), each with 10 alleles (2 at 5 loci). Genotypic values (*GV1*) are calculated as the average across alleles. The simulation is set up for more traits to be generated later, though genotypic values for (silent) traits 2 and 3 (*GV2* and *GV3*) are constraint to zero here.

```
nind=100000
loci = data.frame(matrix(c(rnorm(nind*10,0,sqrt(10)), rep(0,nind*20)), ncol=30))
gen = data.frame(loci, GV1=rowMeans(loci[,1:10]), GV2=0, GV3=0)
gen$Phenotype = NA
phenFnc = function(mydat, ThreshT1, ThreshT2, ThreshT3) {
  Phenotype = NA
  Phenotype[is.na(Phenotype) & mydat$GV1 < ThreshT1["B"]] = "B"
  Phenotype[is.na(Phenotype) & mydat$GV1 < ThreshT1["G"]] = "G"
  Phenotype[is.na(Phenotype) & mydat$GV1 < ThreshT1["L"]] = "L"
  Phenotype[is.na(Phenotype)] = "D"
  return(Phenotype)
}
```

We use simulations to find genotypic thresholds that fit field morph frequencies. The first round scans in allele frequency steps of 5% to explore the full range of possibilities, the second round scans in steps of 2% and the third in steps of 1% to test for the best combination in a restricted range. The output shows a column *Dev* with the sum of squared deviation between predictions and field morph frequencies, where lower values indicate a better fit (the top 10 results are shown).

```
ThSim = ThreshSimQuantFnc(gen, quantSteps=0.05, nTraits=1, FieldFreq=FieldFreq,
                  ThNamesT1 = c("B", "G", "L"), phenFnc=phenFnc)
head(ThSim[order(ThSim$Dev),], 10)
```

```
##              B         G         L PhenB PhenD PhenG PhenL         Dev
## 4552 0.1256613 0.2533471 0.3853205  0.55  0.35  0.05  0.05 0.001717667
## 4933 0.2533471 0.3853205 0.5244005  0.60  0.30  0.05  0.05 0.006111758
## 4913 0.1256613 0.2533471 0.5244005  0.55  0.30  0.05  0.10 0.006820897
## 4932 0.1256613 0.3853205 0.5244005  0.55  0.30  0.10  0.05 0.006820897
## 4171 0.0000000 0.1256613 0.2533471  0.50  0.40  0.05  0.05 0.007274847
## 4532 0.0000000 0.1256613 0.3853205  0.50  0.35  0.05  0.10 0.007339901
## 4551 0.0000000 0.2533471 0.3853205  0.50  0.35  0.10  0.05 0.007339901
## 4893 0.0000000 0.1256613 0.5244005  0.50  0.30  0.05  0.15 0.012443131
## 4912 0.0000000 0.2533471 0.5244005  0.50  0.30  0.10  0.10 0.012443131
## 4931 0.0000000 0.3853205 0.5244005  0.50  0.30  0.15  0.05 0.012443131
```

```
ThSim = ThreshSimQuantFnc(gen, quantSteps=0.02, nTraits=1, FieldFreq=FieldFreq,
                  ThNamesT1 = c("B", "G", "L"), phenFnc=phenFnc,
                  minT=c(-0.04, 0.04, 0.24), maxT=c(0.3, 0.48, 0.6))
head(ThSim[order(ThSim$Dev),], 10)
```

```
##               B         G         L PhenB PhenD PhenG PhenL          Dev
## 73480 0.2018935 0.2533471 0.3054808  0.58  0.38  0.02  0.02 2.683952e-05
## 75881 0.2018935 0.2533471 0.3584588  0.58  0.36  0.02  0.04 2.454123e-04
## 75930 0.2018935 0.3054808 0.3584588  0.58  0.36  0.04  0.02 2.454123e-04
## 73430 0.1509692 0.2018935 0.3054808  0.56  0.38  0.02  0.04 5.200724e-04
```

```
## 73479 0.1509692 0.2533471 0.3054808  0.56  0.38  0.04  0.02 5.200724e-04
## 75931 0.2533471 0.3054808 0.3584588  0.60  0.36  0.02  0.02 5.898713e-04
## 75831 0.1509692 0.2018935 0.3584588  0.56  0.36  0.02  0.06 7.386452e-04
## 75880 0.1509692 0.2533471 0.3584588  0.56  0.36  0.04  0.04 7.386452e-04
## 75929 0.1509692 0.3054808 0.3584588  0.56  0.36  0.06  0.02 7.386452e-04
## 71029 0.1509692 0.2018935 0.2533471  0.56  0.40  0.02  0.02 1.092248e-03
```

```
ThSim = ThreshSimQuantFnc(gen, quantSteps=0.01, nTraits=1, FieldFreq=FieldFreq,
                 ThNamesT1 = c("B", "G", "L"), phenFnc=phenFnc,
                 minT=c(-0.05, 0.05, 0.25), maxT=c(0.3, 0.45, 0.6))
head(ThSim[order(ThSim$Dev),], 10)
```

```
##                B         G         L PhenB PhenD PhenG PhenL          Dev
## 603661 0.2018935 0.2275450 0.3054808  0.58  0.38  0.01  0.03 2.683952e-05
## 603760 0.2018935 0.2533471 0.3054808  0.58  0.38  0.02  0.02 2.683952e-05
## 603859 0.2018935 0.2793190 0.3054808  0.58  0.38  0.03  0.01 2.683952e-05
## 613462 0.2018935 0.2275450 0.3318533  0.58  0.37  0.01  0.04 3.574438e-05
## 613561 0.2018935 0.2533471 0.3318533  0.58  0.37  0.02  0.03 3.574438e-05
## 613660 0.2018935 0.2793190 0.3318533  0.58  0.37  0.03  0.02 3.574438e-05
## 613759 0.2018935 0.3054808 0.3318533  0.58  0.37  0.04  0.01 3.574438e-05
## 603761 0.2275450 0.2533471 0.3054808  0.59  0.38  0.01  0.02 9.770353e-05
## 603860 0.2275450 0.2793190 0.3054808  0.59  0.38  0.02  0.01 9.770353e-05
## 613562 0.2275450 0.2533471 0.3318533  0.59  0.37  0.01  0.03 1.066084e-04
```

```
print(paste0("Within one order of magnitude: of best combination:  ", length(ThSim$Dev<=min(ThSim$Dev)*
```

```
## [1] "Within one order of magnitude: of best combination:  1525"
```

A reasonable fit to field morph frequencies can be achieved with thresholds of $T_B = 0.20$, $T_G = 0.23$, $T_L = 0.33$. Note slight sampling variation between runs.

```
ThreshT1 = c(B=0.20, G=0.23, L=0.33)
gen$Phenotype = phenFnc(gen, ThreshT1, NA, NA)
hist(gen$GV1, nclass=30, main="Single color-morph trait")
abline(v=ThreshT1, lwd=2)
text(c(-3,-1,1,3), 18000, c("Uniform\nbrown", "Uniform\ngreen",
                              "Lateral\ngreen", "Dorsal\ngreen"))
```

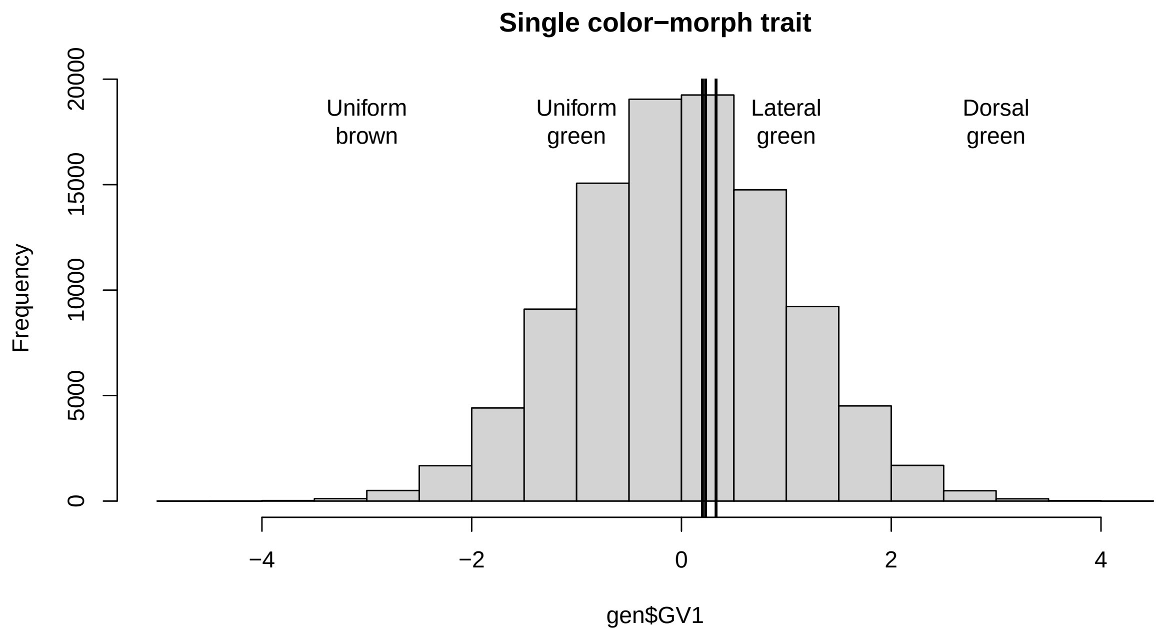


```
print("Field morph frequencies")
```

```
## [1] "Field morph frequencies"
```

```
rbind(Simulated=round(table(gen$Phenotype)/nrow(gen),2),
      Observed=round(FieldFreq,2))
```

```
##              B    D    G    L
## Simulated 0.58 0.37 0.01 0.04
## Observed  0.58 0.38 0.03 0.02
```

Now parents are sampled from this population according to the implemented mating design. Offspring genotypic values are sampled from parental genotypes, offspring phenotype determined and the results tabulated. The process is repeated *nruns* times. Simulation results are plotted (dots) along with the observed number of offspring (bars) per mating combination. The following table shows the proportion of simulations that are as extreme as or more extreme than the observed number of offspring (a p value for the probability that the observations arose by sampling variation given the mating design). Only mating combinations in which at least one offspring morph shows $p < 0.05$ are shown.

```
nruns=1000
res = simQuantFnc(gen, dat, FemaleUq, MaleUq, nruns, phenFnc, ThreshT1, NA, NA)
pVals = plotFnc(obs, res)
```

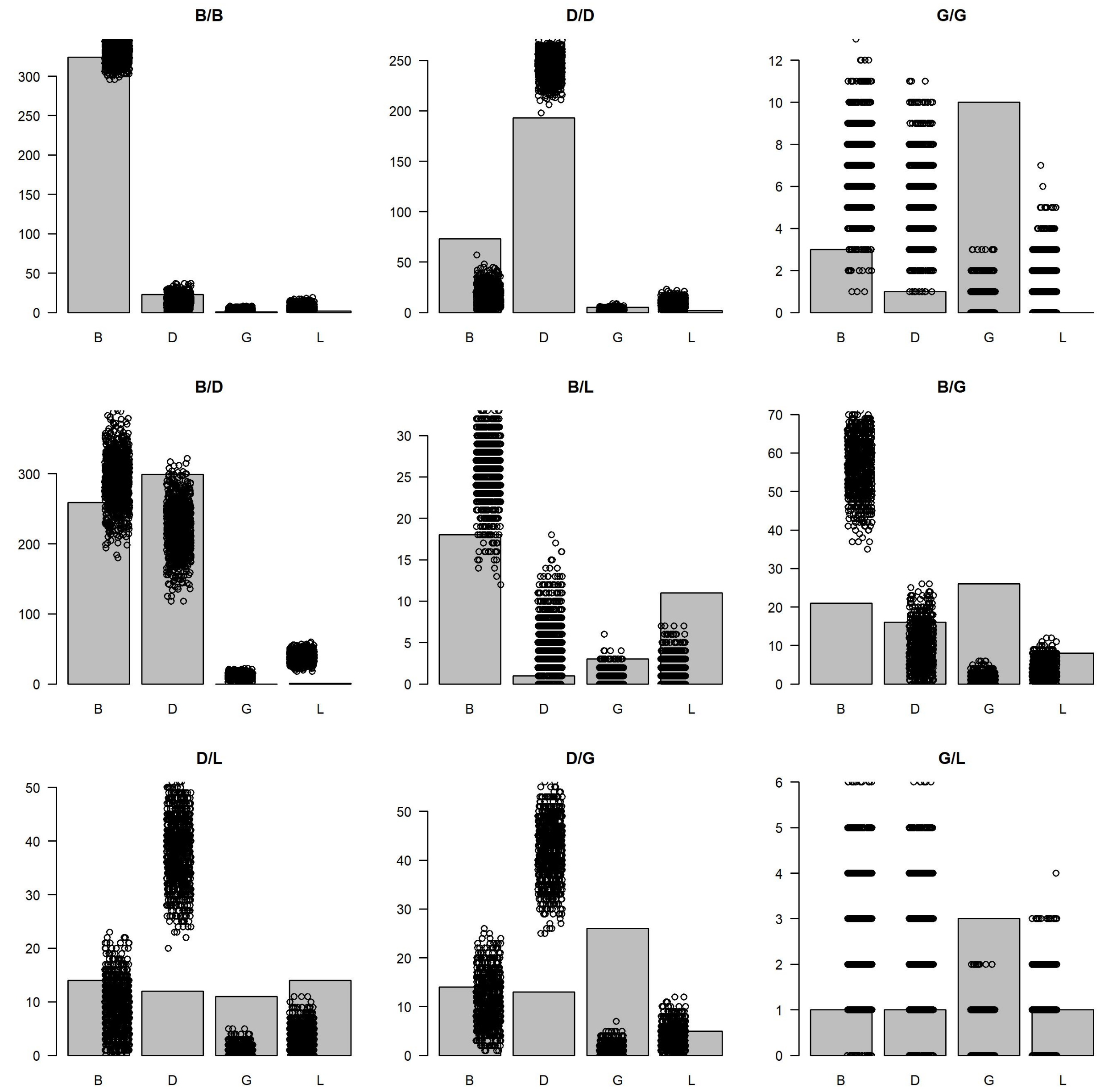


```
evalTab["Model 11", 6:11] = modevalFnc(obs, res)
```

```
## Model fit in offspring numbers and relative improvement to null (uniform) model
## Absolute deviations:           526 (61.9%)
## Sq root of squared deviations: 140 (66.9%)
## Sum of squared deviations:     19471 (89.1%)
```

```
pVals[apply(pVals[,5:8],1,min)<0.05, 5:12]
```

```
##        pB    pD    pG    pL Bsim0 Dsim0 Gsim0 Lsim0
## D/D 0.000 0.000 0.063 0.047 FALSE FALSE FALSE FALSE
## G/G 0.034 0.009 0.000 0.249 FALSE FALSE FALSE FALSE
## B/D 0.208 0.014 0.000 0.000 FALSE FALSE FALSE FALSE
## B/L 0.040 0.120 0.031 0.000 FALSE FALSE FALSE FALSE
## B/G 0.000 0.130 0.000 0.057 FALSE FALSE FALSE FALSE
```

```
## D/L 0.153 0.000 0.000 0.000 FALSE FALSE FALSE FALSE
## D/G 0.319 0.000 0.000 0.374 FALSE FALSE FALSE FALSE
## G/L 0.156 0.229 0.000 0.462 FALSE FALSE FALSE FALSE
```

The model gives a poor fit to most mating combinations. For example, the model overpredicts the number of B offspring from B/G matings (but underpredicts B form D/D mating) and it overpredicts the number of D offspring for D/D, D/L and D/G matings and. Furthermore, it underpredicts G offspring for G/G, B/G, D/L, D/G and G/L matings and underpredicts L for B/L, B/G and D/L matings (but overpredicts L for B/D matings). The model is, therefore, incompatible with the observed data.

Model 12: One-trait model with morph genotypic values (G > L > D > B)

There might also be many loci influencing a single trait with trait-value thresholds determining morph identities. We here assume five independent loci (or haplotypes) with equal effect sizes to allow for some sampling variation (though there could be many more loci involved). First, we generate a large pool of individuals (controlled by *nind*), each with 10 alleles (2 at 5 loci). Genotypic values (*GV1*) are calculated as the average across alleles. The simulation is set up for more traits to be generated later, though genotypic values for (silent) traits 2 and 3 (*GV2* and *GV3*) are constraint to zero here.

```
nind=100000
loci = data.frame(matrix(c(rnorm(nind*10,0,sqrt(10)), rep(0,nind*20)), ncol=30))
gen = data.frame(loci, GV1=rowMeans(loci[,1:10]), GV2=0, GV3=0)
gen$Phenotype = NA
phenFnc = function(mydat, ThreshT1, ThreshT2, ThreshT3) {
  Phenotype = NA
  Phenotype[is.na(Phenotype) & mydat$GV1 < ThreshT1["B"]] = "B"
  Phenotype[is.na(Phenotype) & mydat$GV1 < ThreshT1["D"]] = "D"
  Phenotype[is.na(Phenotype) & mydat$GV1 < ThreshT1["L"]] = "L"
  Phenotype[is.na(Phenotype)] = "G"
  return(Phenotype)
}
```

We use simulations to find genotypic thresholds that fit field morph frequencies. The first round scans in allele frequency steps of 5% to explore the full range of possibilities, the second round scans in steps of 2% and the third in steps of 1% to test for the best combination in a restricted range. The output shows a column *Dev* with the sum of squared deviation between predictions and field morph frequencies, where lower values indicate a better fit (the top 10 results are shown).

```
ThSim = ThreshSimQuantFnc(gen, quantSteps=0.05, nTraits=1, FieldFreq=FieldFreq,
                  ThNamesT1 = c("B", "D", "L"), phenFnc=phenFnc)
head(ThSim[order(ThSim$Dev),], 10)
```

```
##              B         D        L PhenB PhenD PhenG PhenL         Dev
## 6832 0.1256613 1.2815516 1.644854  0.55  0.35  0.05  0.05 0.001699222
## 6833 0.2533471 1.2815516 1.644854  0.60  0.30  0.05  0.05 0.005995136
## 6452 0.1256613 1.0364334 1.281552  0.55  0.30  0.10  0.05 0.006940416
## 6813 0.1256613 1.0364334 1.644854  0.55  0.30  0.05  0.10 0.006940416
## 6831 0.0000000 1.2815516 1.644854  0.50  0.40  0.05  0.05 0.007379502
## 6451 0.0000000 1.0364334 1.281552  0.50  0.35  0.10  0.05 0.007501013
## 6812 0.0000000 1.0364334 1.644854  0.50  0.35  0.05  0.10 0.007501013
## 6071 0.0000000 0.8416212 1.036433  0.50  0.30  0.15  0.05 0.012611859
## 6432 0.0000000 0.8416212 1.281552  0.50  0.30  0.10  0.10 0.012611859
## 6793 0.0000000 0.8416212 1.644854  0.50  0.30  0.05  0.15 0.012611859
```

```
ThSim = ThreshSimQuantFnc(gen, quantSteps=0.02, nTraits=1, FieldFreq=FieldFreq,
                  ThNamesT1 = c("B", "D", "L"), phenFnc=phenFnc,
                  minT=c(-0.04, 0.8, 1.0), maxT=c(0.3, 1.4, 1.8))
head(ThSim[order(ThSim$Dev),], 10)
```

```
##                B        D        L PhenB PhenD PhenG PhenL         Dev
## 110229 0.1509692 1.281552 1.405072  0.56  0.34  0.08  0.02 0.001761431
## 112630 0.1509692 1.281552 1.554774  0.56  0.34  0.06  0.04 0.001761431
## 115031 0.1509692 1.281552 1.750686  0.56  0.34  0.04  0.06 0.001761431
## 110228 0.1004337 1.281552 1.405072  0.54  0.36  0.08  0.02 0.002037171
## 112629 0.1004337 1.281552 1.554774  0.54  0.36  0.06  0.04 0.002037171
## 115030 0.1004337 1.281552 1.750686  0.54  0.36  0.04  0.06 0.002037171
```

```
## 110230 0.2018935 1.281552 1.405072  0.58  0.32  0.08  0.02 0.003075655
## 112631 0.2018935 1.281552 1.554774  0.58  0.32  0.06  0.04 0.003075655
## 115032 0.2018935 1.281552 1.750686  0.58  0.32  0.04  0.06 0.003075655
## 107778 0.1004337 1.174987 1.281552  0.54  0.34  0.10  0.02 0.003119643
```

```
ThSim = ThreshSimQuantFnc(gen, quantSteps=0.01, nTraits=1, FieldFreq=FieldFreq,
                 ThNamesT1 = c("B", "D", "L"), phenFnc=phenFnc,
                 minT=c(-0.05, 0.8, 1.0), maxT=c(0.3, 1.4, 1.8))
head(ThSim[order(ThSim$Dev),], 10)
```

```
##                  B        D        L PhenB PhenD PhenG PhenL         Dev
## 900857 0.1509692 1.340755 1.405072  0.56  0.35  0.08  0.01 0.001128980
## 910658 0.1509692 1.340755 1.475791  0.56  0.35  0.07  0.02 0.001128980
## 920459 0.1509692 1.340755 1.554774  0.56  0.35  0.06  0.03 0.001128980
## 930260 0.1509692 1.340755 1.644854  0.56  0.35  0.05  0.04 0.001128980
## 940061 0.1509692 1.340755 1.750686  0.56  0.35  0.04  0.05 0.001128980
## 900856 0.1256613 1.340755 1.405072  0.55  0.36  0.08  0.01 0.001278083
## 910657 0.1256613 1.340755 1.475791  0.55  0.36  0.07  0.02 0.001278083
## 920458 0.1256613 1.340755 1.554774  0.55  0.36  0.06  0.03 0.001278083
## 930259 0.1256613 1.340755 1.644854  0.55  0.36  0.05  0.04 0.001278083
## 940060 0.1256613 1.340755 1.750686  0.55  0.36  0.04  0.05 0.001278083
```

```
print(paste0("Within one order of magnitude: of best combination:  ", length(ThSim$Dev<=min(ThSim$Dev)*
```

```
## [1] "Within one order of magnitude: of best combination:  1664"
```

A reasonable fit to field morph frequencies can be achieved with thresholds of $T_B = 0.15$, $T_D = 1.34$, $p_L = 1.41$ (though these thesholds overpredict the number of G morphs). Also note slight sampling variation between runs.

```
ThreshT1 = c(B=0.15, D=1.34, L=1.41)
gen$Phenotype = phenFnc(gen, ThreshT1, NA, NA)
hist(gen$GV1, nclass=30, main="Single color-morph trait")
abline(v=ThreshT1, lwd=2)
text(c(-3,-0.1,1,3), 18000, c("Uniform\nbrown", "Dorsal\ngreen",
                                "Lateral\ngreen", "Uniform\ngreen"))
```

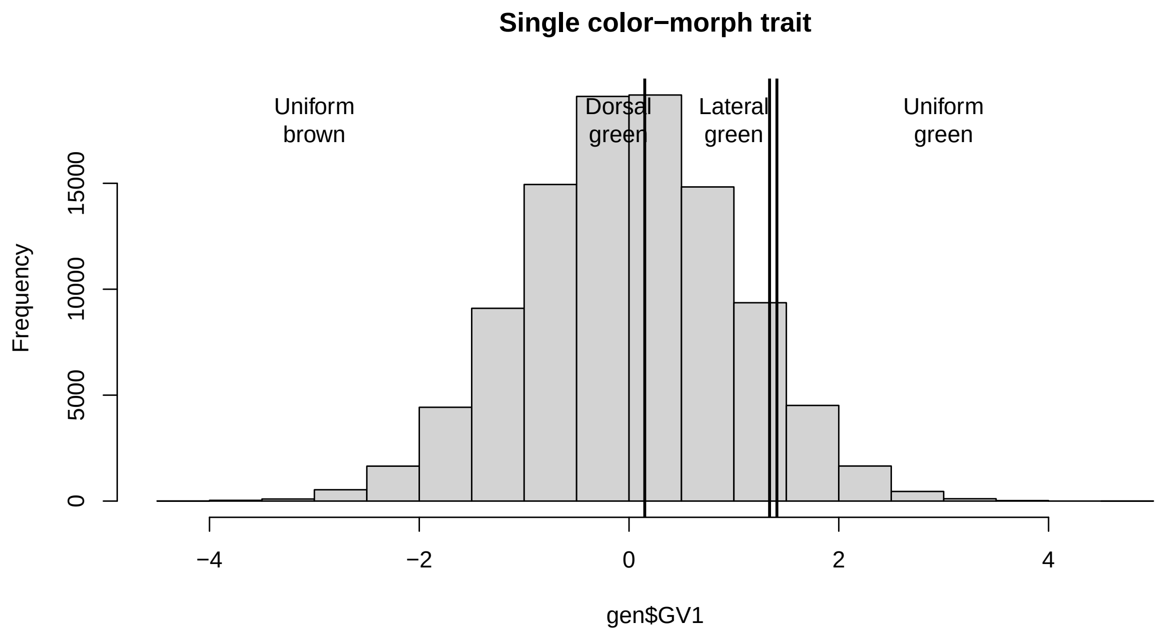


```
print("Field morph frequencies")
```

```
## [1] "Field morph frequencies"
```

```
rbind(Simulated=round(table(gen$Phenotype)/nrow(gen),2),
      Observed=round(FieldFreq,2))
```

```
##              B    D    G    L
## Simulated 0.56 0.35 0.08 0.01
## Observed  0.58 0.38 0.03 0.02
```

Now parents are sampled from this population according to the implemented mating design. Offspring genotypic values are sampled from parental genotypes, offspring phenotype determined and the results tabulated. The process is repeated *nruns* times. Simulation results are plotted (dots) along with the observed number of offspring (bars) per mating combination. The following table shows the proportion of simulations that are as extreme as or more extreme than the observed number of offspring (a p value for the probability that the observations arose by sampling variation given the mating design). Only mating combinations in which at least one offspring morph shows $p < 0.05$ are shown.

```
nruns=1000
res = simQuantFnc(gen, dat, FemaleUq, MaleUq, nruns, phenFnc, ThreshT1, NA, NA)
pVals = plotFnc(obs, res)
```

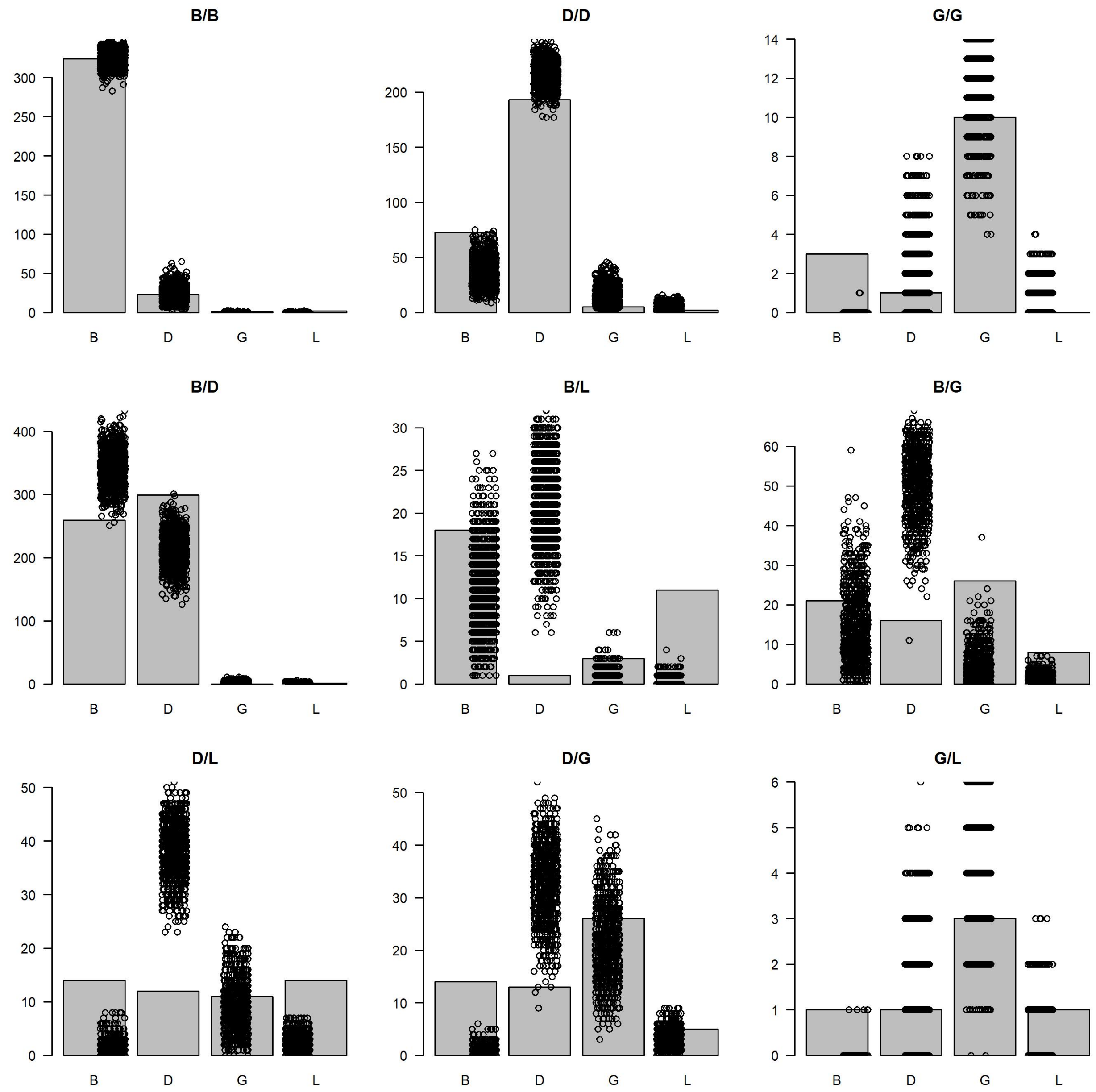


```
evalTab["Model 12", 6:11] = modevalFnc(obs, res)
```

```
## Model fit in offspring numbers and relative improvement to null (uniform) model
## Absolute deviations:           470 (66%)
## Sq root of squared deviations: 144 (65.8%)
## Sum of squared deviations:     20816 (88.3%)
```

```
pVals[apply(pVals[,5:8],1,min)<0.05, 5:12]
```

```
##        pB    pD    pG    pL Bsim0 Dsim0 Gsim0 Lsim0
## B/B 0.416 0.506 0.029 0.001 FALSE FALSE FALSE FALSE
## D/D 0.002 0.022 0.055 0.175 FALSE FALSE FALSE FALSE
## G/G 0.000 0.498 0.282 0.632 FALSE FALSE FALSE FALSE
## B/D 0.002 0.001 0.247 0.576 FALSE FALSE FALSE FALSE
## B/L 0.115 0.000 0.030 0.000 FALSE FALSE FALSE FALSE
```

```
## B/G 0.247 0.001 0.001 0.000 FALSE FALSE FALSE FALSE
## D/L 0.000 0.000 0.386 0.000 FALSE FALSE FALSE FALSE
## D/G 0.000 0.004 0.259 0.242 FALSE FALSE FALSE FALSE
## G/L 0.005 0.492 0.345 0.296 FALSE FALSE FALSE FALSE
```

The model gives a poor fit for most mating combinations. For example, the model underpredicts the number of B offspring for D/D, G/G, D/L and D/G matings (but underpredicts B offspring for B/D matings) and it overpredicts the number of D offspring for D/D, B/L, B/G, D/L and D/G matings (but underpredicts D offspring for B/D matings). Furthermore, it underpredicts G offspring for B/L and B/G matings and underpredicts L for B/L, B/G and D/L matings. The model is, therefore, incompatible with the observed data.

Model 13: Two-trait models

Instead of being explained by a single 'trait', morph could be considered to consist of two 'traits'. Hence, there could be many loci influencing two traits with the combination of three trait-value thresholds determining morph identities. These traits could be green on the dorsal side and green on the lateral side. We here assume five independent loci (or haplotypes) with equal effect sizes per trait to allow for some sampling variation (though there could be many more loci involved). First, we generate a large pool of individuals (controlled by *nind*), each with 20 alleles (2 at 5 loci, for 2 traits). Genotypic values (*GV1* and *GV2*) are calculated as the average across alleles. The simulation is set up for more traits to be generated later, though genotypic values for (silent) trait 3 (*GV3*) are constraint to zero here.

```
nind=100000
loci = data.frame(matrix(c(rnorm(nind*20,0, sqrt(10)), rep(0,nind*10)), ncol=30))
gen = data.frame(loci, GV1=scale(rowMeans(loci[,1:10])),
                       GV2=scale(rowMeans(loci[,11:20])), GV3=0)
gen$Phenotype = NA
phenFnc = function(mydat, ThreshT1, ThreshT2, ThreshT3) {
  Phenotype = NA
  Phenotype[is.na(Phenotype) & mydat$GV1 < ThreshT1 & mydat$GV2 < ThreshT2] = "B"
  Phenotype[is.na(Phenotype) & mydat$GV1 >= ThreshT1 & mydat$GV2 >= ThreshT2] = "G"
  Phenotype[is.na(Phenotype) & mydat$GV1 >= ThreshT1 & mydat$GV2 < ThreshT2] = "D"
  Phenotype[is.na(Phenotype) & mydat$GV1 < ThreshT1 & mydat$GV2 >= ThreshT2] = "L"
  return(Phenotype)
}
```

We use simulations to find genotypic thresholds that fit field morph frequencies. The first round scans in allele frequency steps of 5% to explore the full range of possibilities, the second round scans in steps of 2% and the third in steps of 1% to test for the best combination in a restricted range. The output shows a column *Dev* with the sum of squared deviation between predictions and field morph frequencies, where lower values indicate a better fit (the top 10 results are shown).

```
ThSim = ThreshSimQuantFnc(gen, quantSteps=0.05, nTraits=2, FieldFreq=FieldFreq,
                ThNamesT1 = "T1", ThNamesT2 = "T2", phenFnc=phenFnc)
head(ThSim[order(ThSim$Dev),], 10)
```

```
##            T1        T2 PhenB PhenD PhenG PhenL          Dev
## 354 0.2533471 1.6448536  0.57  0.38  0.02  0.03 0.0001604955
## 335 0.2533471 1.2815516  0.54  0.36  0.04  0.06 0.0020089001
## 355 0.3853205 1.6448536  0.62  0.33  0.02  0.03 0.0032886822
## 336 0.3853205 1.2815516  0.59  0.31  0.04  0.06 0.0038355349
## 353 0.1256613 1.6448536  0.52  0.43  0.02  0.03 0.0062770434
## 316 0.2533471 1.0364334  0.51  0.34  0.06  0.09 0.0064108056
## 317 0.3853205 1.0364334  0.55  0.30  0.05  0.10 0.0070417042
## 334 0.1256613 1.2815516  0.49  0.41  0.05  0.05 0.0084465898
## 298 0.3853205 0.8416212  0.52  0.28  0.07  0.13 0.0130400008
## 315 0.1256613 1.0364334  0.47  0.38  0.07  0.08 0.0131995803
```

```
ThSim = ThreshSimQuantFnc(gen, quantSteps=0.02, nTraits=2, FieldFreq=FieldFreq,
                ThNamesT1 = "T1", ThNamesT2 = "T2", phenFnc=phenFnc,
                min=c(0.04, 1.0), maxT=c(0.48, 1.8))
head(ThSim[order(ThSim$Dev),], 10)
```

```
##             T1       T2 PhenB PhenD PhenG PhenL          Dev
## 2333 0.2533471 1.750686  0.58  0.39  0.02  0.02 0.0001177810
## 2334 0.3054808 1.750686  0.60  0.37  0.02  0.02 0.0003119407
## 2284 0.2533471 1.554774  0.56  0.38  0.02  0.04 0.0003171224
```

```
## 2285 0.3054808 1.554774  0.58  0.36  0.02  0.04 0.0003455407
## 2236 0.3054808 1.405072  0.57  0.35  0.03  0.05 0.0007993388
## 2235 0.2533471 1.405072  0.55  0.37  0.03  0.05 0.0009568361
## 2332 0.2018935 1.750686  0.56  0.40  0.02  0.02 0.0014105761
## 2283 0.2018935 1.554774  0.54  0.40  0.03  0.03 0.0016987029
## 2187 0.3054808 1.281552  0.56  0.34  0.04  0.06 0.0017090884
## 2286 0.3584588 1.554774  0.60  0.34  0.02  0.04 0.0018413904
```

```
ThSim = ThreshSimQuantFnc(gen, quantSteps=0.01, nTraits=2, FieldFreq=FieldFreq,
                  ThNamesT1 = "T1", ThNamesT2 = "T2", phenFnc=phenFnc,
                  min=c(0.05, 1.0), maxT=c(0.45, 1.8))
head(ThSim[order(ThSim$Dev),], 10)
```

```
##             T1       T2 PhenB PhenD PhenG PhenL          Dev
## 9466 0.2793190 1.750686  0.59  0.37  0.02  0.02 2.374959e-05
## 9367 0.2793190 1.644854  0.58  0.37  0.02  0.03 2.946160e-05
## 9465 0.2533471 1.750686  0.58  0.39  0.02  0.02 1.177810e-04
## 9268 0.2793190 1.554774  0.57  0.37  0.02  0.04 1.432591e-04
## 9366 0.2533471 1.644854  0.57  0.38  0.02  0.03 1.604955e-04
## 9368 0.3054808 1.644854  0.59  0.36  0.02  0.03 2.757062e-04
## 9467 0.3054808 1.750686  0.60  0.37  0.02  0.02 3.119407e-04
## 9267 0.2533471 1.554774  0.56  0.38  0.02  0.04 3.171224e-04
## 9269 0.3054808 1.554774  0.58  0.36  0.02  0.04 3.455407e-04
## 9169 0.2793190 1.475791  0.57  0.36  0.03  0.04 3.625329e-04
```

```
print(paste0("Within one order of magnitude: of best combination:  ", length(ThSim$Dev<=min(ThSim$Dev)*
```

```
## [1] "Within one order of magnitude: of best combination:  192"
```

A reasonable fit to field morph frequencies can be achieved with thresholds of $T_1 = 0.28$, $T_2 = 1.75$. Note slight sampling variation between runs.

```
ThreshT1 = 0.28
ThreshT2 = 1.75
gen$Phenotype = phenFnc(gen, ThreshT1, ThreshT2, NA)
par(mfrow=c(1,2))
hist(gen$GV1, nclass=30, main="Trait for color of dorsal side")
abline(v=ThreshT1, lwd=2)
text(c(-3,3), 18000, c("Brown", "Green"))
hist(gen$GV2, nclass=30, main="Trait for color of lateral side")
abline(v=ThreshT2, lwd=2)
text(c(-3,3), 18000, c("Brown", "Green"))
```

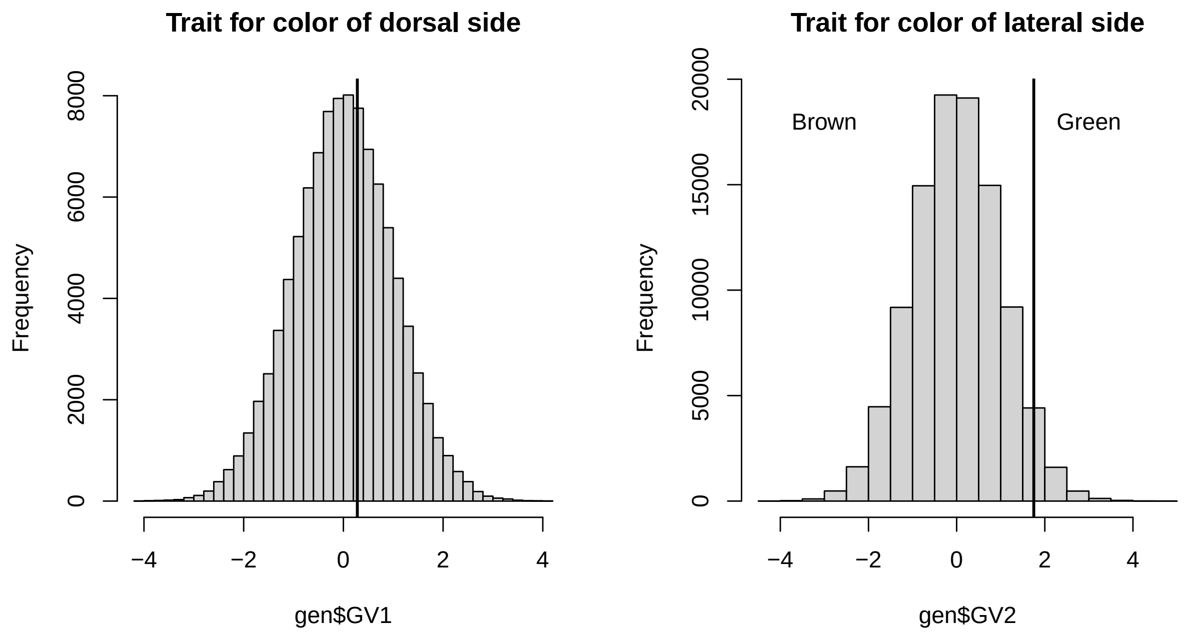


```
rbind(Simulated=round(table(gen$Phenotype)/nrow(gen),2),
      Observed=round(FieldFreq,2))
```

```
##               B    D    G    L
## Simulated 0.59 0.37 0.02 0.02
## Observed  0.58 0.38 0.03 0.02
```

Now parents are sampled from this population according to the implemented mating design. Offspring genotypic values are sampled from parental genotypes, offspring phenotype determined and the results tabulated. The process is repeated *nruns* times. Simulation results are plotted (dots) along with the observed number of offspring (bars) per mating combination. The following table shows the proportion of simulations that are as extreme as or more extreme than the observed number of offspring (a p value for the probability that the observations arose by sampling variation given the mating design). Only mating combinations in which at least one offspring morph shows $p < 0.05$ are shown.

```
nruns=1000
res = simQuantFnc(gen, dat, FemaleUq, MaleUq, nruns, phenFnc, ThreshT1, ThreshT2, NA)
pVals = plotFnc(obs, res)
```

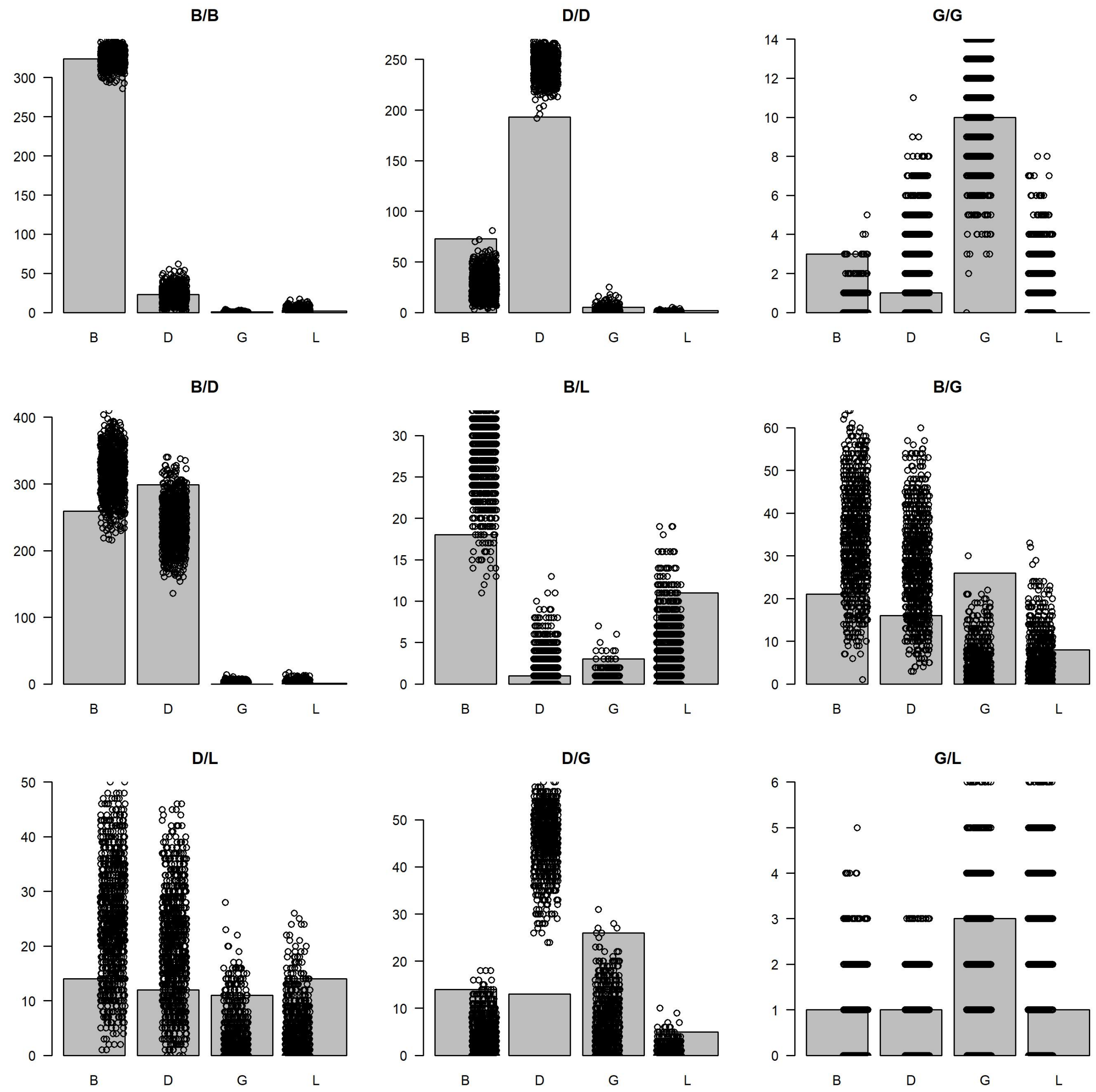


```
evalTab["Model 13", 6:11] = modevalFnc(obs, res)

## Model fit in offspring numbers and relative improvement to null (uniform) model
## Absolute deviations:           424 (69.3%)
## Sq root of squared deviations: 123 (70.8%)
## Sum of squared deviations:     15154 (91.5%)

pVals[apply(pVals[,5:8],1,min)<0.05, 5:12]

##        pB    pD    pG    pL Bsim0 Dsim0 Gsim0 Lsim0
## D/D 0.001 0.001 0.072 0.028 FALSE FALSE FALSE FALSE
## G/G 0.016 0.403 0.500 0.542 FALSE FALSE FALSE FALSE
## B/D 0.056 0.047 0.437 0.595 FALSE FALSE FALSE FALSE
## B/L 0.039 0.560 0.016 0.058 FALSE FALSE FALSE FALSE
## B/G 0.124 0.190 0.001 0.258 FALSE FALSE FALSE FALSE
```

```
## D/G 0.014 0.000 0.006 0.018 FALSE FALSE FALSE FALSE
```

The model gives a poor fit to most mating combinations. For example, the model underpredicts the number of B offspring for D/D and G/G matings and it overpredicts the number of D offspring for D/D and D/G matings. Furthermore, it underpredicts G offspring for B/G and D/G matings and underpredicts L for D/L and D/G matings. The model is, therefore, incompatible with the observed data.

Model 14: Three-trait models

Instead of being explained by a single 'trait' or two 'traits', morph could be considered to consist of three 'traits'. Hence, there could be many loci influencing three traits with the combination of three trait-value thresholds determining morph identities. We here assume five independent loci (or haplotypes) with equal effect sizes per trait to allow for some sampling variation (though there could be many more loci involved). First, we generate a large pool of individuals (controlled by *nind*), each with 30 alleles (2 at 5 loci for 3 traits). Genotypic values (*GV1*, *GV2*, and *GV3*) are calculated as the average across alleles.

```
nind=100000
loci = data.frame(matrix(rnorm(nind*30, 0, sqrt(10)), ncol=30), Phenotype=NA)
gen = data.frame(loci, GV1=scale(rowMeans(loci[,1:10])),
                       GV2=scale(rowMeans(loci[,11:20])),
                       GV3=scale(rowMeans(loci[,21:30])))
gen$Phenotype = NA
phenFnc = function(mydat, ThreshT1, ThreshT2, ThreshT3) {
  Phenotype = NA
  Phenotype[is.na(Phenotype) & mydat$GV1 < ThreshT1] = "B"
  Phenotype[is.na(Phenotype) & mydat$GV2 >= ThreshT2 & mydat$GV3 >= ThreshT3] = "G"
  Phenotype[is.na(Phenotype) & mydat$GV2 < ThreshT2 & mydat$GV3 >= ThreshT3] = "D"
  Phenotype[is.na(Phenotype) & mydat$GV2 >= ThreshT2 & mydat$GV3 < ThreshT3] = "L"
  Phenotype[is.na(Phenotype) & mydat$GV2 < ThreshT2 & mydat$GV3 < ThreshT3] = "B"
  return(Phenotype)
}
```

We use simulations to find genotypic thresholds that fit field morph frequencies. The first round scans in allele frequency steps of 5% to explore the full range of possibilities, the second round scans in steps of 2% and the third in steps of 1% to test for the best combination in a restricted range. The output shows a column *Dev* with the sum of squared deviation between predictions and field morph frequencies, where lower values indicate a better fit (the top 10 results are shown).

```
ThSim = ThreshSimQuantFnc(gen, quantSteps=0.05, nTraits=3, FieldFreq=FieldFreq,
                 ThNamesT1 = "B", ThNamesT2 = "G", ThNamesT3 = "L", phenFnc=phenFnc)
head(ThSim[order(ThSim$Dev),], 10)
```

```
##                B        G          L PhenB PhenD PhenG PhenL          Dev
## 3595 -0.8416212 1.644854  0.0000000  0.58  0.38  0.02  0.02 2.897812e-05
## 1776 -0.1256613 1.281552 -0.6744898  0.57  0.37  0.04  0.01 6.672447e-05
## 334   0.1256613 1.281552 -1.6448536  0.57  0.38  0.04  0.00 1.570101e-04
## 1435  0.0000000 1.644854 -0.8416212  0.59  0.38  0.02  0.00 2.033454e-04
## 3955 -1.0364334 1.644854  0.1256613  0.59  0.37  0.02  0.02 2.290568e-04
## 714   0.1256613 1.644854 -1.2815516  0.59  0.38  0.02  0.00 2.312604e-04
## 1055  0.0000000 1.281552 -1.0364334  0.57  0.38  0.04  0.01 2.350458e-04
## 695   0.1256613 1.281552 -1.2815516  0.59  0.36  0.04  0.00 2.493858e-04
## 2876 -0.3853205 1.644854 -0.2533471  0.60  0.37  0.02  0.01 2.561485e-04
## 1795 -0.1256613 1.644854 -0.6744898  0.58  0.39  0.02  0.01 2.669945e-04
```

```
ThSim = ThreshSimQuantFnc(gen, quantSteps=0.02, nTraits=3, FieldFreq=FieldFreq,
                 ThNamesT1 = "B", ThNamesT2 = "G", ThNamesT3 = "L", phenFnc=phenFnc,
                 minT=c(-1.3, 1.2, -1.7), maxT=c(0.2, 1.7, 0.2))
head(ThSim[order(ThSim$Dev),], 10)
```

```
##                 B        G           L PhenB PhenD PhenG PhenL          Dev
## 57489 -0.70630256 1.554774 -0.05015358  0.58  0.37  0.02  0.02 4.804078e-06
## 35840 -0.20189348 1.405072 -0.52440051  0.58  0.37  0.03  0.01 4.899030e-06
## 55089 -0.64334541 1.554774 -0.10043372  0.58  0.38  0.02  0.02 6.775960e-06
```

```
## 28639 -0.10043372 1.405072 -0.70630256  0.58  0.38  0.03  0.01 7.191993e-06
## 21438  0.00000000 1.405072 -0.91536509  0.58  0.38  0.03  0.01 7.950735e-06
## 11787  0.10043372 1.281552 -1.28155157  0.58  0.37  0.04  0.00 1.559013e-05
## 38240 -0.25334710 1.405072 -0.46769880  0.58  0.38  0.03  0.01 1.565455e-05
## 16637  0.05015358 1.405072 -1.08031934  0.58  0.38  0.03  0.01 1.710147e-05
## 16588  0.05015358 1.281552 -1.08031934  0.58  0.37  0.04  0.01 1.913248e-05
## 26239 -0.05015358 1.405072 -0.77219321  0.59  0.37  0.03  0.01 1.983504e-05
```

```
ThSim = ThreshSimQuantFnc(gen, quantSteps=0.01, nTraits=3, FieldFreq=FieldFreq,
                  ThNamesT1 = "B", ThNamesT2 = "G", ThNamesT3 = "L", phenFnc=phenFnc,
                  minT=c(-1.3, 1.2, -1.7), maxT=c(0.2, 1.7, 0.2))
head(ThSim[order(ThSim$Dev),], 10)
```

```
##                  B        G           L PhenB PhenD PhenG PhenL          Dev
## 48071   0.15096922 1.281552 -1.64485363  0.58  0.38  0.04  0.00 3.154368e-07
## 136375  0.05015358 1.340755 -1.08031934  0.58  0.38  0.04  0.01 3.956501e-07
## 352180 -0.33185335 1.475791 -0.35845879  0.58  0.38  0.03  0.02 4.004348e-07
## 155976  0.02506891 1.340755 -0.99445788  0.58  0.37  0.04  0.01 6.991462e-07
## 361980 -0.35845879 1.475791 -0.33185335  0.58  0.38  0.03  0.02 1.028715e-06
## 460078 -0.67448975 1.554774 -0.07526986  0.58  0.38  0.02  0.02 1.082999e-06
## 67672   0.12566135 1.281552 -1.47579103  0.58  0.38  0.04  0.00 1.133128e-06
## 430679 -0.55338472 1.554774 -0.15096922  0.58  0.38  0.02  0.02 1.305033e-06
## 420879 -0.52440051 1.554774 -0.17637416  0.58  0.38  0.02  0.02 1.385760e-06
## 224678 -0.07526986 1.405072 -0.73884685  0.58  0.38  0.03  0.01 1.403668e-06
```

```
print(paste0("Within one order of magnitude: of best combination:  ", length(ThSim$Dev<=min(ThSim$Dev)*[illegible]
```

```
## [1] "Within one order of magnitude: of best combination:  17808"
```

A reasonable fit to field morph frequencies can be achieved with thresholds of $T_B = 0.08$, $T_G = 1.34$, $p_L = -1.17$. Note slight sampling variation between runs.

```
ThreshT1 = 0.08
ThreshT2 = 1.34
ThreshT3 = -1.17
gen$Phenotype = phenFnc(gen, ThreshT1, ThreshT2, ThreshT3)
par(mfrow=c(1,3))
hist(gen$GV1, nclass=30, main="Trait for potential to be green")
abline(v=ThreshT1, lwd=2)
text(c(-3,3), 18000, c("Brown", "Green"))
hist(gen$GV2, nclass=30, main="Trait for color of dorsal side")
abline(v=ThreshT2, lwd=2)
text(c(-3,3), 18000, c("Brown", "Green"))
hist(gen$GV3, nclass=30, main="Trait for color of lateral side")
abline(v=ThreshT3, lwd=2)
text(c(-3,3), 18000, c("Brown", "Green"))
```

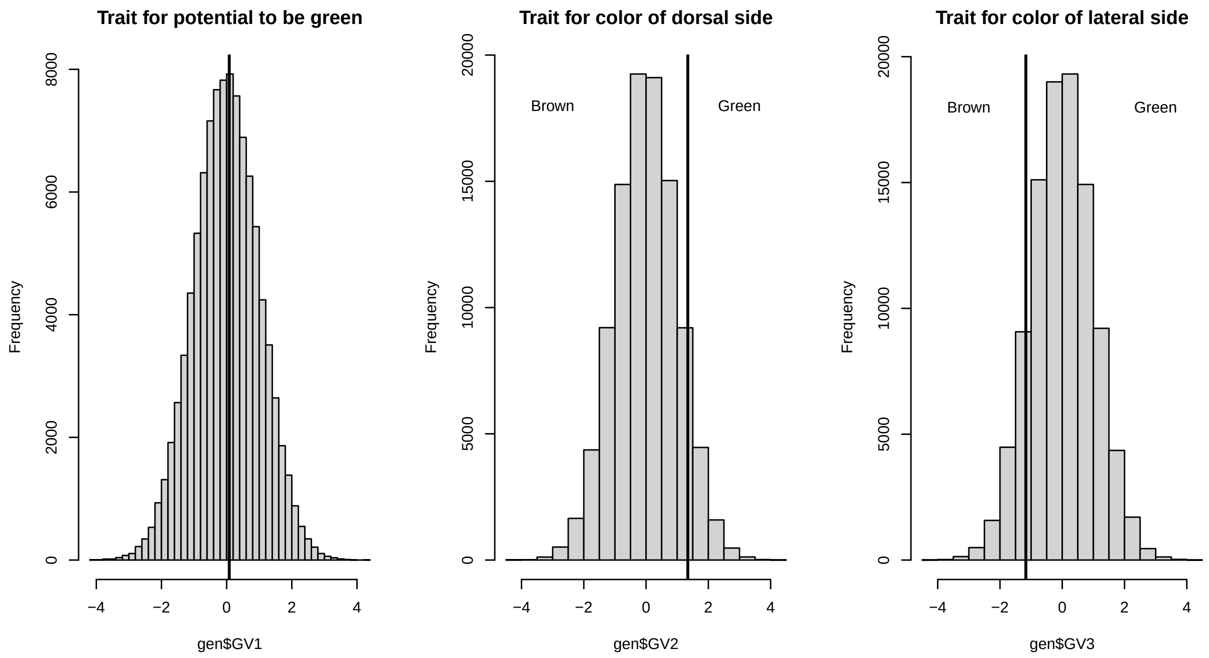


```
print("Field morph frequencies")
```

```
## [1] "Field morph frequencies"
```

```
rbind(Simulated=round(table(gen$Phenotype)/nrow(gen),2),
      Observed=round(FieldFreq,2))
```

```
##              B    D    G    L
## Simulated 0.58 0.37 0.04 0.00
## Observed  0.58 0.38 0.03 0.02
```

Now parents are sampled from this population according to the implemented mating design. Offspring genotypic values are sampled from parental genotypes, offspring phenotype determined and the results tabulated. The process is repeated *nruns* times. Simulation results are plotted (dots) along with the observed number of offspring (bars) per mating combination. The following table shows the proportion of simulations that are as extreme or more extreme than the observed number of offspring (a p value for the probability that the observations arose by sampling variation given the mating design). Only mating combinations in which at least one offspring morph shows $p < 0.05$ are shown.

```
nruns=1000
res = simQuantFnc(gen, dat, FemaleUq, MaleUq, nruns, phenFnc, ThreshT1, ThreshT2, ThreshT3)
pVals = plotFnc(obs, res)
```

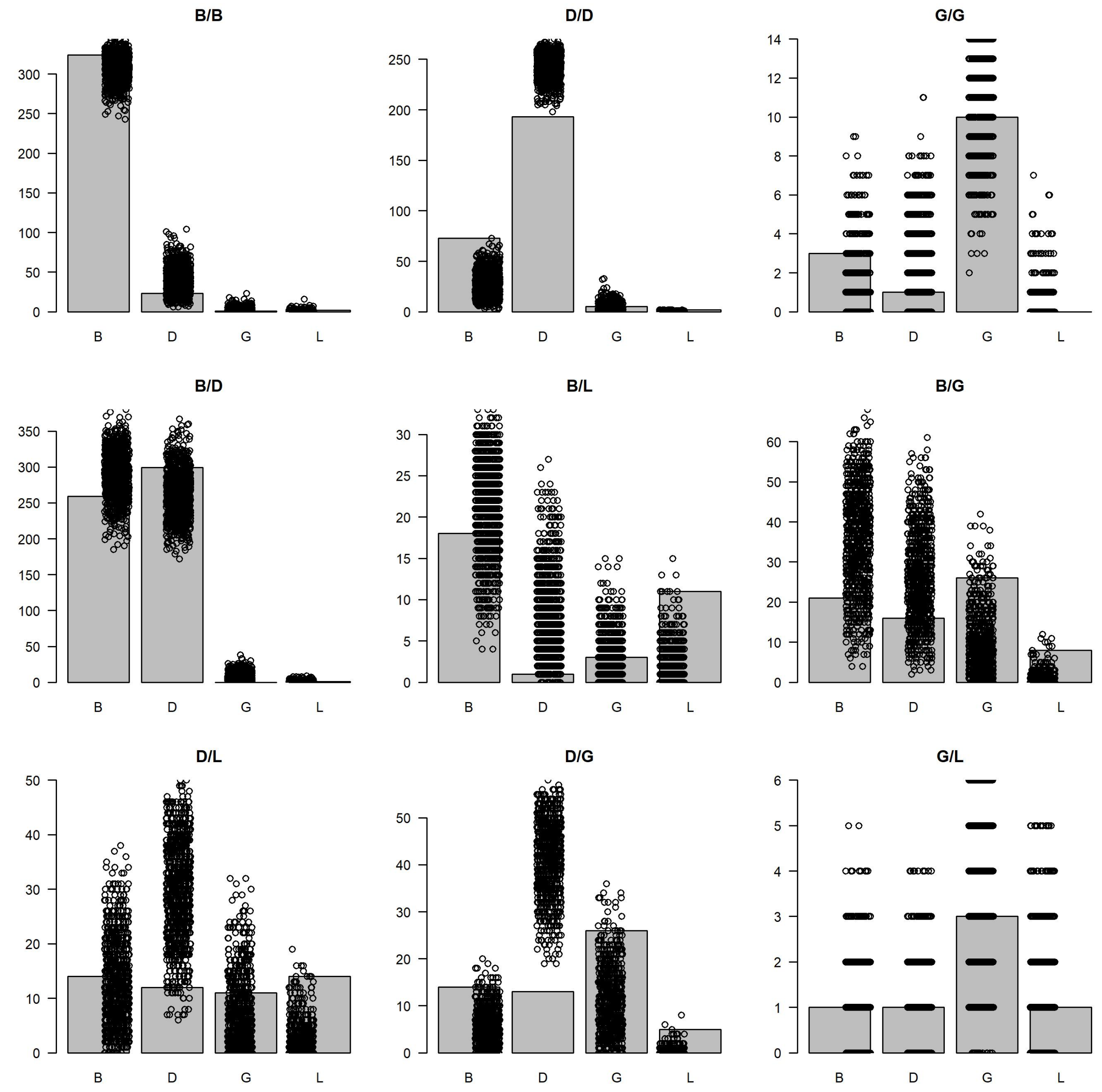


```
evalTab["Model 14", 6:11] = modevalFnc(obs, res)
```

```
## Model fit in offspring numbers and relative improvement to null (uniform) model
## Absolute deviations:           382 (72.3%)
## Sq root of squared deviations: 101 (76%)
## Sum of squared deviations:     10208 (94.3%)
```

```
pVals[apply(pVals[,5:8],1,min)<0.05, 5:12]
```

```
##        pB    pD    pG    pL Bsim0 Dsim0 Gsim0 Lsim0
## D/D 0.001 0.000 0.246 0.010 FALSE FALSE FALSE FALSE
## B/D 0.171 0.126 0.023 0.289 FALSE FALSE FALSE FALSE
## B/L 0.339 0.065 0.365 0.008 FALSE FALSE FALSE FALSE
## B/G 0.143 0.226 0.039 0.008 FALSE FALSE FALSE FALSE
## D/L 0.426 0.030 0.221 0.009 FALSE FALSE FALSE FALSE
```

```
## D/G 0.028 0.000 0.023 0.003 FALSE FALSE FALSE FALSE
```

The model gives a poor fit to several mating combinations. For example, the model underpredicts the number of B offspring for D/D matings and it overpredicts the number of D offspring for D/D and D/G matings. Furthermore, it underpredicts G offspring for B/G and D/G matings and underpredicts L form B/L, B/G, D/L and D/G matings. The model is, therefore, incompatible with the observed data.

Conclusions

The following table shows a summary of the simulation results. The column *AbsDev* shows the difference in the absolute number between the average of the simulations and observed numbers. The column *SqDev* shows the sum of the squared difference in number between the average of the simulations and observed numbers. The column *SrSqDev* shows the square root of the sum of the squared difference in number between the average of the simulations and observed numbers. The columns *AbsDevRel*, *SqDevRel* and *SrSqDevRel* show the improvement relative to a uniform distribution of offspring numbers.

```
##          AbsDev SqDev SrSqDev AbsDevRel SqDevRel SrSqDevRel
## Model 1     348    78    6074     0.748    0.815      0.966
## Model 2     318    76    5776     0.770    0.820      0.968
## Model 3     277    67    4537     0.799    0.840      0.974
## Model 4     206    55    3014     0.851    0.870      0.983
## Model 5     213    47    2223     0.846    0.888      0.988
## Model 6     310    77    5967     0.776    0.817      0.966
## Model 7     148    38    1478     0.893    0.909      0.992
## Model 8     242    60    3557     0.825    0.859      0.980
## Model 9     231    55    2997     0.833    0.870      0.983
## Model 10    189    47    2203     0.863    0.889      0.988
## Model 11    526   140   19471     0.619    0.669      0.891
## Model 12    470   144   20816     0.660    0.658      0.883
## Model 13    424   123   15154     0.693    0.708      0.915
## Model 14    382   101   10208     0.723    0.760      0.943
```

The best-fitting model is one with three loci, two alleles each: One locus G with two alleles (G and b) responsible for the ability to produce green color, with G dominant over b. One locus D with two alleles (D and u) where the recessive allele u suppresses green color dorsally. And one locus L with two alleles (L and n) where the recessive allele n suppresses green color laterally. **The second-fitting model is one with two loci with two and three alleles, respectively**: One locus G with two alleles (G and b) responsible for the ability to produce green color and on locus that controls the body region in whcih green green is expressed. However, the model is structurally similar to the three locus model and gives a slightly less good fit.

There are a few offspring from D/D matings that are not explained by either of these two models. This refers to five G offspring and two L that are not produced by any of the two best-fitting models (2.5% of the 273 offspring from D/D matings). This suggest that there are genetic or environmental modifiers involved that suppress the development of green lateral sides in L allele carriers. The existance of these offspring suggests a role of modifiers. A four locus model with two alleles for the lateral green colour can produce the missing G and L offspring. However, overall the model does not give a better fit than the three-locus model such that it remains open if modifiers have a genetic or environmental origin resulting in incomplete penetrance of the laternal green phenotype.
